# Supplementary figures and images for: GMCL1 controls 53BP1 stability and modulates taxane sensitivity (part 1 of 2)
Source: eLife. 2026 Jan 19;14:RP106730. doi: 10.7554/eLife.106730 (PMC12815461; doi:10.7554/eLife.106730)

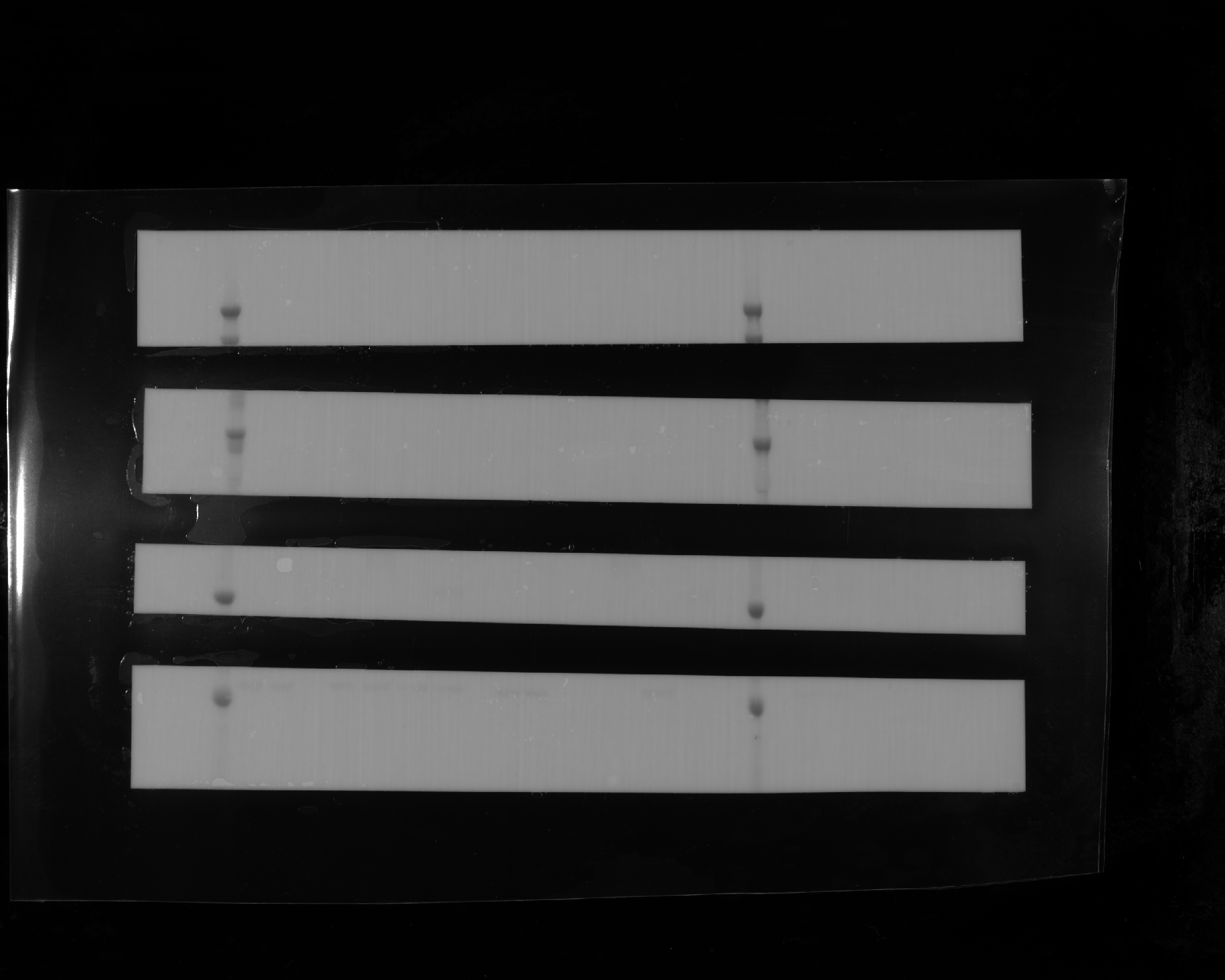

Supplement: Figure 1—source data 1. [file elife-106730-fig1-data1.zip › Figure 1ΓÇösource data 1/Figure 1F/Gmcl1KI_53bp1_cul3_gmcl1_actin_6(Colorimetric).tif]

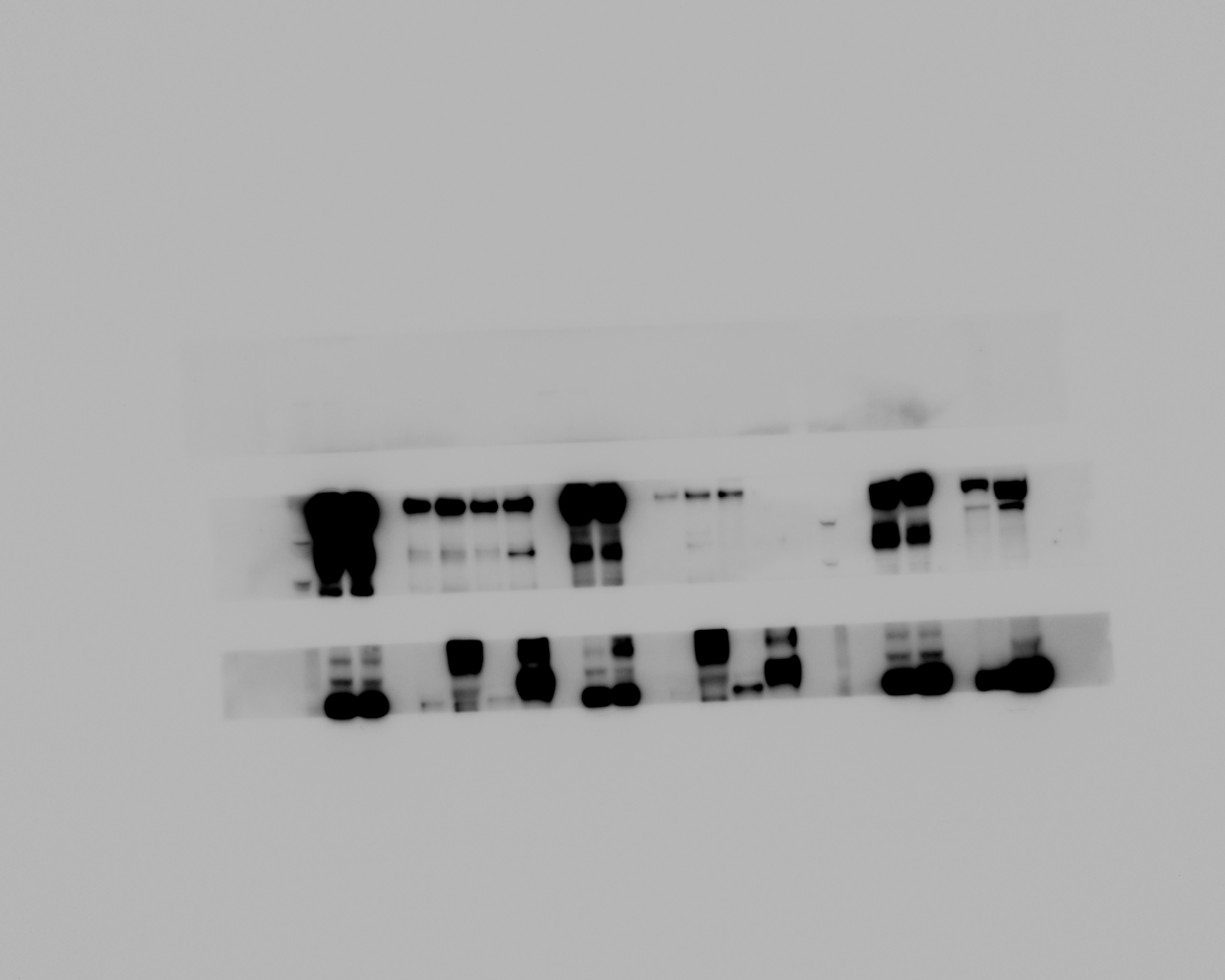

Supplement: Figure 1—source data 1. [file elife-106730-fig1-data1.zip › Figure 1ΓÇösource data 1/Figure 1F/Gmcl1KI_vinculin_usp28_p53_4(Chemiluminescence_Background).tif]

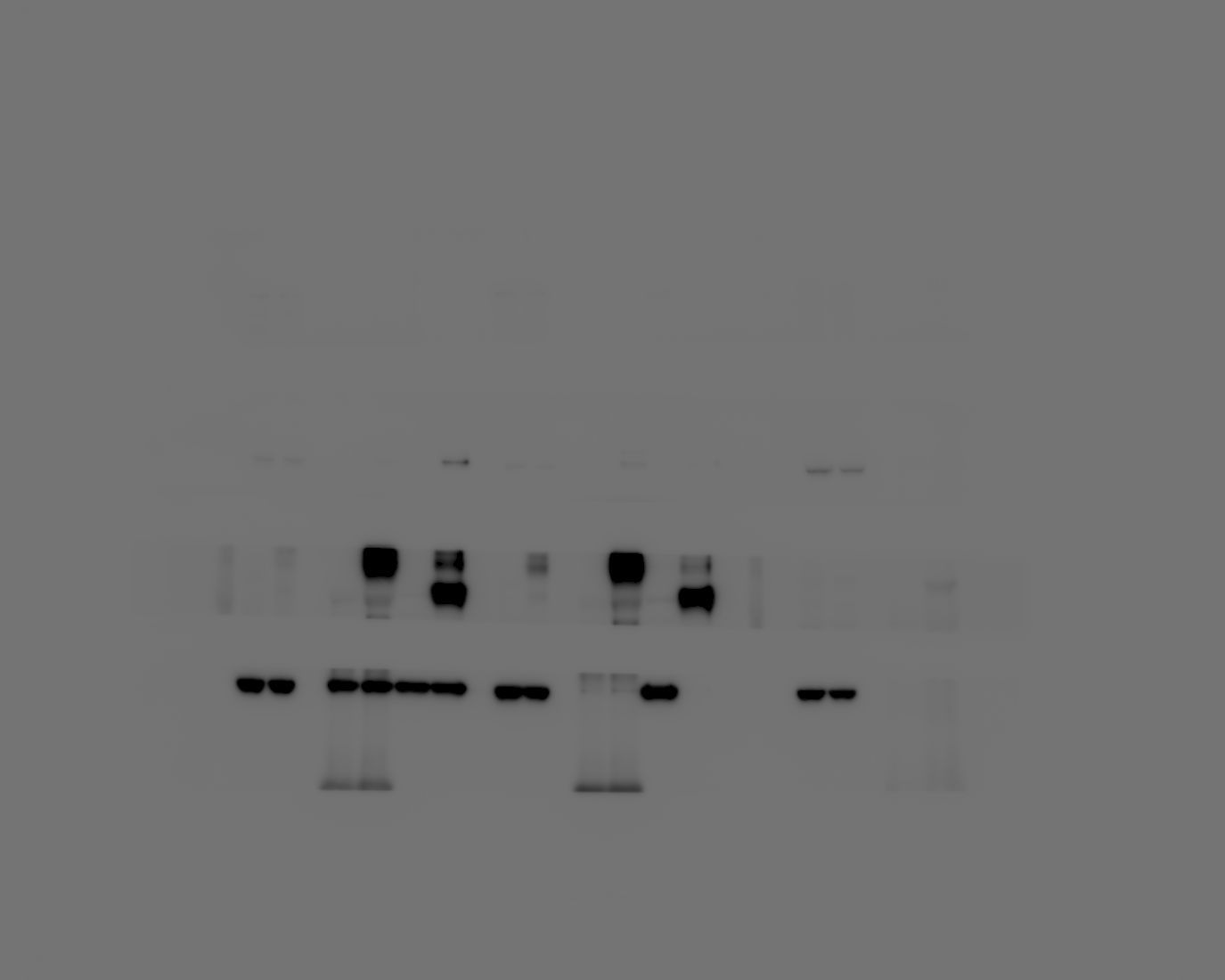

Supplement: Figure 1—source data 1. [file elife-106730-fig1-data1.zip › Figure 1ΓÇösource data 1/Figure 1F/Gmcl1KI_53bp1_cul3_gmcl1_actin_2(Chemiluminescence).png]

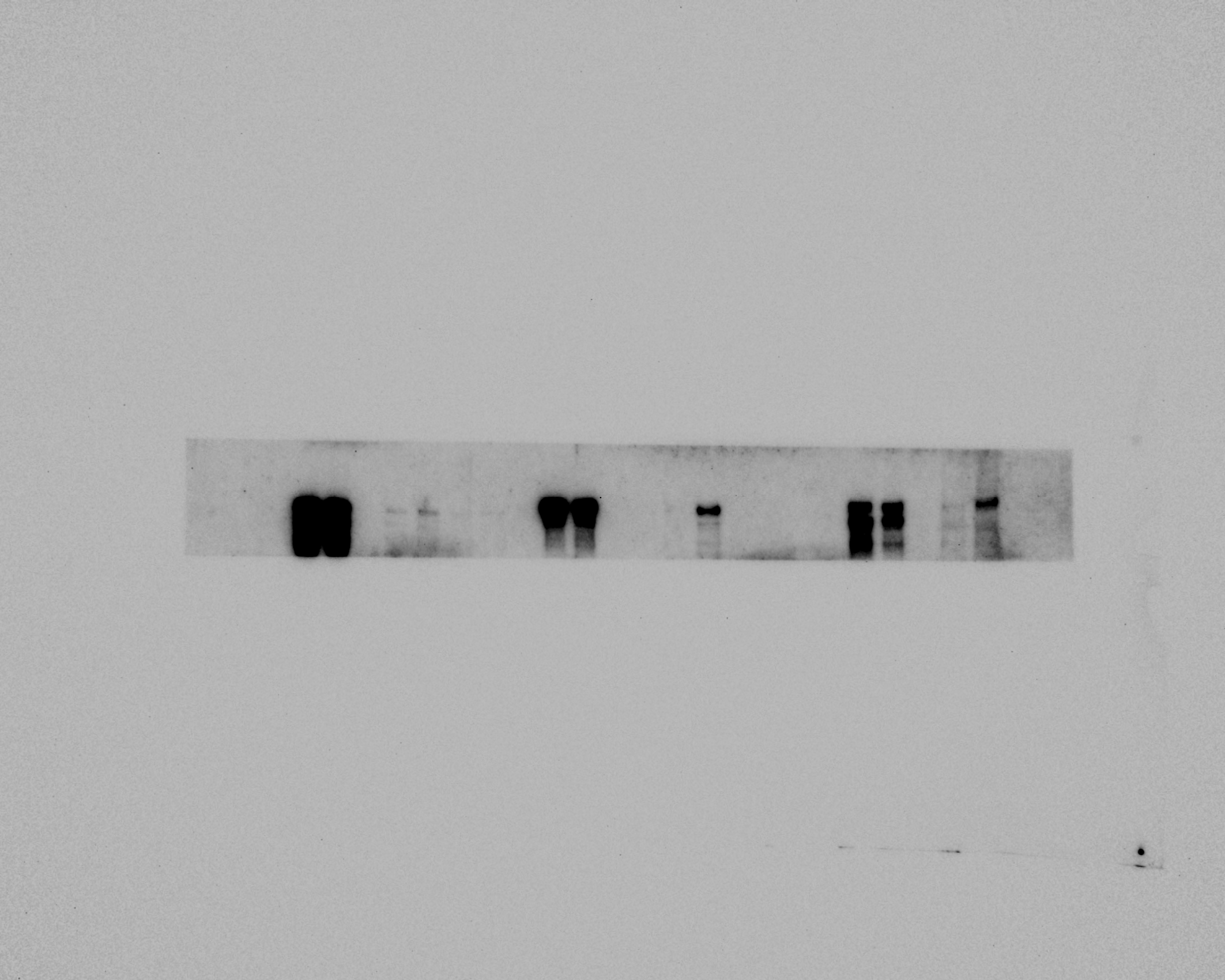

Supplement: Figure 1—source data 1. [file elife-106730-fig1-data1.zip › Figure 1ΓÇösource data 1/Figure 1F/Gmcl1KI_53bp1_5(Chemiluminescence_Background).tif]

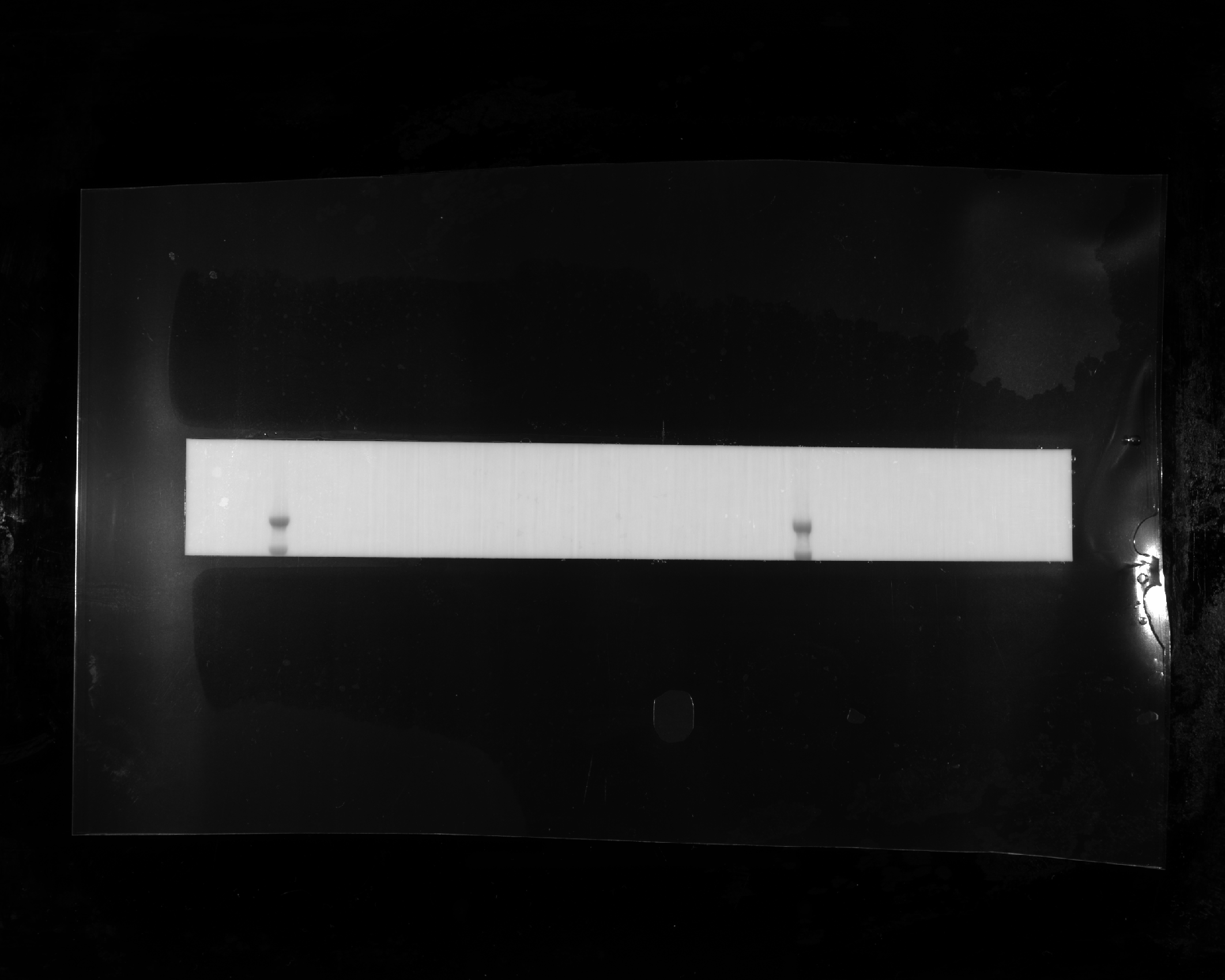

Supplement: Figure 1—source data 1. [file elife-106730-fig1-data1.zip › Figure 1ΓÇösource data 1/Figure 1F/Gmcl1KI_53bp1_6(Colorimetric).tif]

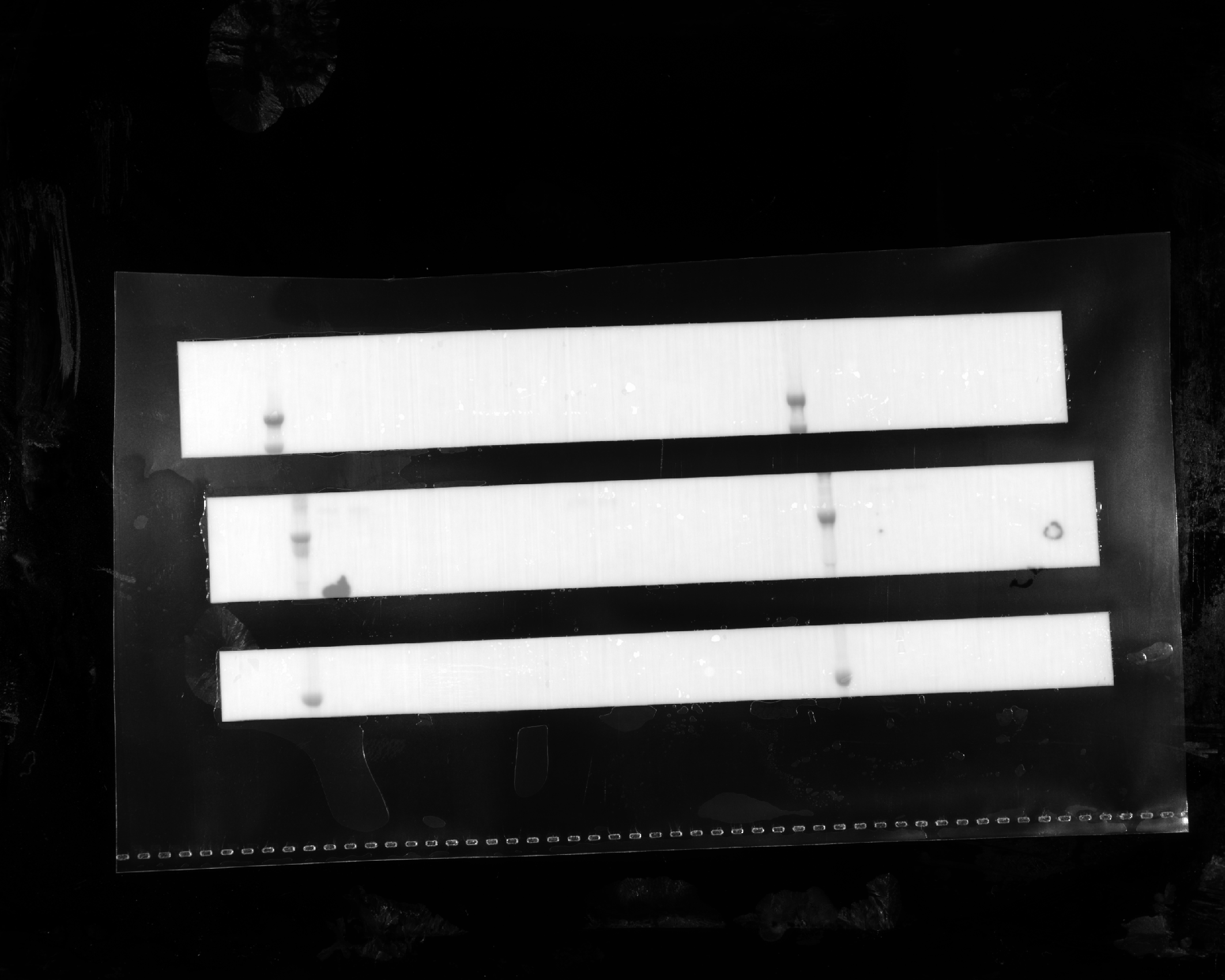

Supplement: Figure 1—source data 1. [file elife-106730-fig1-data1.zip › Figure 1ΓÇösource data 1/Figure 1F/Gmcl1KI_vinculin_usp28_p53_6(Colorimetric).tif]

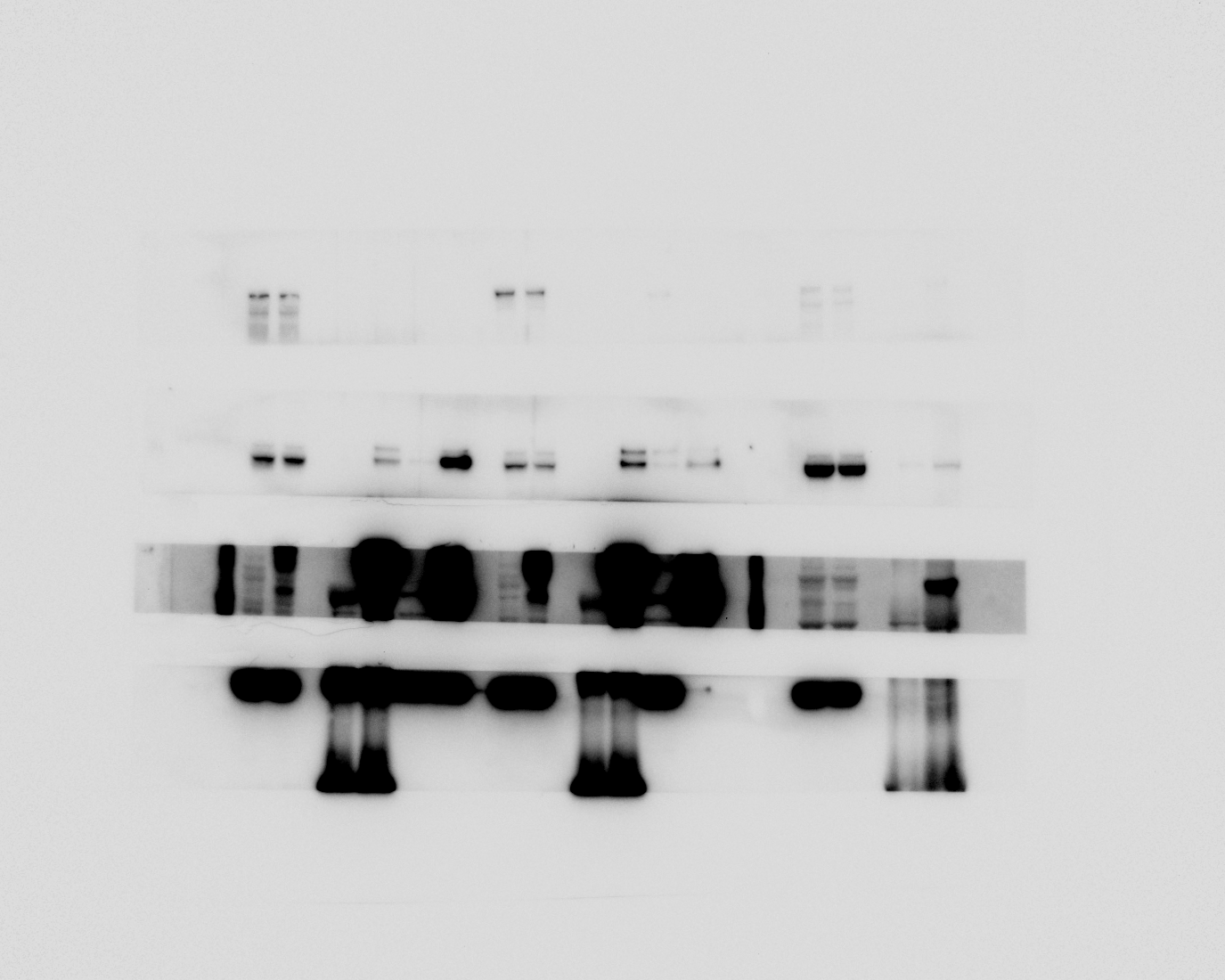

Supplement: Figure 1—source data 1. [file elife-106730-fig1-data1.zip › Figure 1ΓÇösource data 1/Figure 1F/Gmcl1KI_53bp1_cul3_gmcl1_actin_2(Chemiluminescence_Background).raw16.tif]

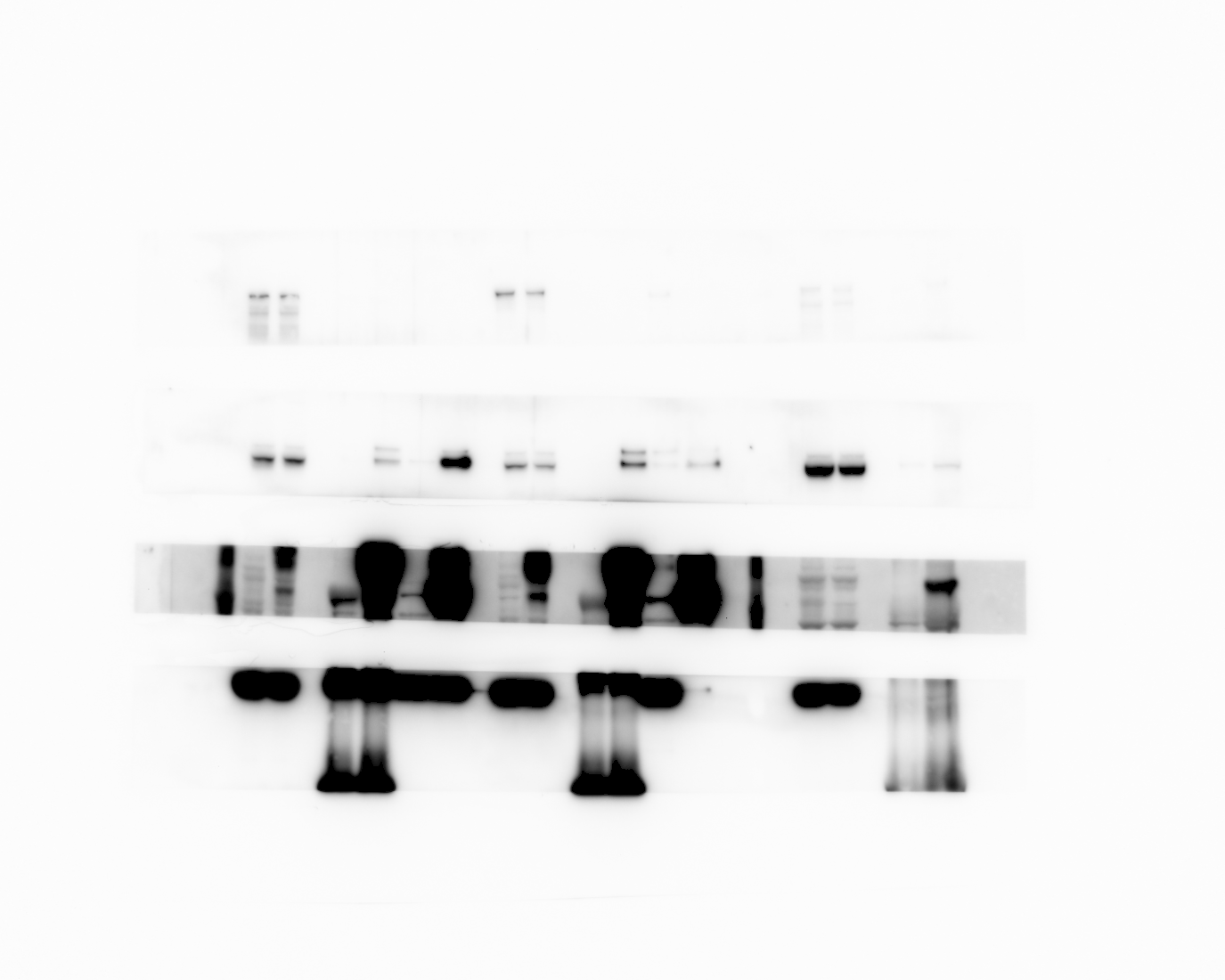

Supplement: Figure 1—source data 1. [file elife-106730-fig1-data1.zip › Figure 1ΓÇösource data 1/Figure 1F/Gmcl1KI_53bp1_cul3_gmcl1_actin_5(Chemiluminescence).raw16.tif]

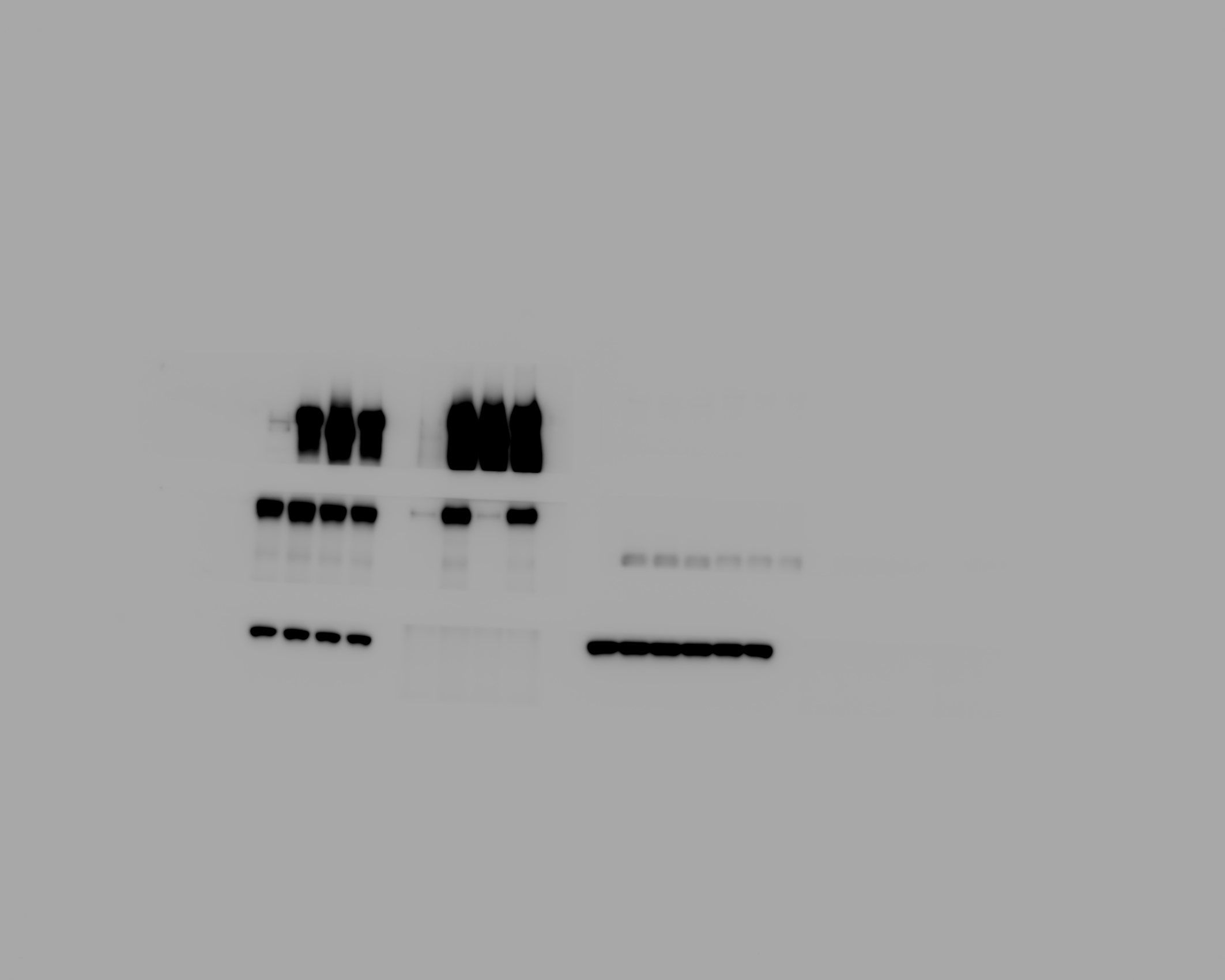

Supplement: Figure 1—source data 1. [file elife-106730-fig1-data1.zip › Figure 1ΓÇösource data 1/Figure 1E/112324-53bp1-Flag-IP_Flag_usp28_actin_53bp1_p53_05(Chemiluminescence_Background).tif]

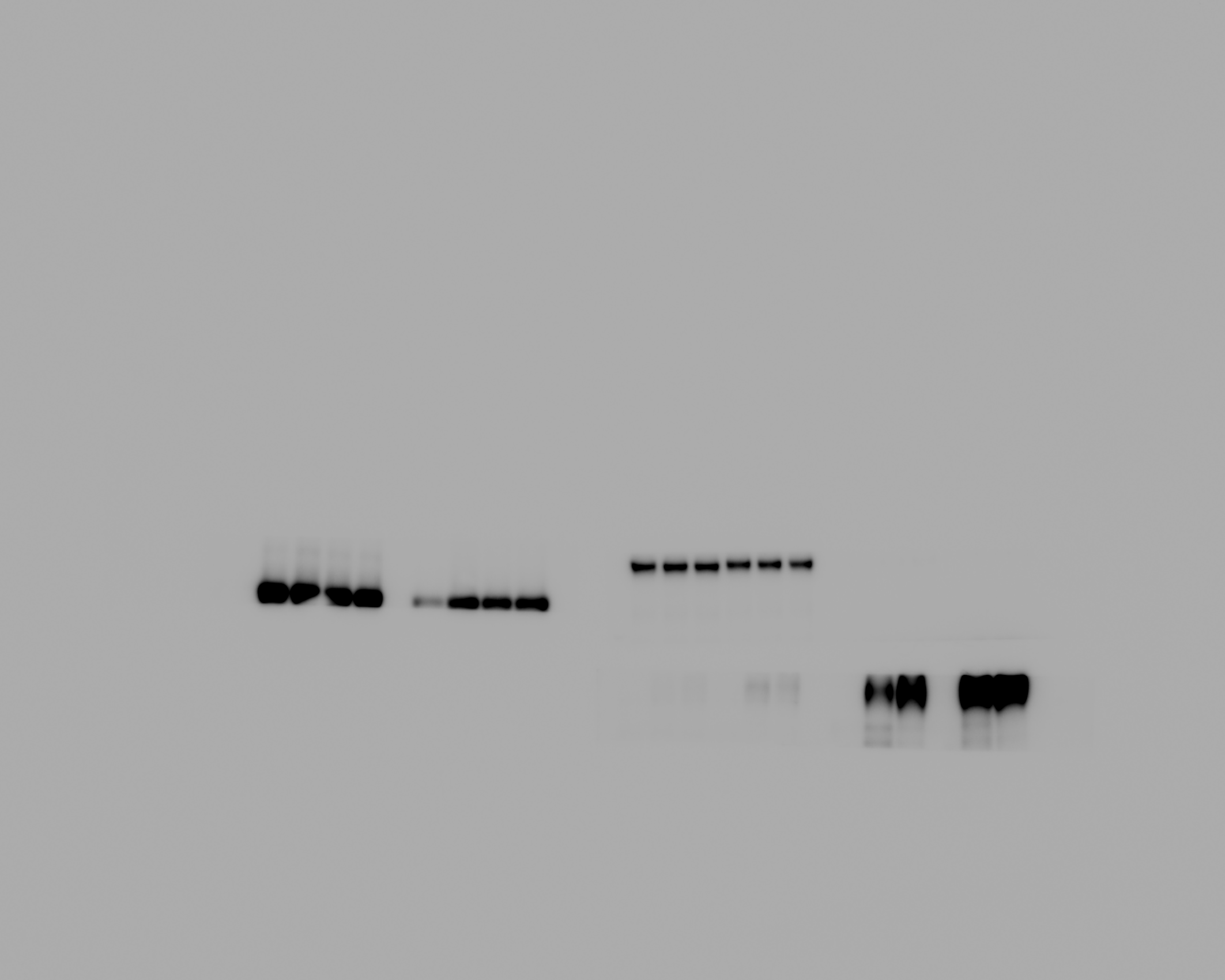

Supplement: Figure 1—source data 1. [file elife-106730-fig1-data1.zip › Figure 1ΓÇösource data 1/Figure 1E/112324-53bp1-IP_p53_usp28_flag_03(Chemiluminescence).tif]

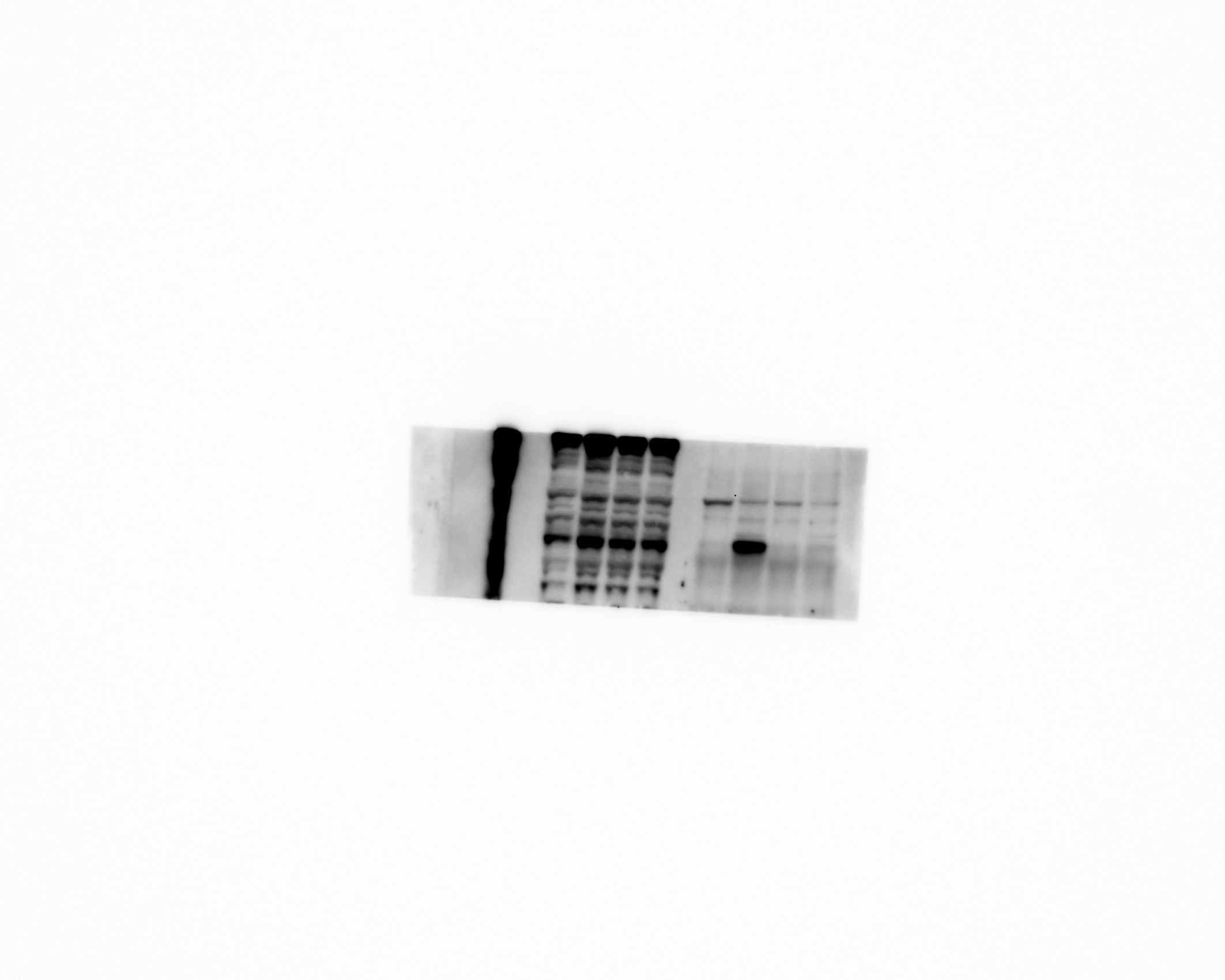

Supplement: Figure 1—source data 1. [file elife-106730-fig1-data1.zip › Figure 1ΓÇösource data 1/Figure 1E/112324-Rerun-53bp1-IP_gmcl1_4(Chemiluminescence).tif]

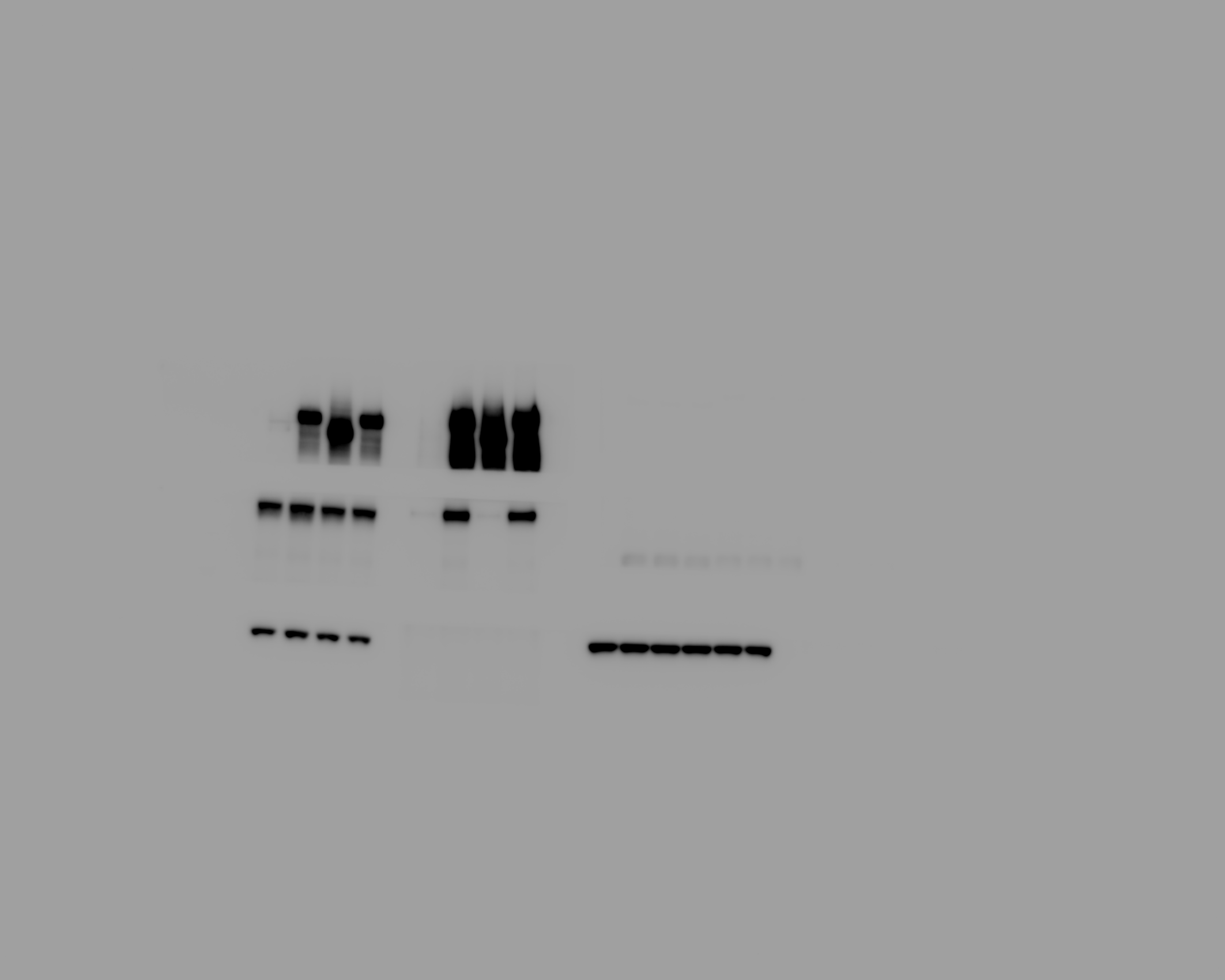

Supplement: Figure 1—source data 1. [file elife-106730-fig1-data1.zip › Figure 1ΓÇösource data 1/Figure 1E/112324-53bp1-Flag-IP_Flag_usp28_actin_53bp1_p53_03(Chemiluminescence_Background).tif]

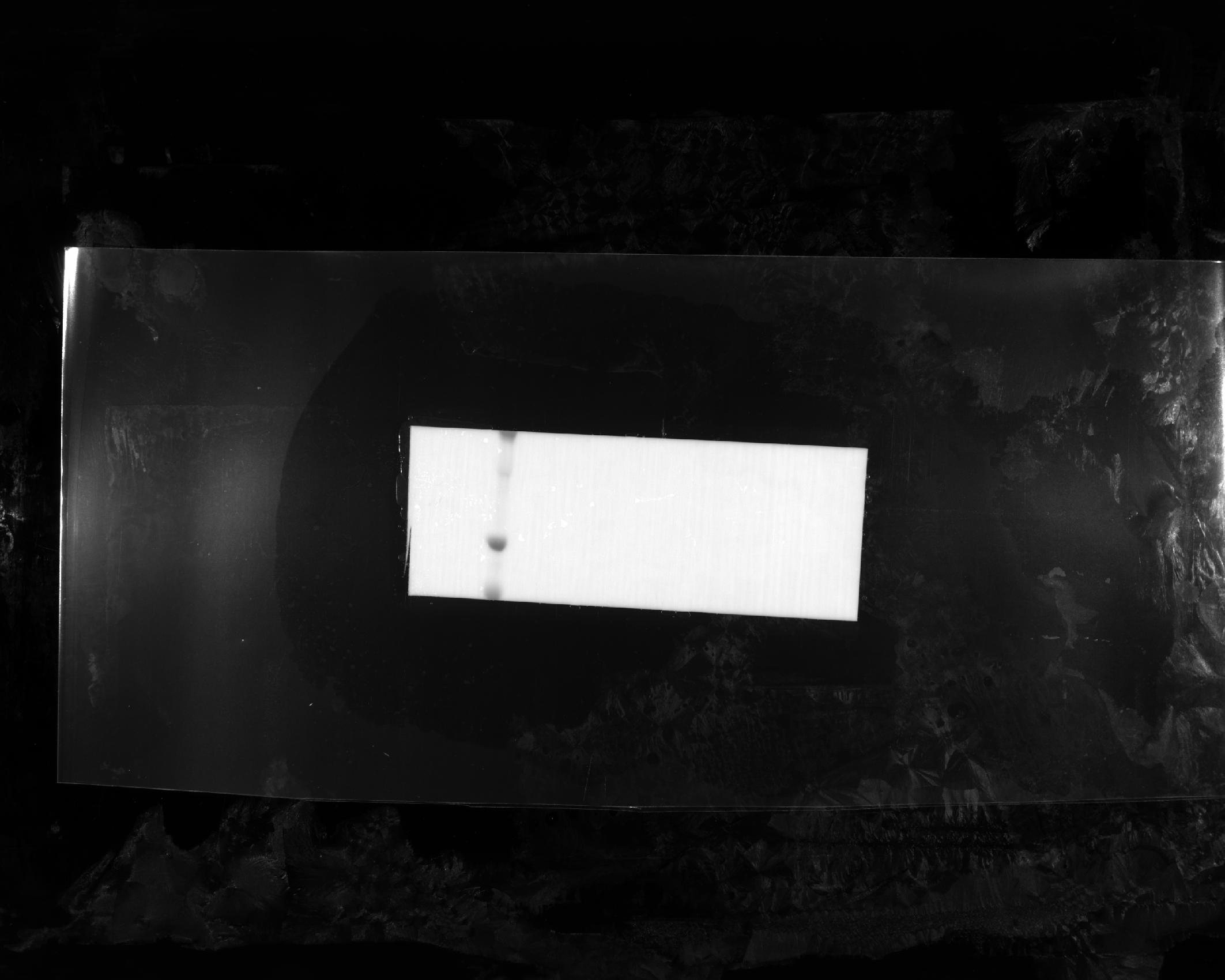

Supplement: Figure 1—source data 1. [file elife-106730-fig1-data1.zip › Figure 1ΓÇösource data 1/Figure 1E/112324-Rerun-53bp1-IP_gmcl1_6(Colorimetric).tif]

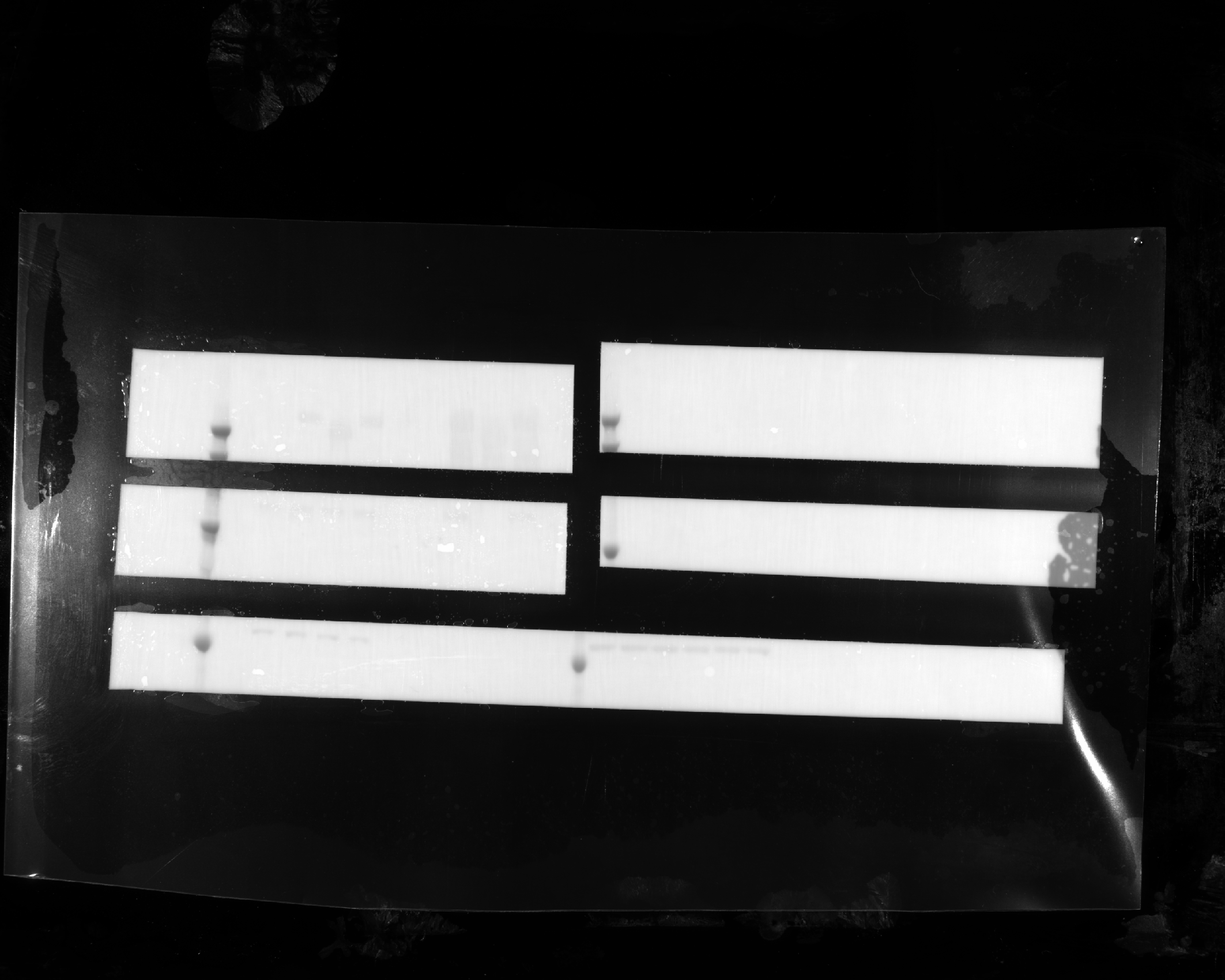

Supplement: Figure 1—source data 1. [file elife-106730-fig1-data1.zip › Figure 1ΓÇösource data 1/Figure 1E/112324-53bp1-Flag-IP_Flag_usp28_actin_53bp1_p53_11(Colorimetric).tif]

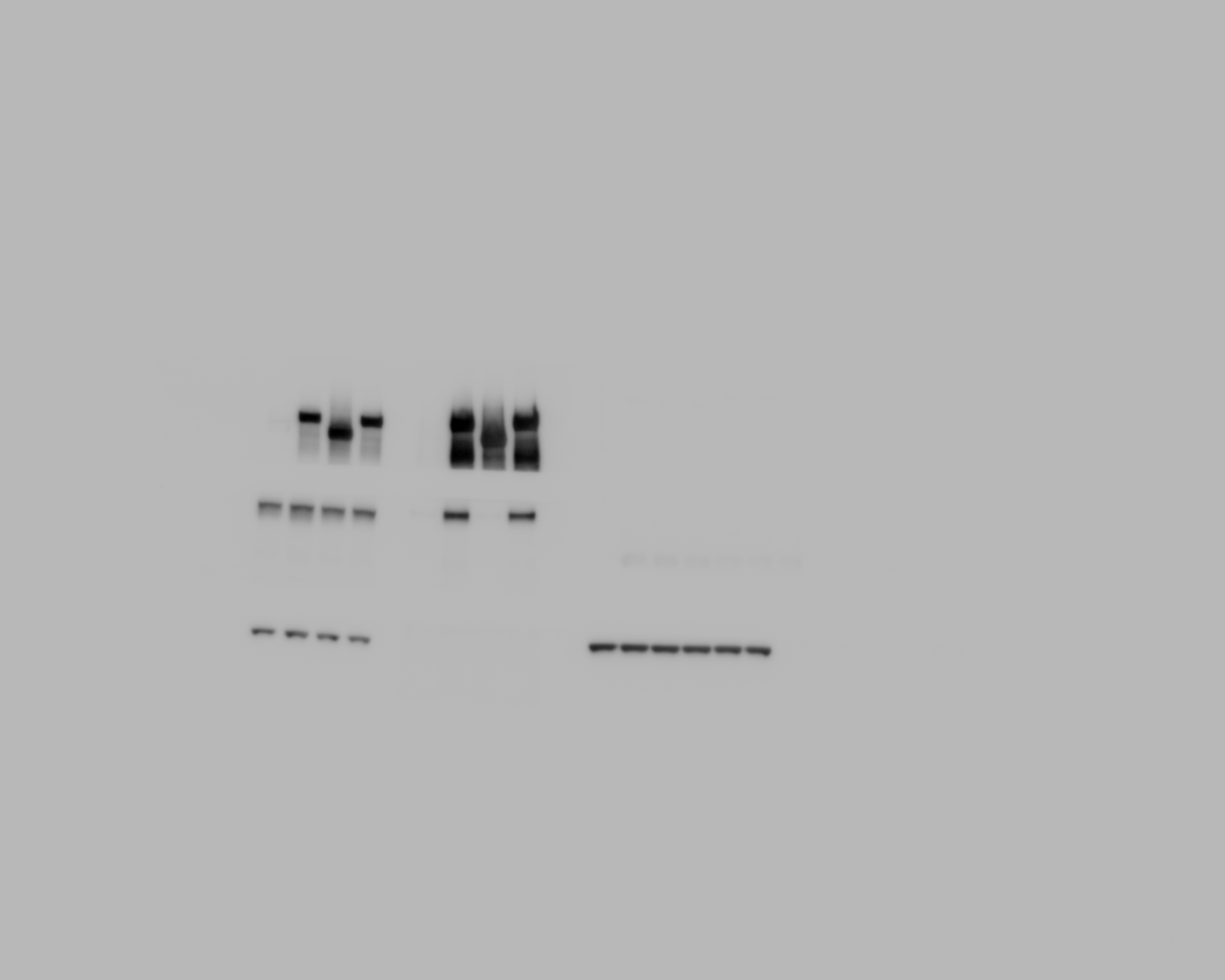

Supplement: Figure 1—source data 1. [file elife-106730-fig1-data1.zip › Figure 1ΓÇösource data 1/Figure 1E/112324-53bp1-Flag-IP_Flag_usp28_actin_53bp1_p53_02(Chemiluminescence_Background).tif]

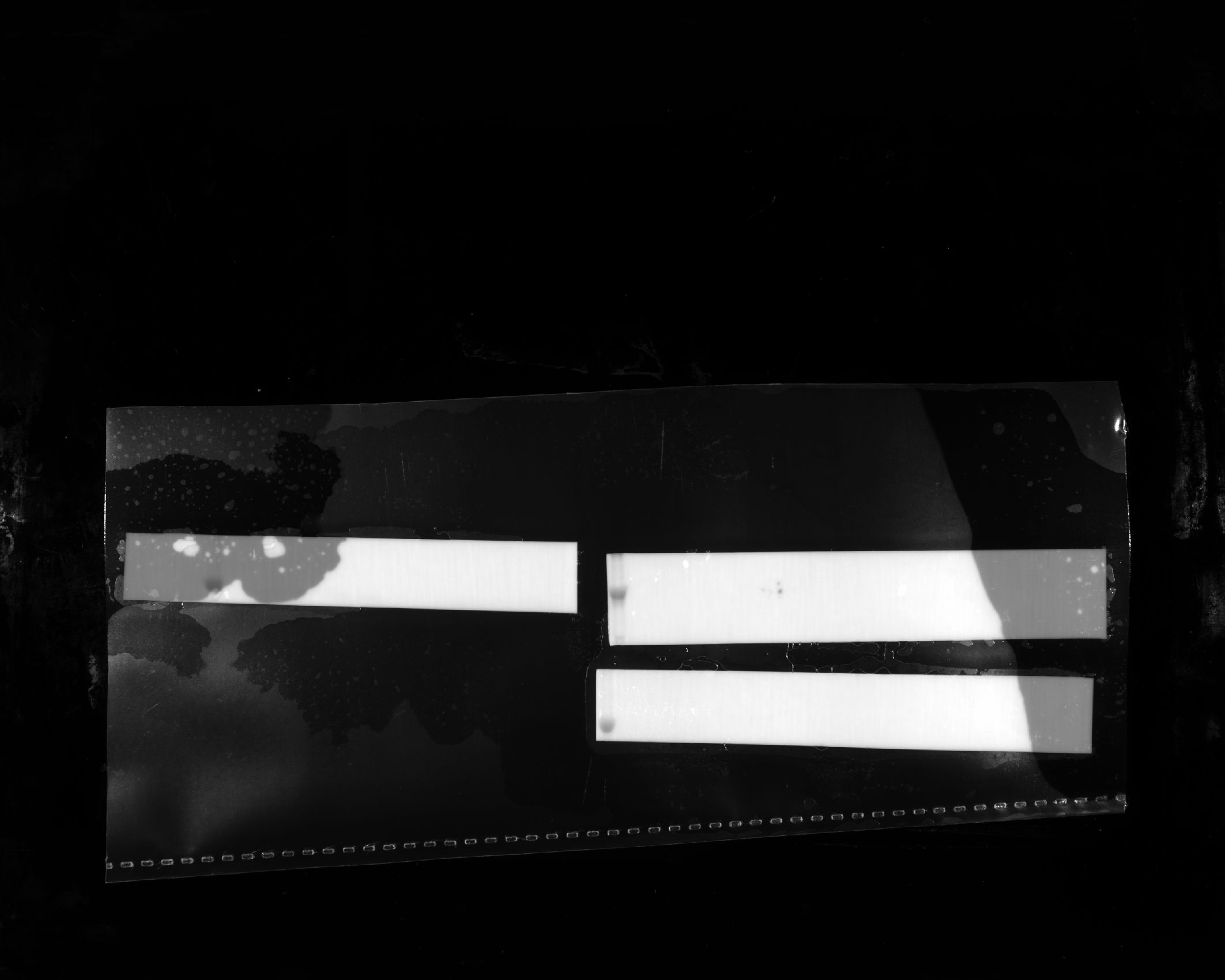

Supplement: Figure 1—source data 1. [file elife-106730-fig1-data1.zip › Figure 1ΓÇösource data 1/Figure 1E/112324-53bp1-IP_p53_usp28_flag_11(Colorimetric).tif]

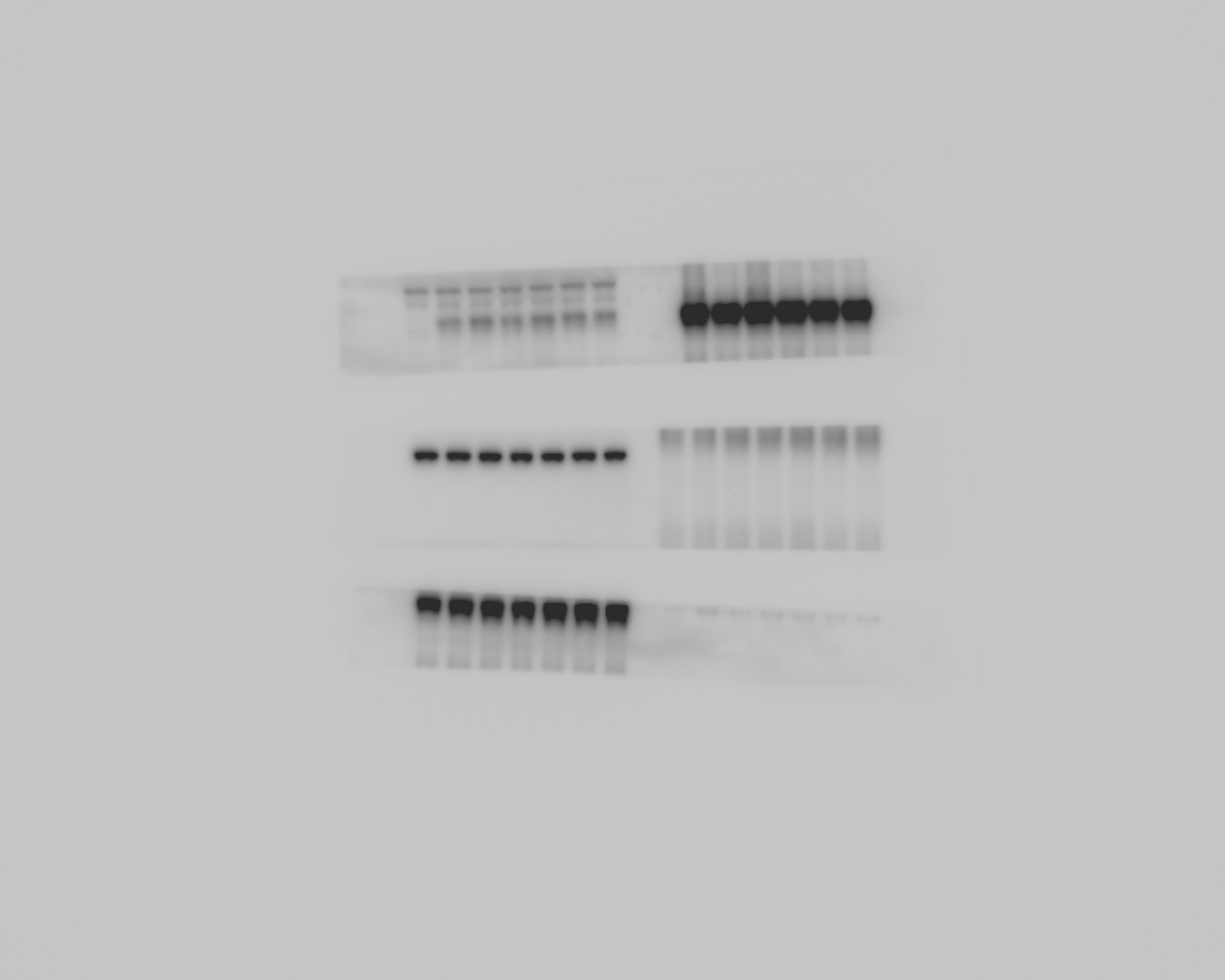

Supplement: Figure 1—source data 1. [file elife-106730-fig1-data1.zip › Figure 1ΓÇösource data 1/Figure 1D/Fig1D_Flag_actin_usp28_4(Chemiluminescence).tif]

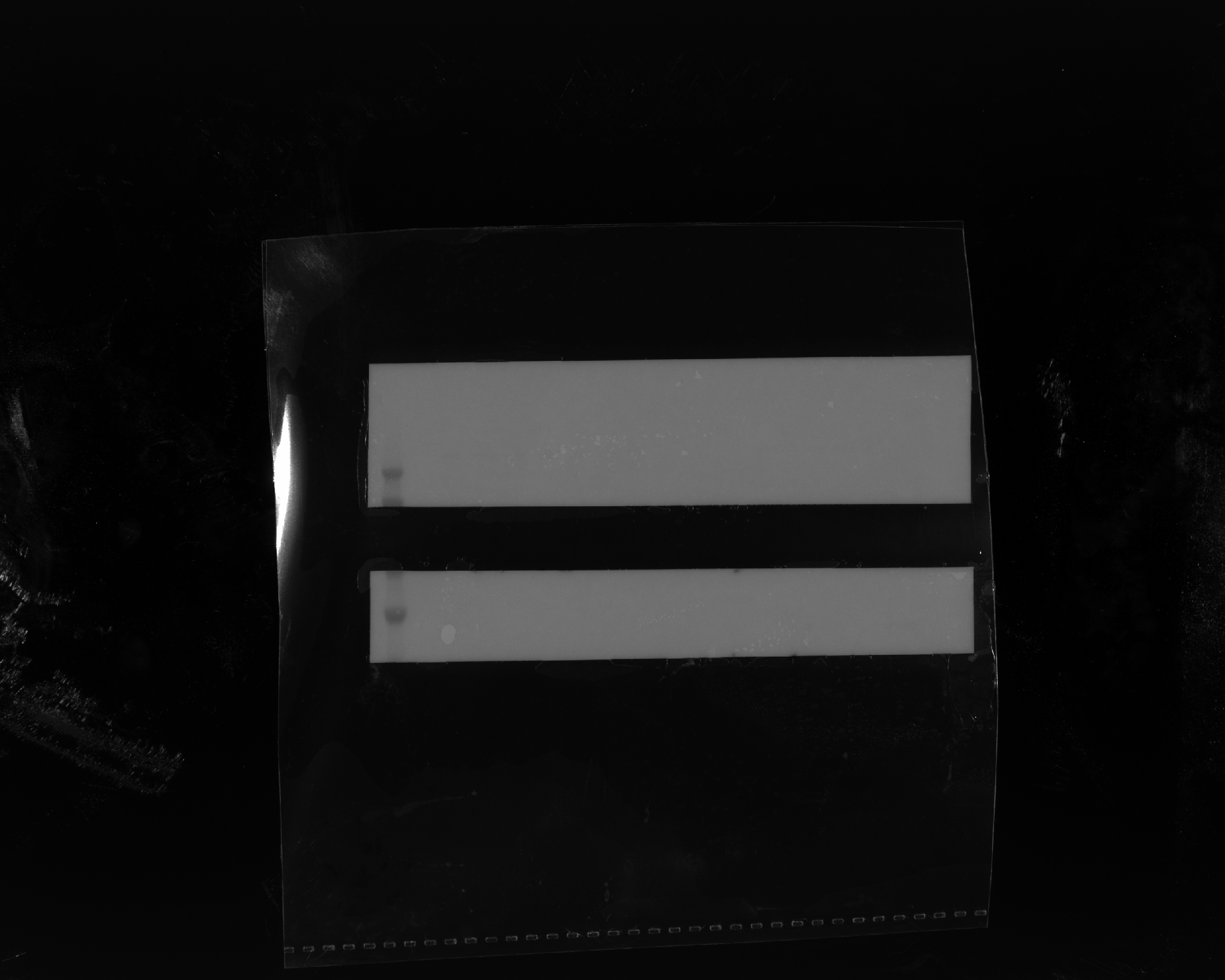

Supplement: Figure 1—source data 1. [file elife-106730-fig1-data1.zip › Figure 1ΓÇösource data 1/Figure 1D/Fig1D_53bp1_cul3_11(Colorimetric).tif]

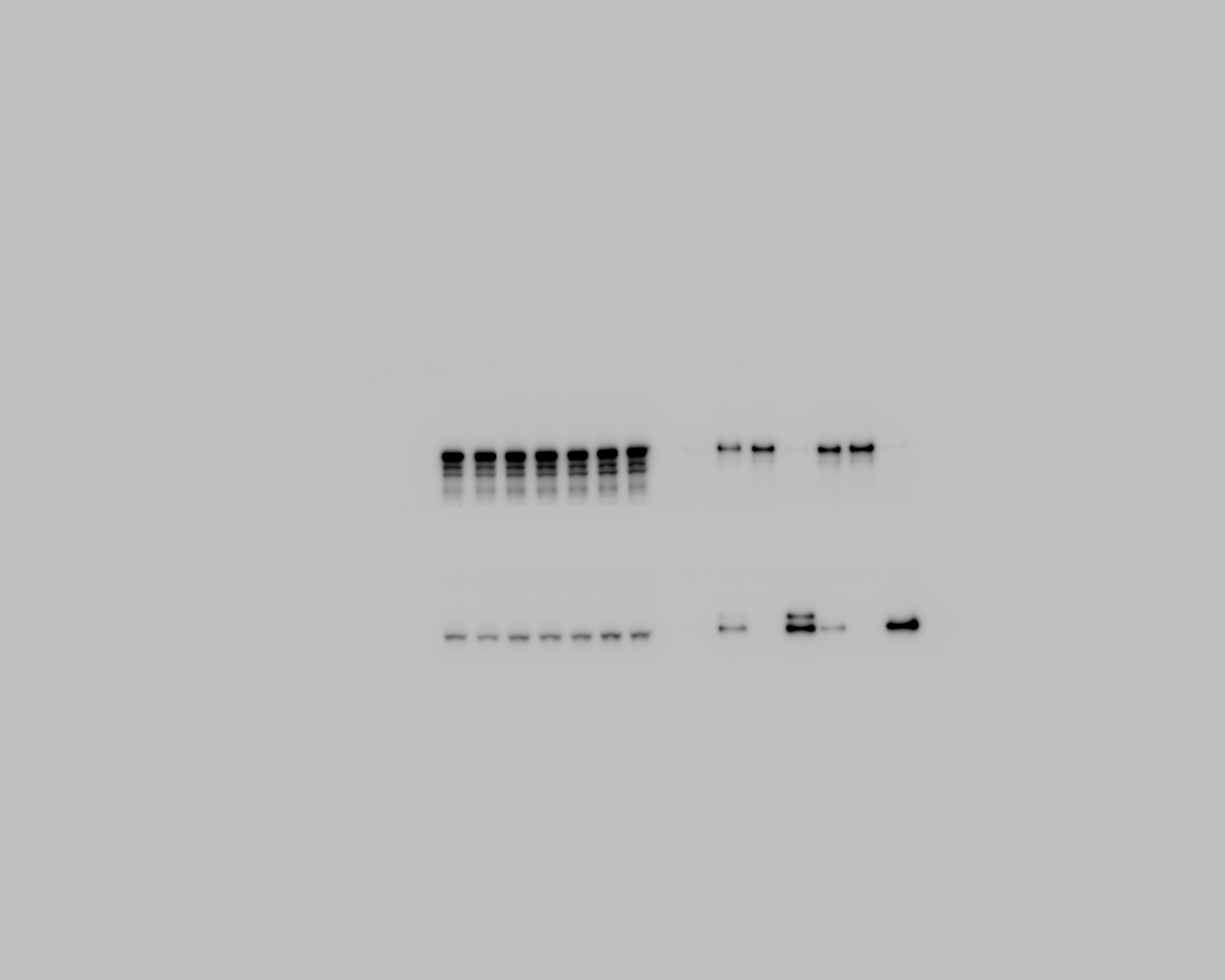

Supplement: Figure 1—source data 1. [file elife-106730-fig1-data1.zip › Figure 1ΓÇösource data 1/Figure 1D/Fig1D_53bp1_cul3_02(Chemiluminescence).tif]

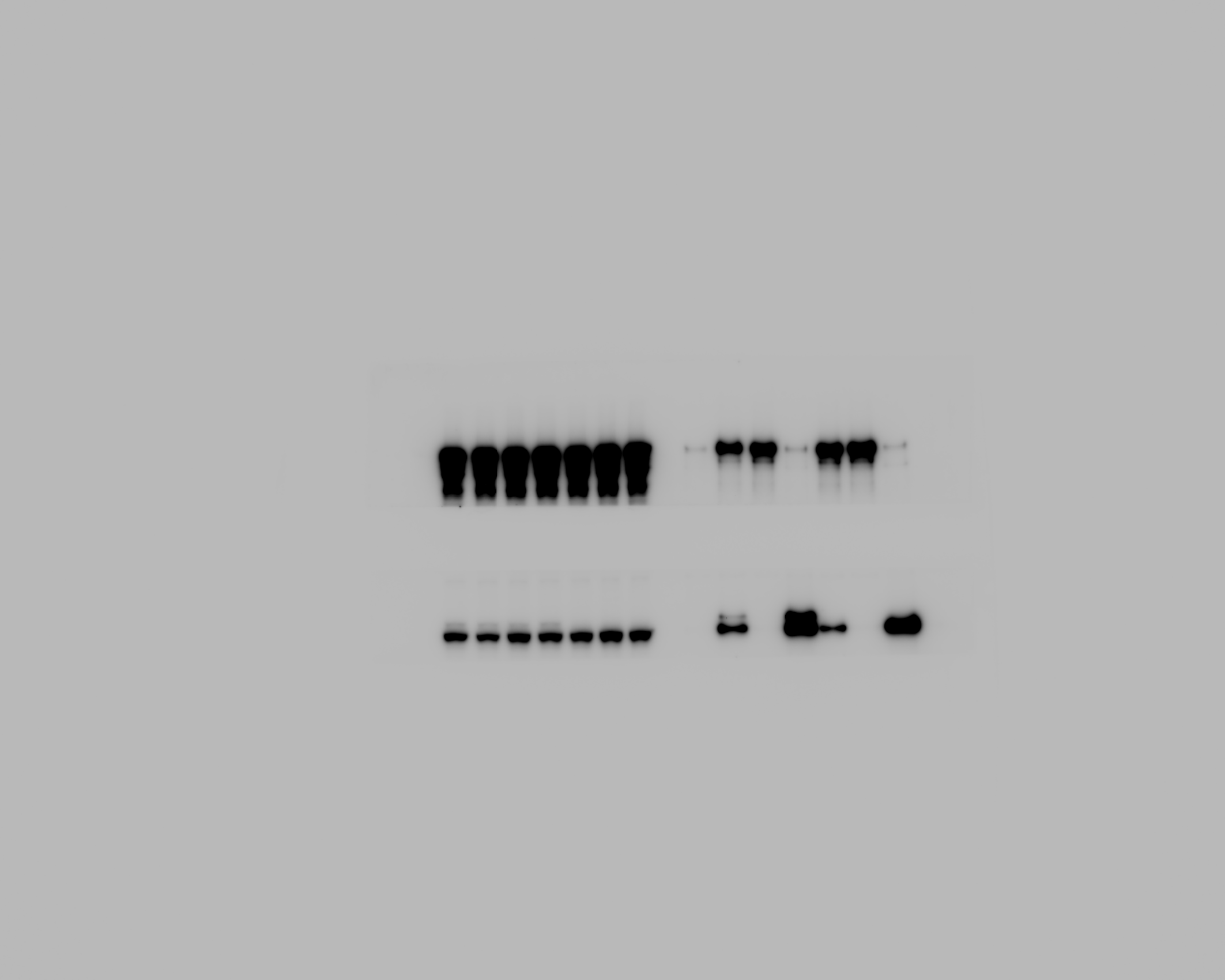

Supplement: Figure 1—source data 1. [file elife-106730-fig1-data1.zip › Figure 1ΓÇösource data 1/Figure 1D/Fig1D_53bp1_cul3_03(Chemiluminescence).tif]

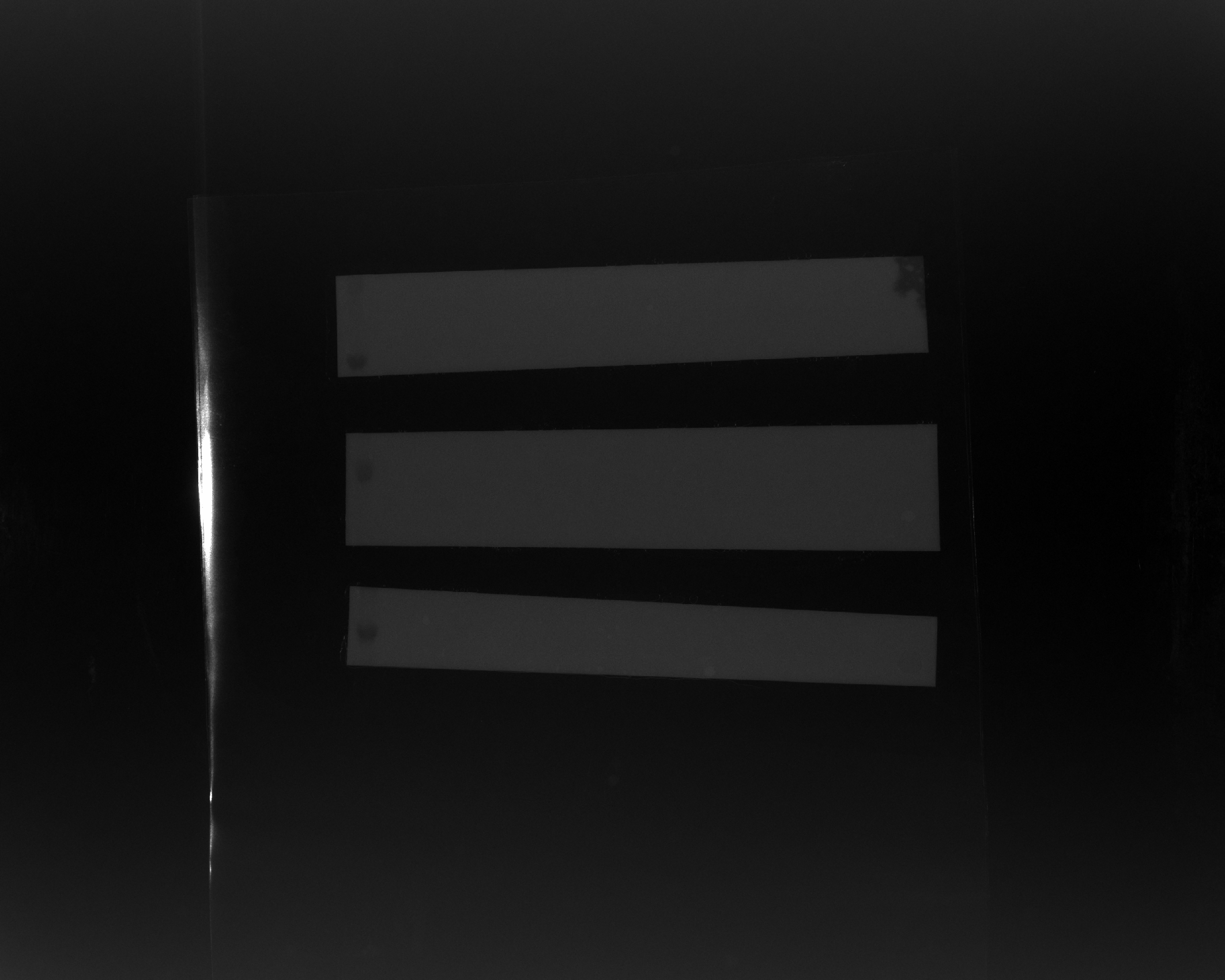

Supplement: Figure 1—source data 1. [file elife-106730-fig1-data1.zip › Figure 1ΓÇösource data 1/Figure 1D/Fig1D_Flag_actin_usp28_6(Colorimetric).tif]

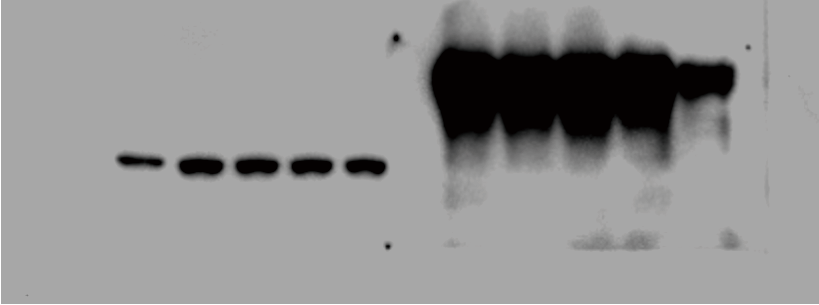

Supplement: Figure 1—source data 1. [file elife-106730-fig1-data1.zip › Figure 1ΓÇösource data 1/Figure 1C/actin.png]

Figure1E

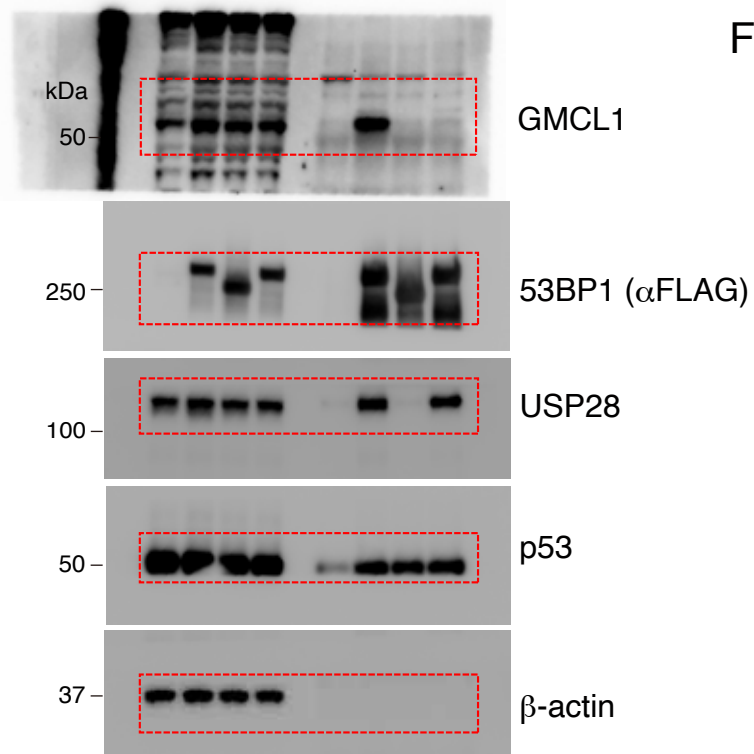

Figure1F

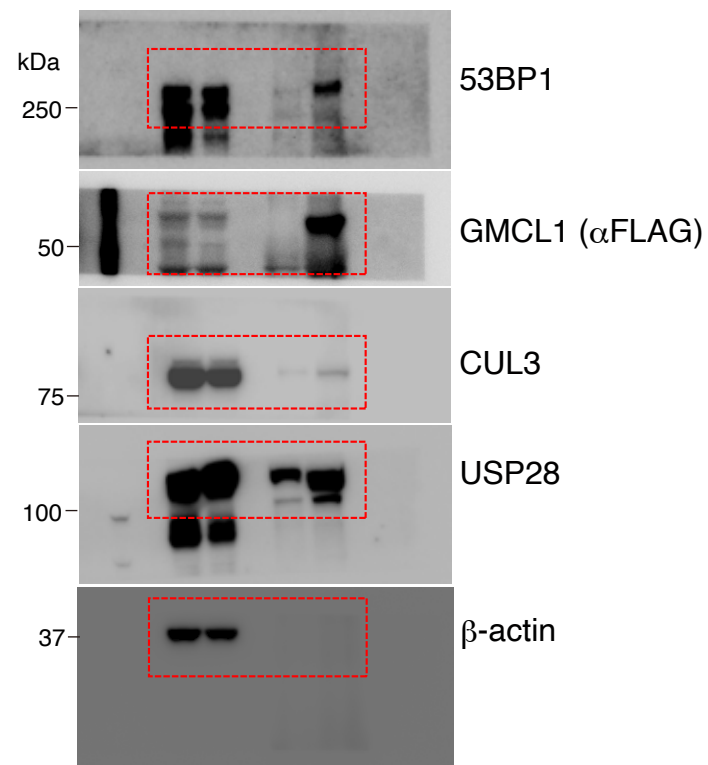

Supplement: Figure 1—source data 2. [file elife-106730-fig1-data2.zip › Figure 1ΓÇösource data 2/Figure1-2_Raw uncropped supporting Western blot files.pdf]

Figure1C

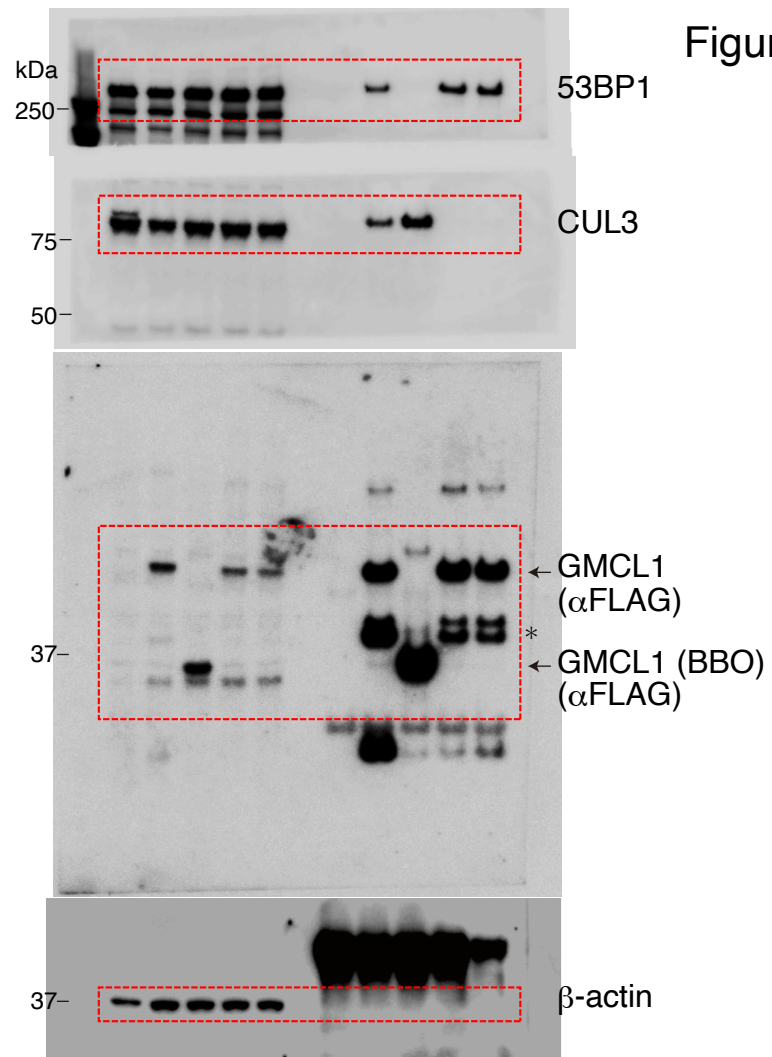

Figure1D

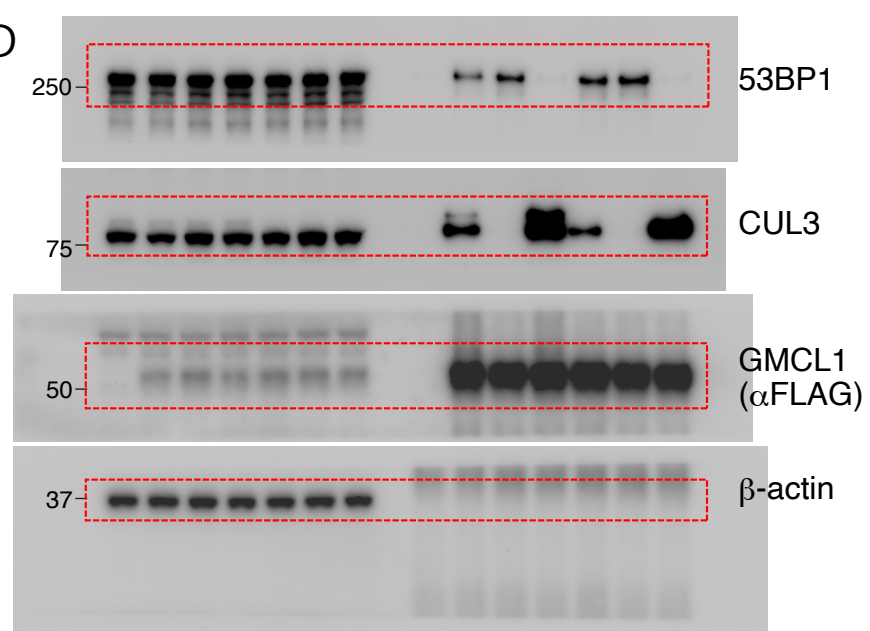

Supplement: Figure 1—source data 2. [file elife-106730-fig1-data2.zip › Figure 1ΓÇösource data 2/Figure1-1_Raw uncropped supporting Western blot files.pdf]

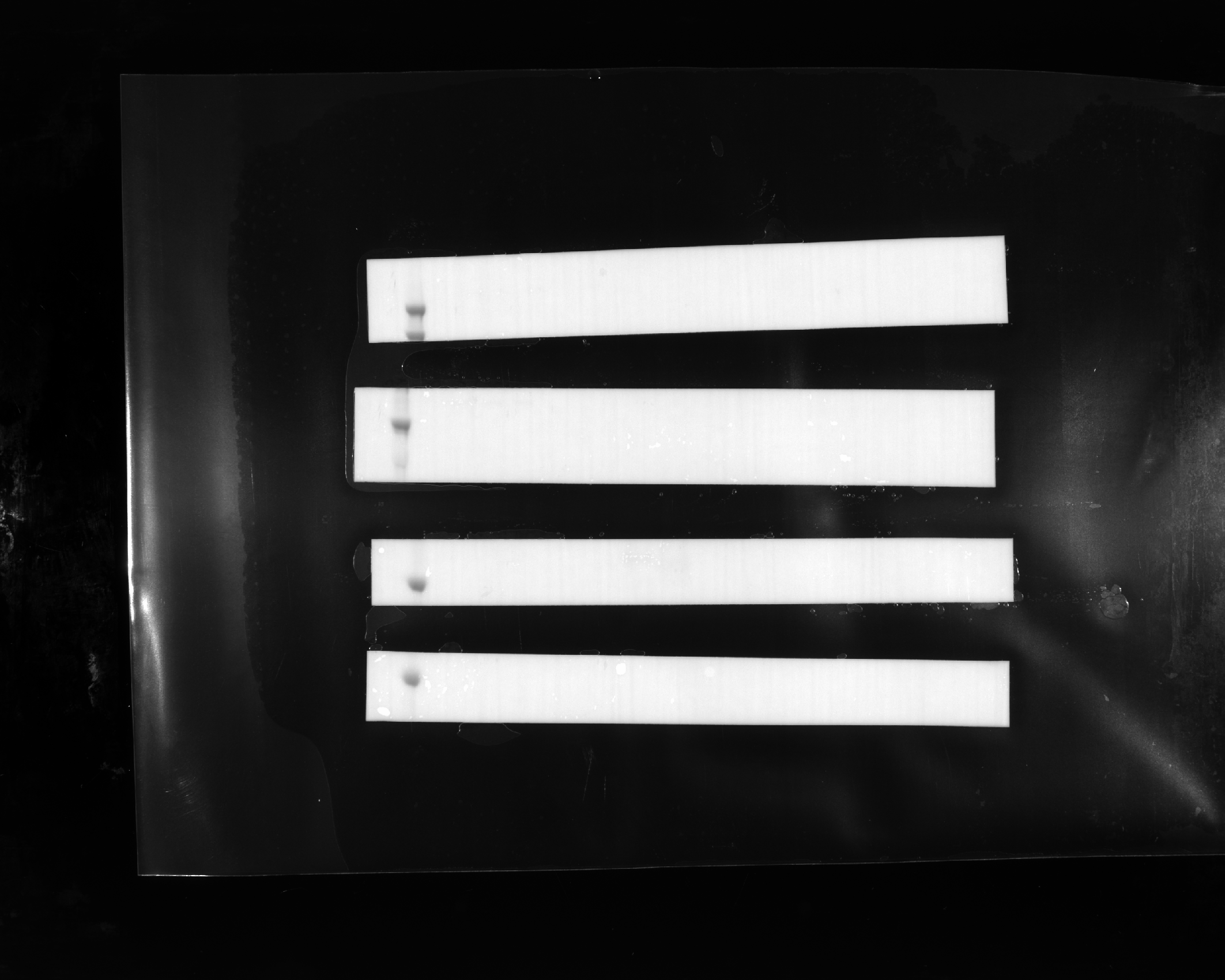

Supplement: Figure 1—figure supplement 1—source data 1. [file elife-106730-fig1-figsupp1-data1.zip › Figure 1ΓÇöfigure supplement 1ΓÇösource data 1/Figure 1ΓÇöfigure supplement 1F/GMCL1&2-IP_53bp1_usp28_gmcl1_actin_11(Colorimetric).tif]

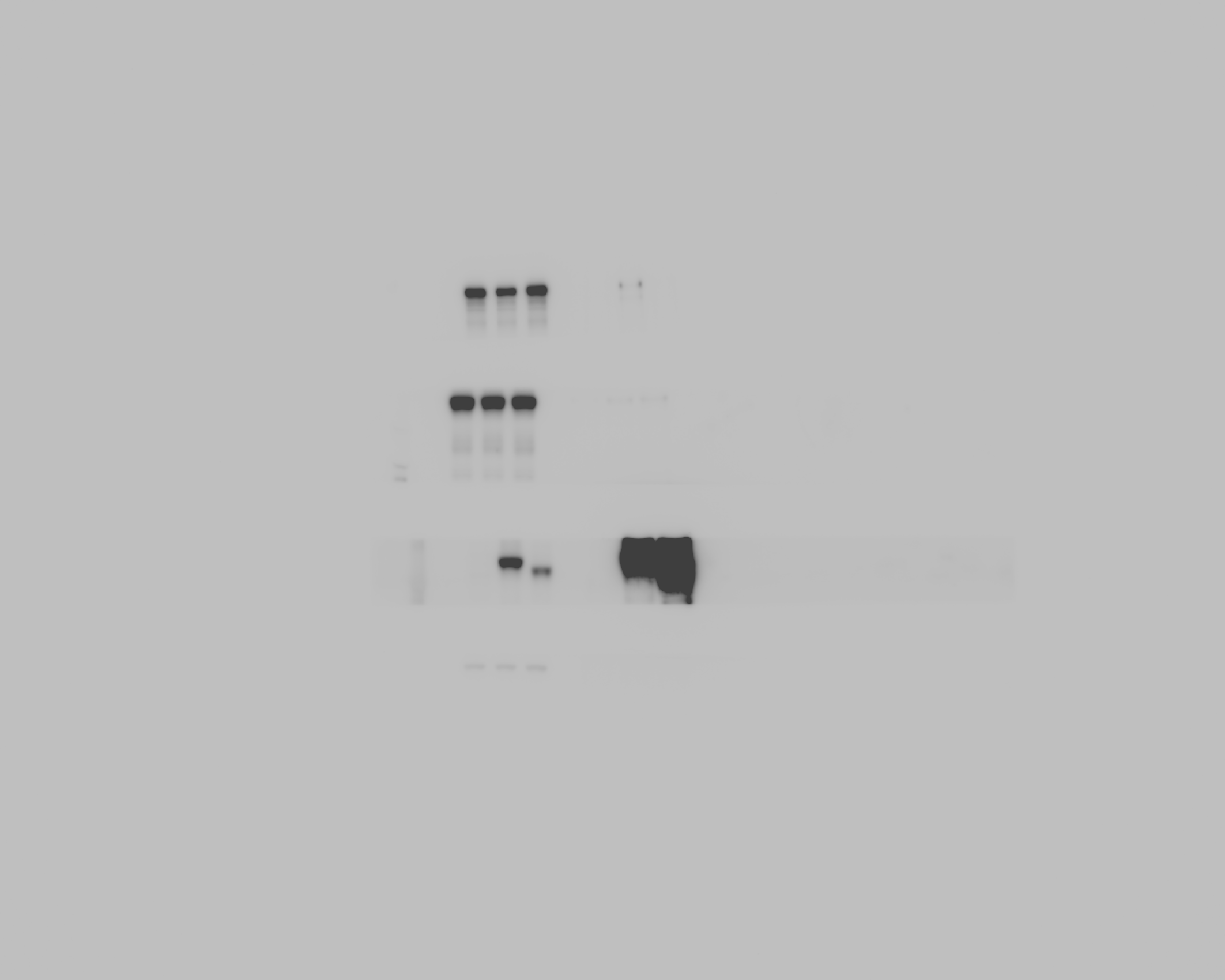

Supplement: Figure 1—figure supplement 1—source data 1. [file elife-106730-fig1-figsupp1-data1.zip › Figure 1ΓÇöfigure supplement 1ΓÇösource data 1/Figure 1ΓÇöfigure supplement 1F/GMCL1&2-IP_53bp1_usp28_gmcl1_actin_04(Chemiluminescence_Background).tif]

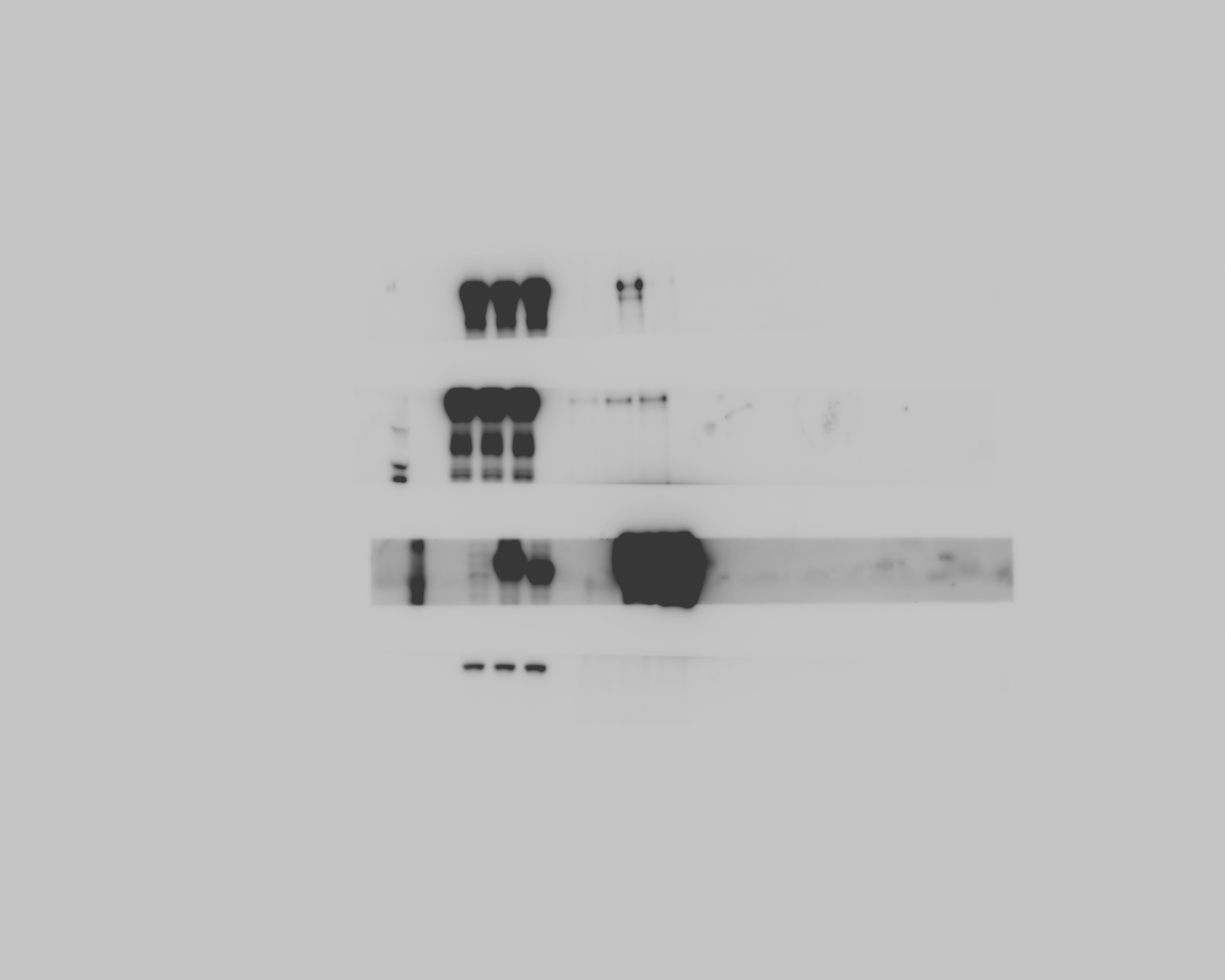

Supplement: Figure 1—figure supplement 1—source data 1. [file elife-106730-fig1-figsupp1-data1.zip › Figure 1ΓÇöfigure supplement 1ΓÇösource data 1/Figure 1ΓÇöfigure supplement 1F/GMCL1&2-IP_53bp1_usp28_gmcl1_actin_09(Chemiluminescence_Background).tif]

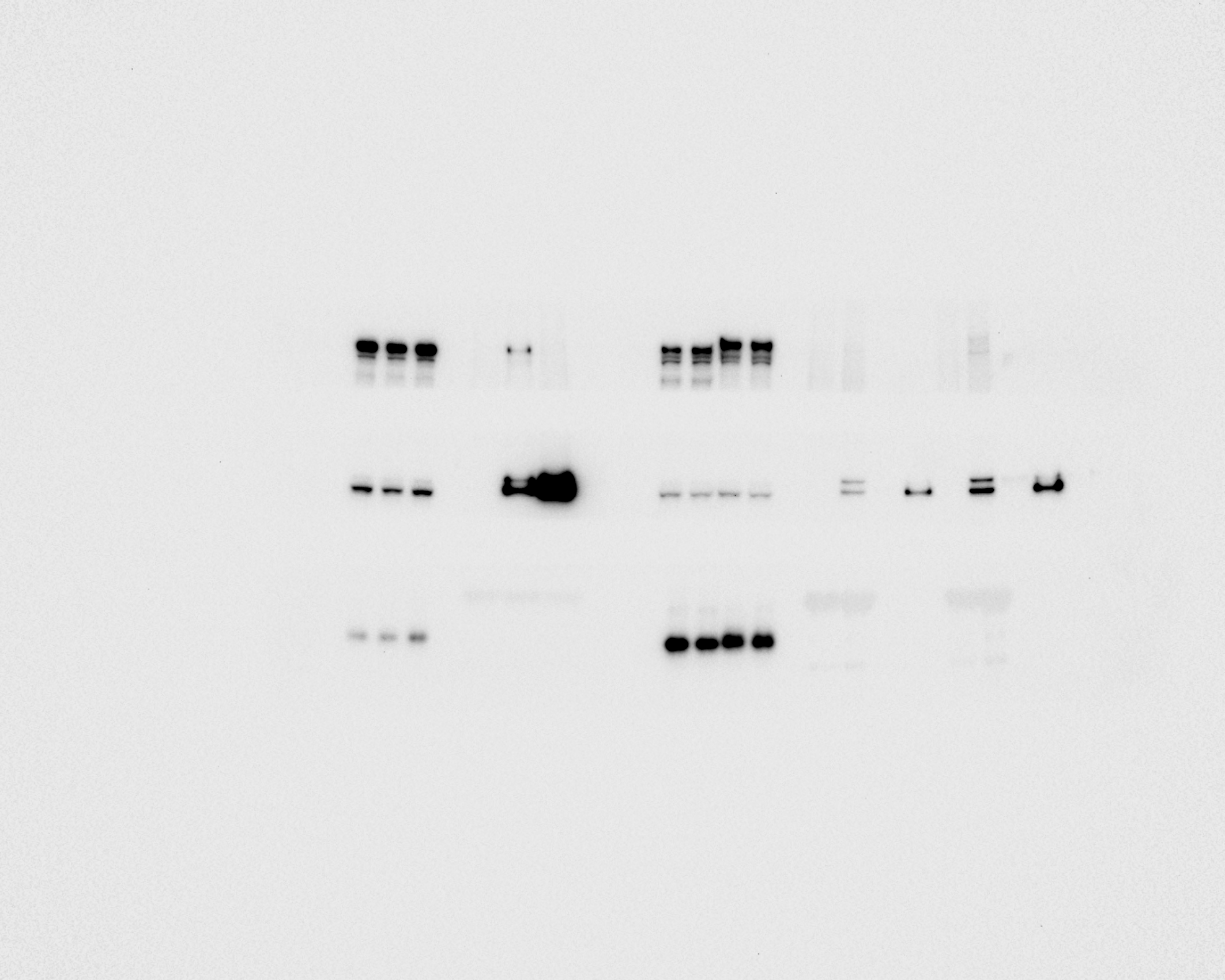

Supplement: Figure 1—figure supplement 1—source data 1. [file elife-106730-fig1-figsupp1-data1.zip › Figure 1ΓÇöfigure supplement 1ΓÇösource data 1/Figure 1ΓÇöfigure supplement 1F/122024-GMCL1&2OE_53bp1_cul3_p21__Flag_06(Chemiluminescence).tif]

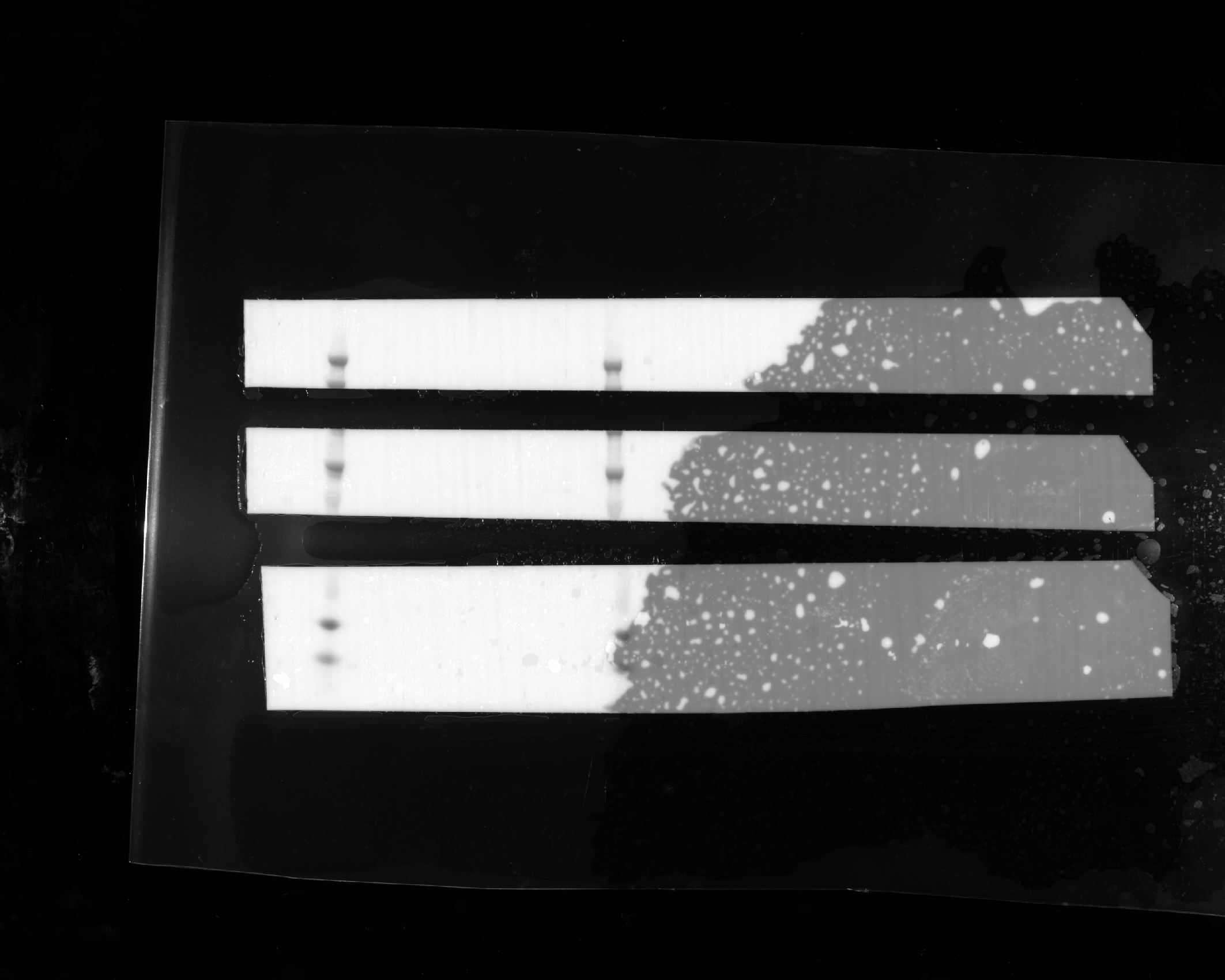

Supplement: Figure 1—figure supplement 1—source data 1. [file elife-106730-fig1-figsupp1-data1.zip › Figure 1ΓÇöfigure supplement 1ΓÇösource data 1/Figure 1ΓÇöfigure supplement 1F/122024-GMCL1&2OE_53bp1_cul3_p21__Flag_12(Colorimetric).tif]

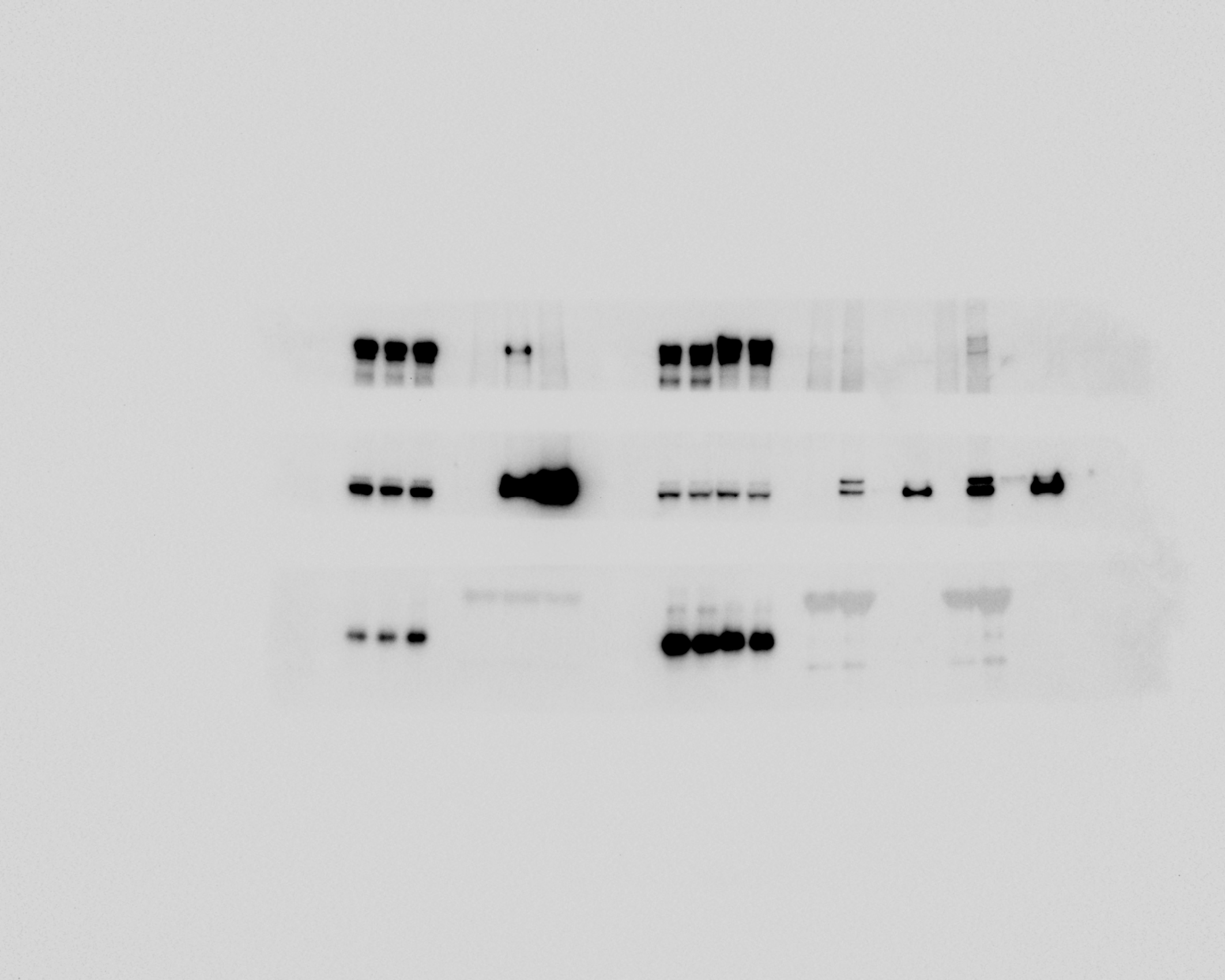

Supplement: Figure 1—figure supplement 1—source data 1. [file elife-106730-fig1-figsupp1-data1.zip › Figure 1ΓÇöfigure supplement 1ΓÇösource data 1/Figure 1ΓÇöfigure supplement 1F/122024-GMCL1&2OE_53bp1_cul3_p21__Flag_08(Chemiluminescence).tif]

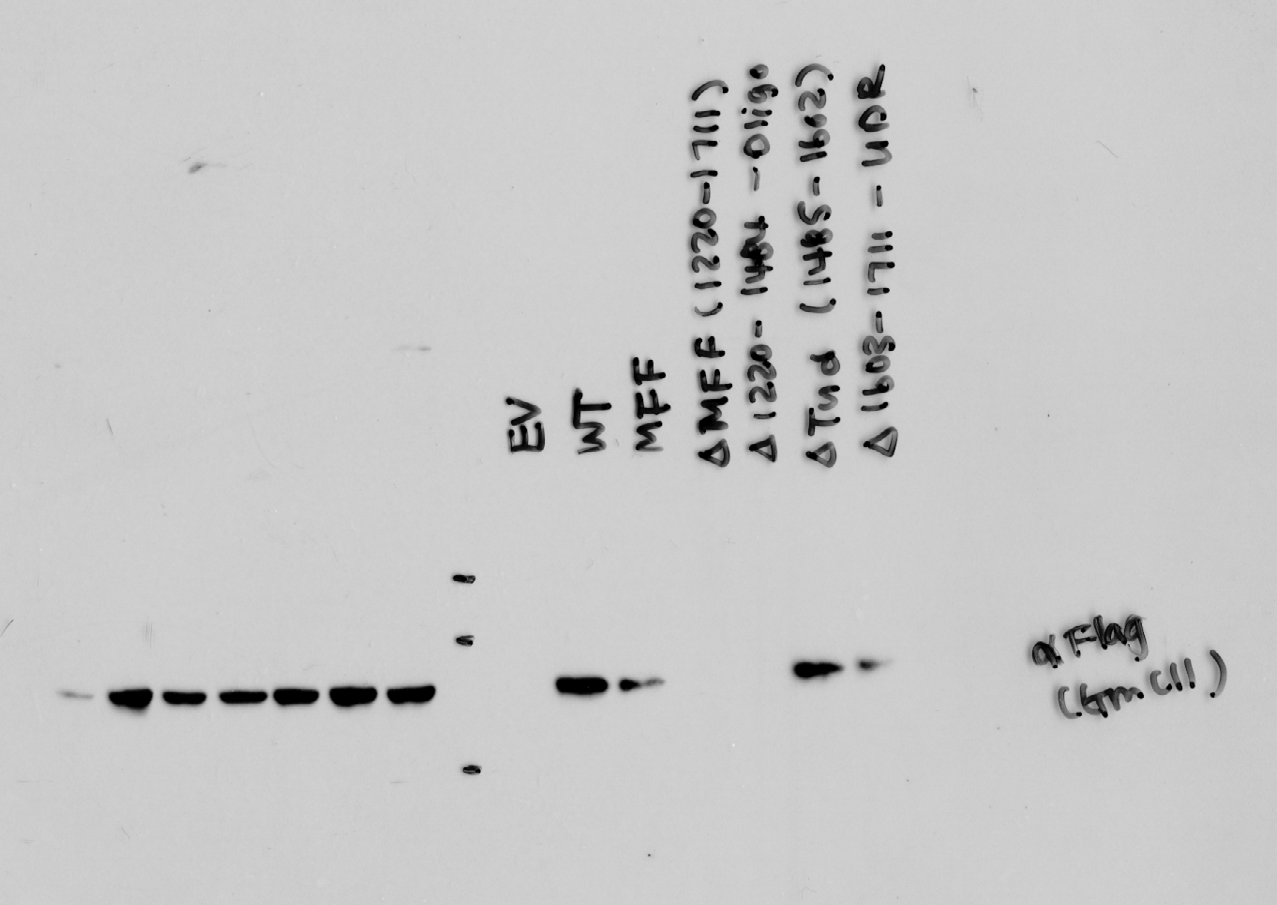

Supplement: Figure 1—figure supplement 1—source data 1. [file elife-106730-fig1-figsupp1-data1.zip › Figure 1ΓÇöfigure supplement 1ΓÇösource data 1/Figure 1ΓÇöfigure supplement 1D/flag001.tif]

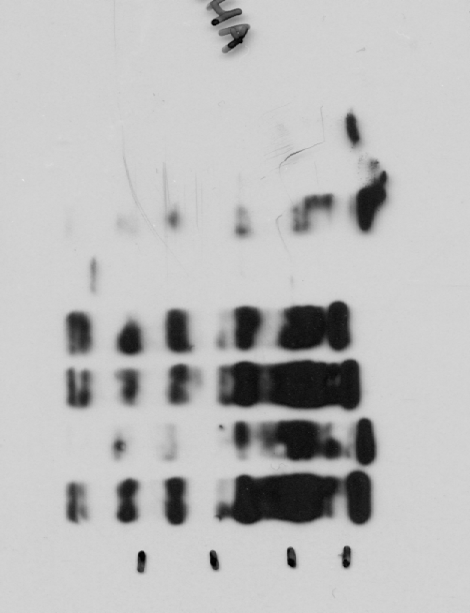

Supplement: Figure 1—figure supplement 1—source data 1. [file elife-106730-fig1-figsupp1-data1.zip › Figure 1ΓÇöfigure supplement 1ΓÇösource data 1/Figure 1ΓÇöfigure supplement 1D/HA006.tif]

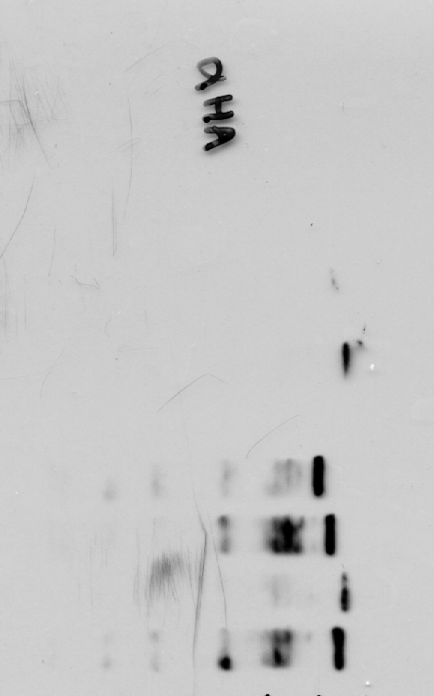

Supplement: Figure 1—figure supplement 1—source data 1. [file elife-106730-fig1-figsupp1-data1.zip › Figure 1ΓÇöfigure supplement 1ΓÇösource data 1/Figure 1ΓÇöfigure supplement 1D/HA005.tif]

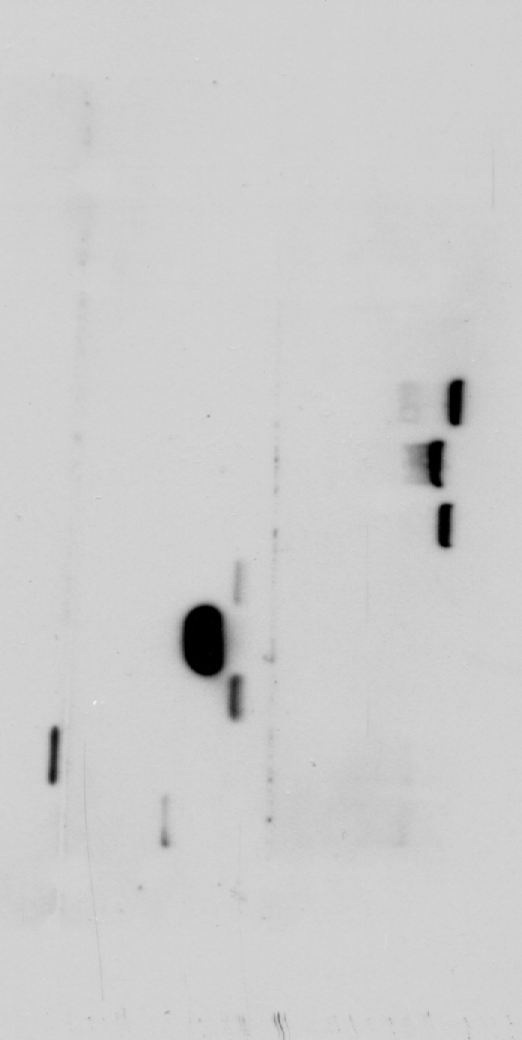

Supplement: Figure 1—figure supplement 1—source data 1. [file elife-106730-fig1-figsupp1-data1.zip › Figure 1ΓÇöfigure supplement 1ΓÇösource data 1/Figure 1ΓÇöfigure supplement 1C/HA002.tif]

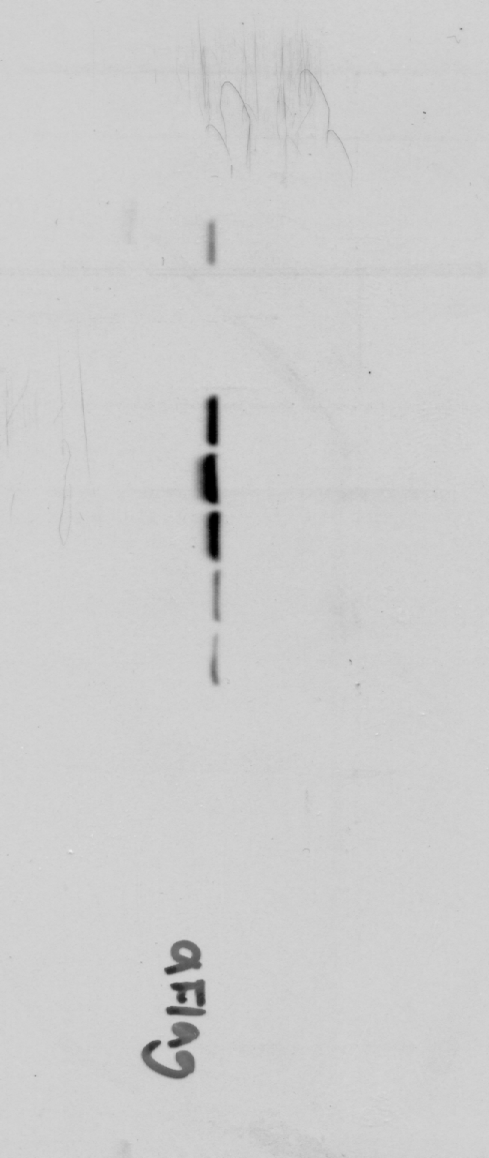

Supplement: Figure 1—figure supplement 1—source data 1. [file elife-106730-fig1-figsupp1-data1.zip › Figure 1ΓÇöfigure supplement 1ΓÇösource data 1/Figure 1ΓÇöfigure supplement 1C/Flag001.tif]

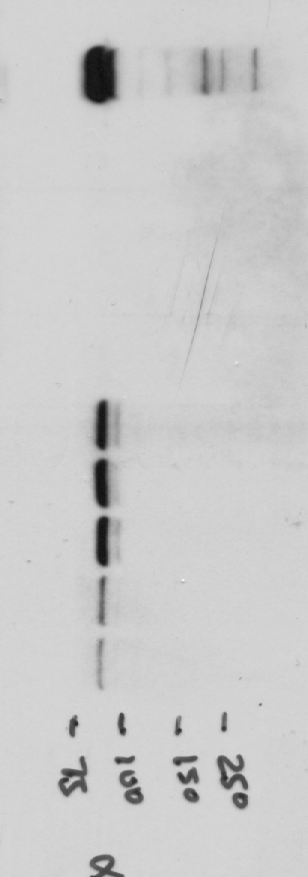

Supplement: Figure 1—figure supplement 1—source data 1. [file elife-106730-fig1-figsupp1-data1.zip › Figure 1ΓÇöfigure supplement 1ΓÇösource data 1/Figure 1ΓÇöfigure supplement 1C/cul3005.tif]

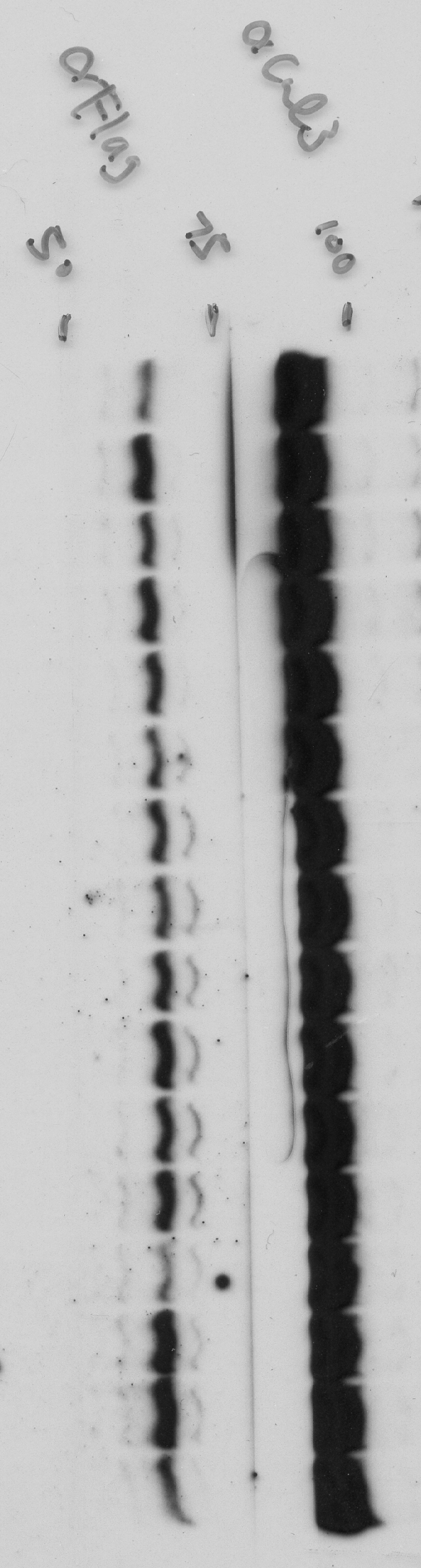

Supplement: Figure 1—figure supplement 1—source data 1. [file elife-106730-fig1-figsupp1-data1.zip › Figure 1ΓÇöfigure supplement 1ΓÇösource data 1/Figure 1ΓÇöfigure supplement 1E/INPUT-FLAG006.tif]

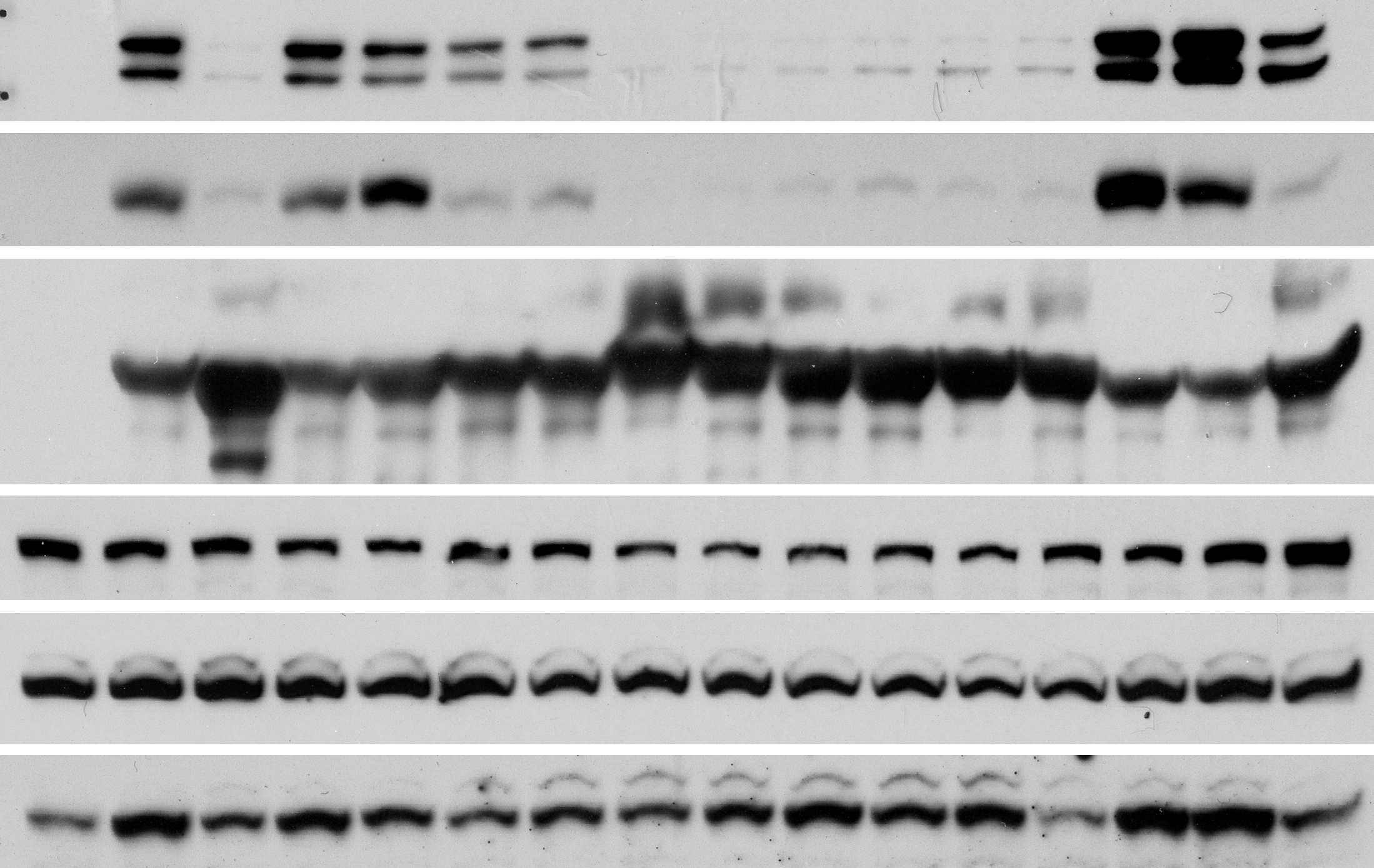

Supplement: Figure 1—figure supplement 1—source data 1. [file elife-106730-fig1-figsupp1-data1.zip › Figure 1ΓÇöfigure supplement 1ΓÇösource data 1/Figure 1ΓÇöfigure supplement 1E/Untitled-1.tif]

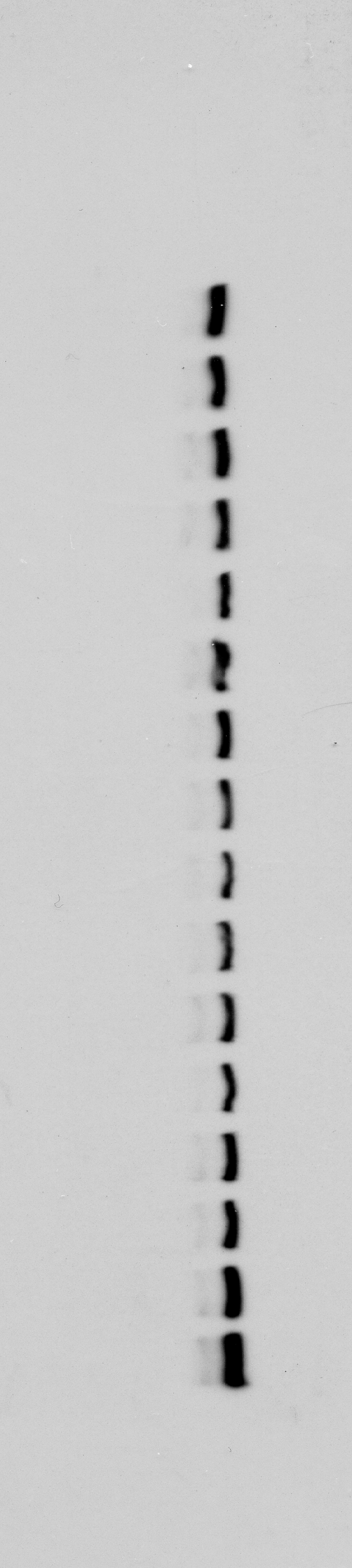

Supplement: Figure 1—figure supplement 1—source data 1. [file elife-106730-fig1-figsupp1-data1.zip › Figure 1ΓÇöfigure supplement 1ΓÇösource data 1/Figure 1ΓÇöfigure supplement 1E/INPUT-53BP1005.tif]

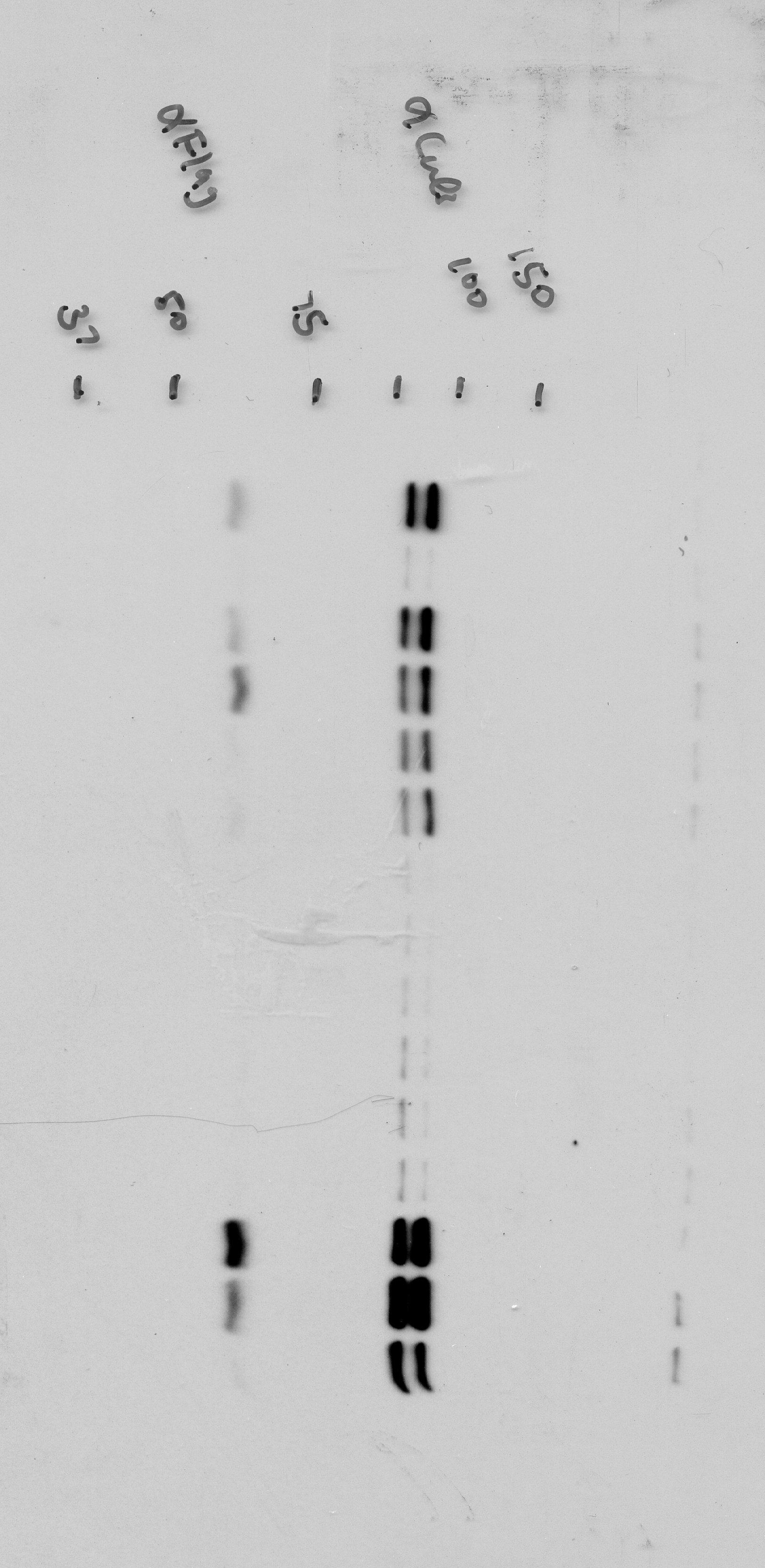

Supplement: Figure 1—figure supplement 1—source data 1. [file elife-106730-fig1-figsupp1-data1.zip › Figure 1ΓÇöfigure supplement 1ΓÇösource data 1/Figure 1ΓÇöfigure supplement 1E/IP001.tif]

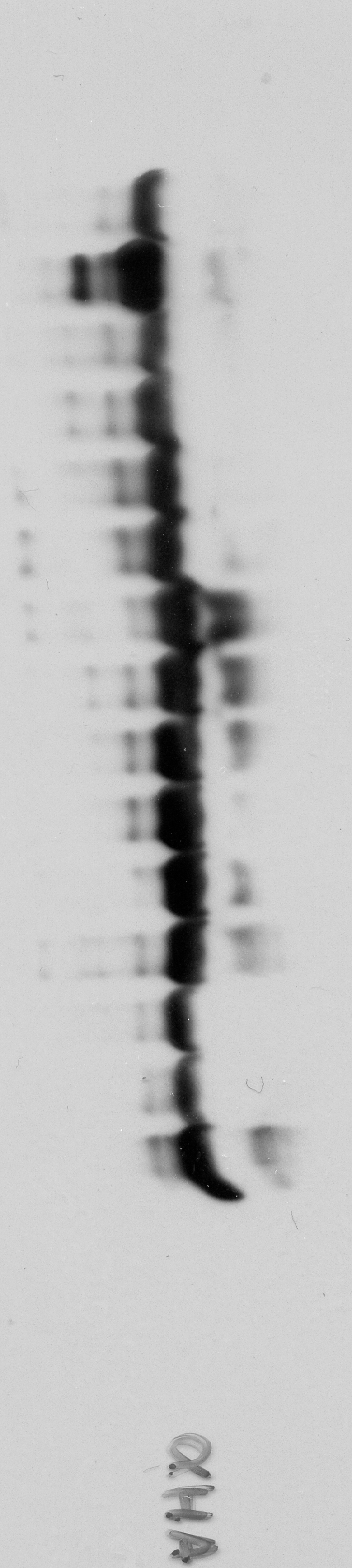

Supplement: Figure 1—figure supplement 1—source data 1. [file elife-106730-fig1-figsupp1-data1.zip › Figure 1ΓÇöfigure supplement 1ΓÇösource data 1/Figure 1ΓÇöfigure supplement 1E/IP003.tif]

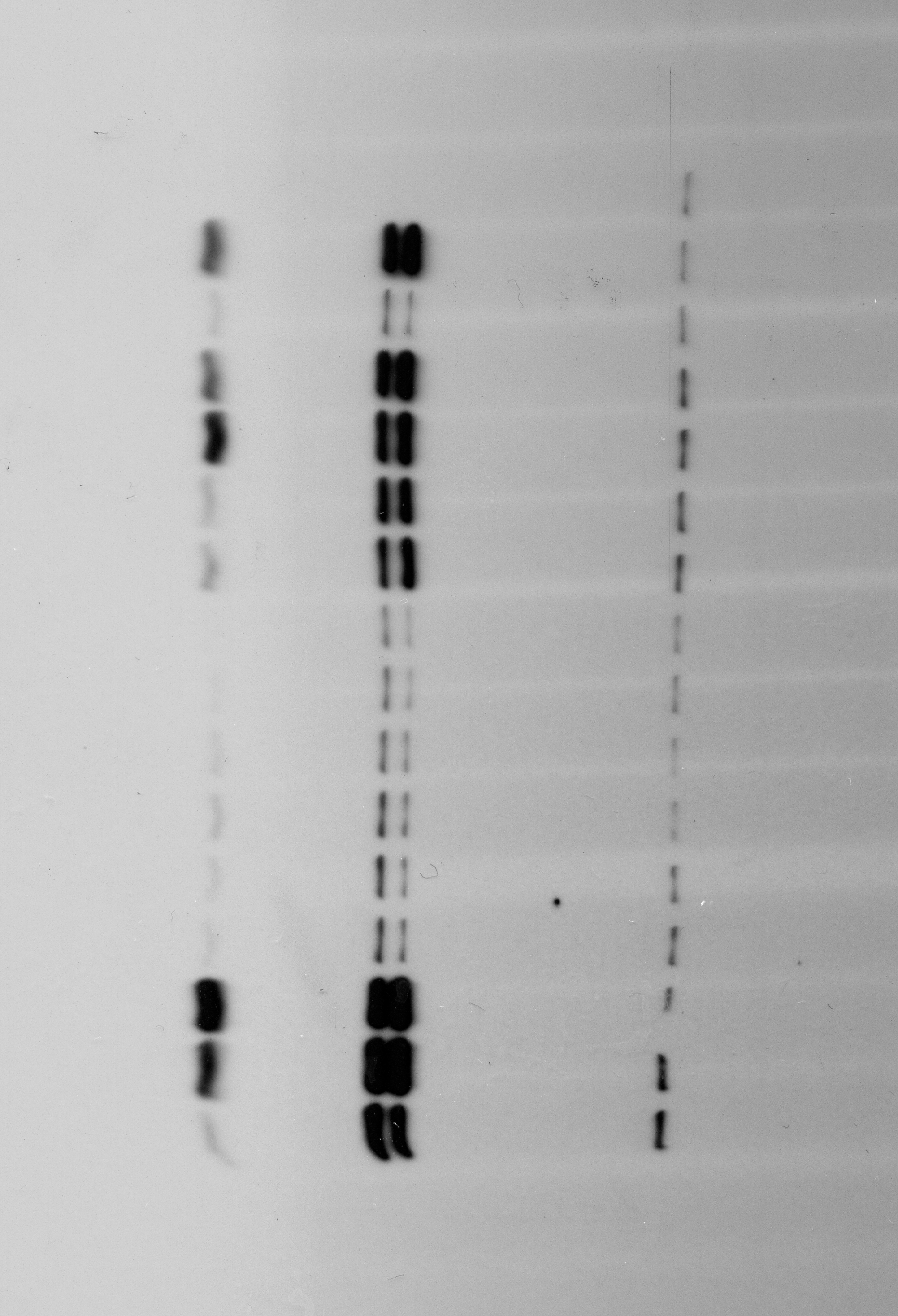

Supplement: Figure 1—figure supplement 1—source data 1. [file elife-106730-fig1-figsupp1-data1.zip › Figure 1ΓÇöfigure supplement 1ΓÇösource data 1/Figure 1ΓÇöfigure supplement 1E/IP002.tif]

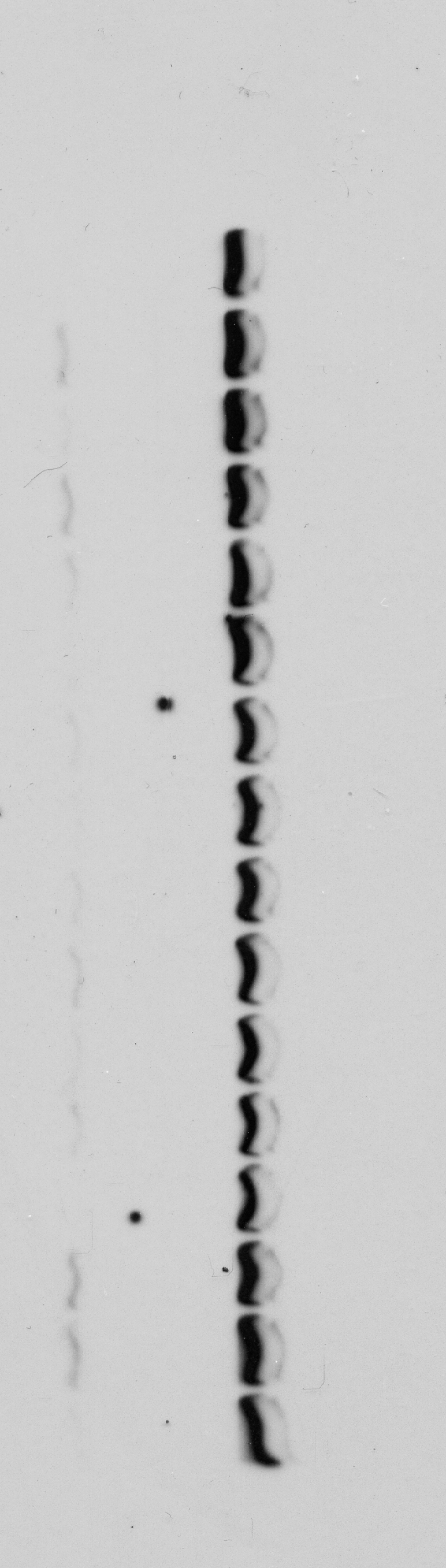

Supplement: Figure 1—figure supplement 1—source data 1. [file elife-106730-fig1-figsupp1-data1.zip › Figure 1ΓÇöfigure supplement 1ΓÇösource data 1/Figure 1ΓÇöfigure supplement 1E/INPUT004.tif]

Supplemental  
Figure1E

CUL3

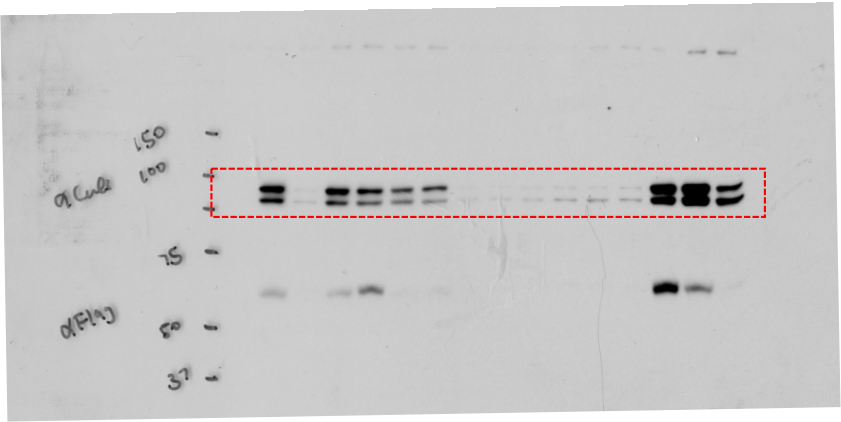

GMCL1 ( $\alpha$ FLAG)

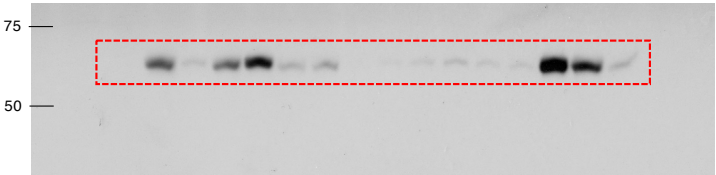

53BP1 MFF ( $\alpha$ HA)

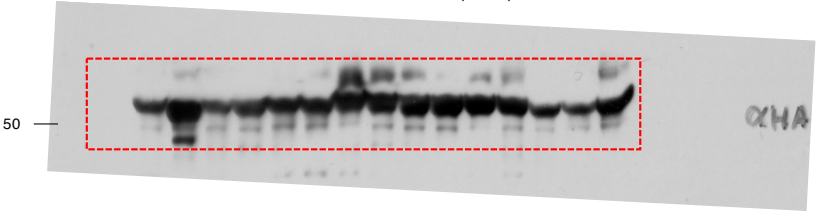

53BP1

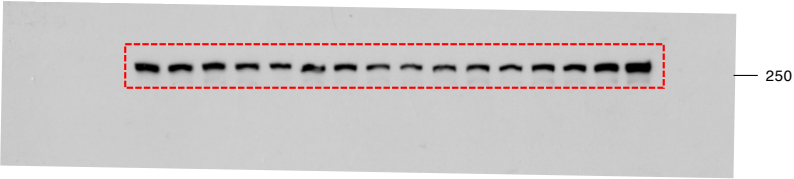

CUL3

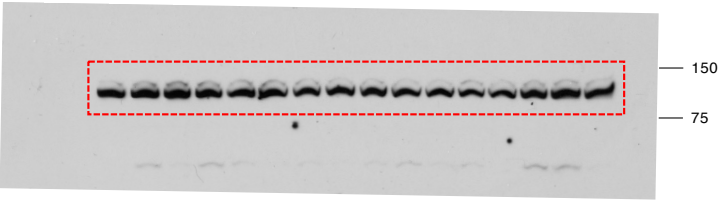

GMCL1 ( $\alpha$ FLAG)

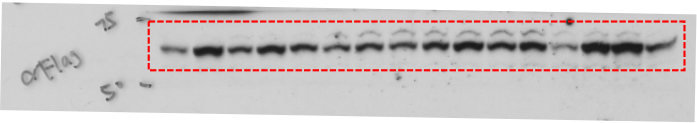

Supplement: Figure 1—figure supplement 1—source data 2. [file elife-106730-fig1-figsupp1-data2.zip › Figure 1ΓÇöfigure supplement 1ΓÇösource data 2/SupFigure1-2_Raw uncropped supporting Western blot files.pdf]

Supplemental  
Figure1F

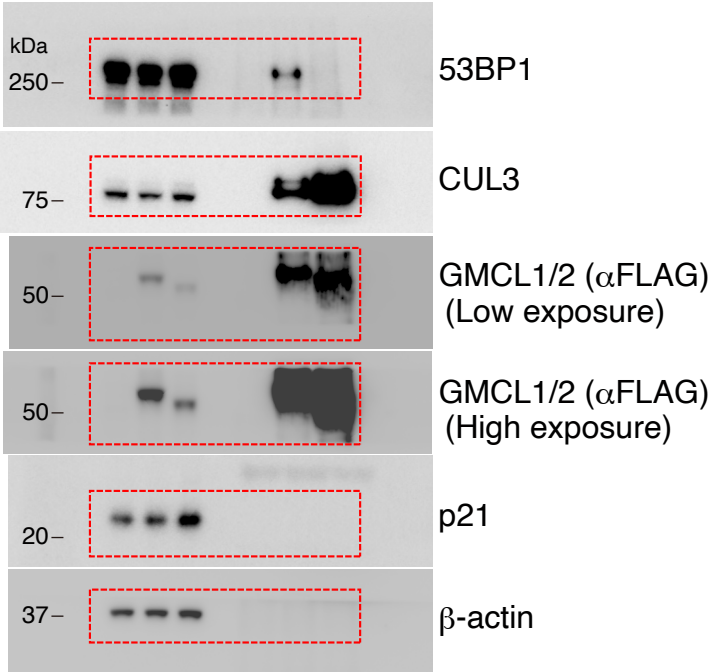

Supplement: Figure 1—figure supplement 1—source data 2. [file elife-106730-fig1-figsupp1-data2.zip › Figure 1ΓÇöfigure supplement 1ΓÇösource data 2/SupFigure1-3_Raw uncropped supporting Western blot files.pdf]

Supplemental  
Figure1C

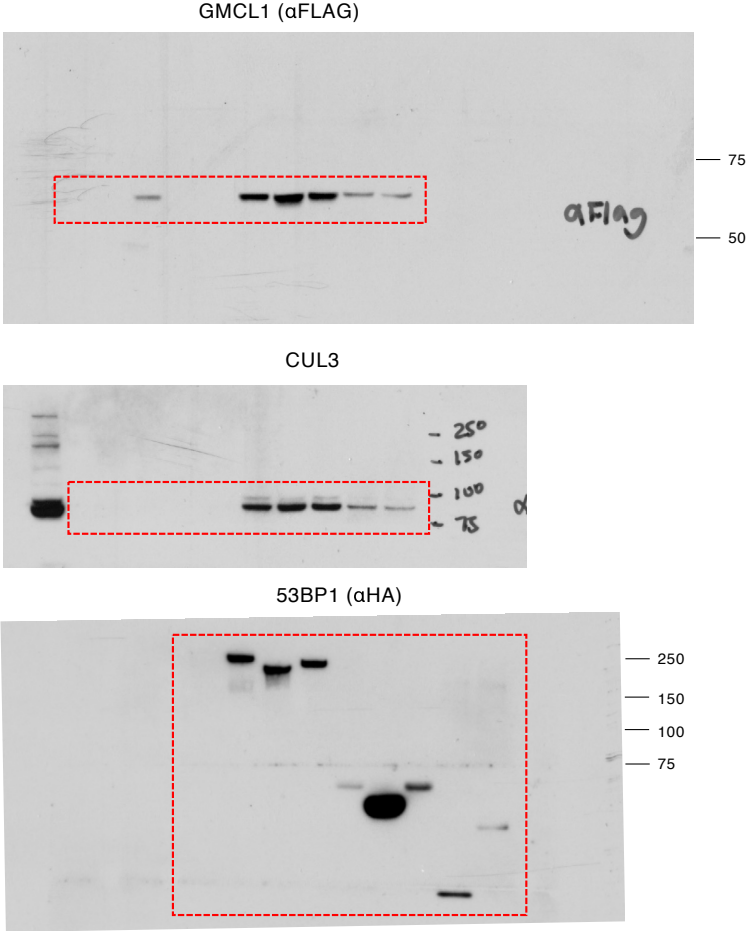

Supplemental  
Figure1D

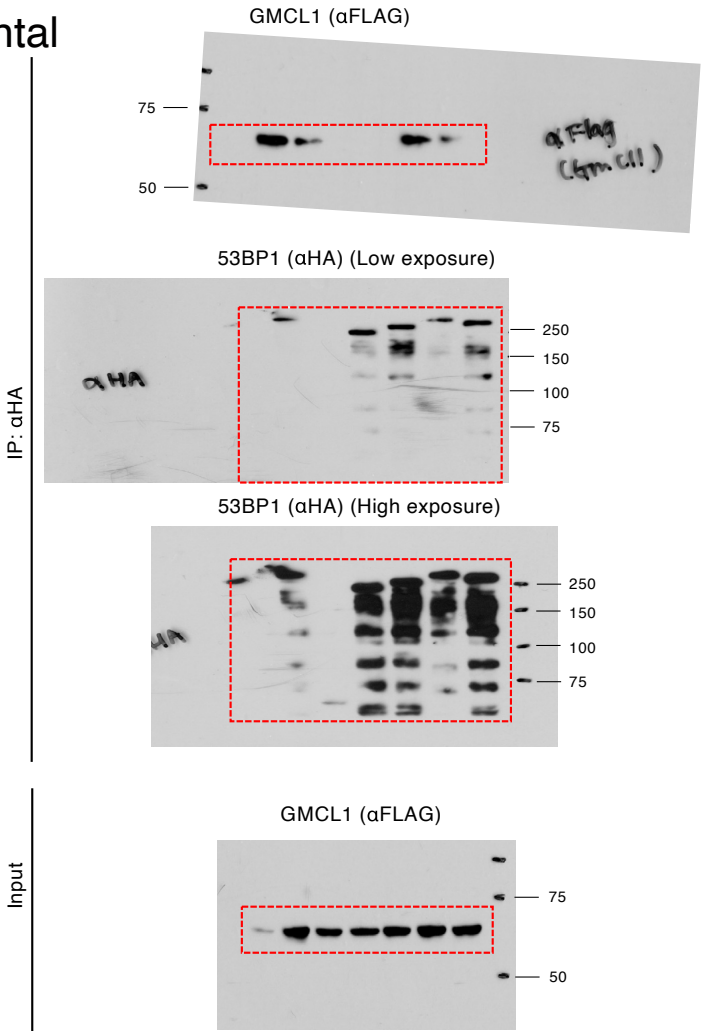

Supplement: Figure 1—figure supplement 1—source data 2. [file elife-106730-fig1-figsupp1-data2.zip › Figure 1ΓÇöfigure supplement 1ΓÇösource data 2/SupFigure1-1_Raw uncropped supporting Western blot files.pdf]

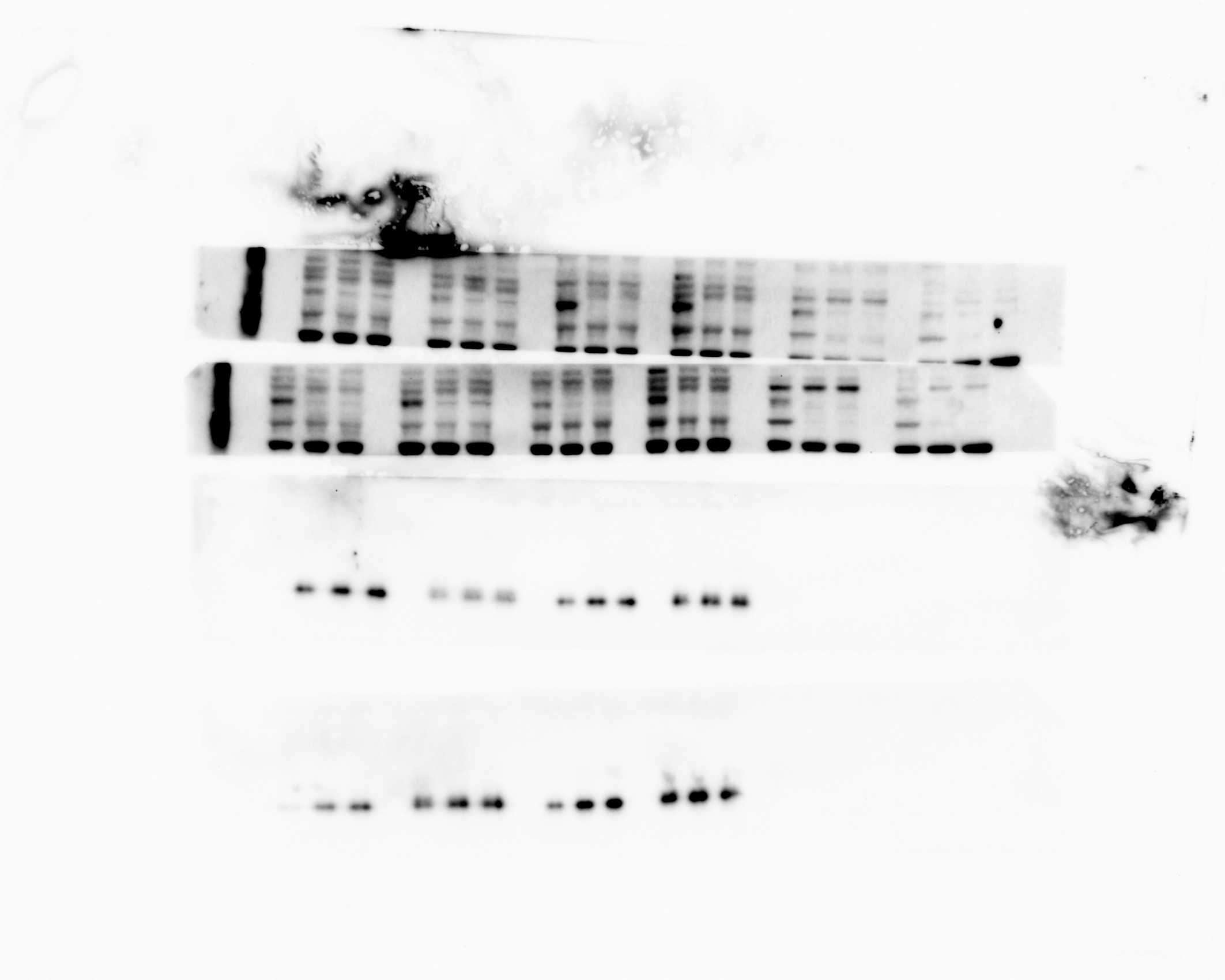

Supplement: Figure 2—source data 1. [file elife-106730-fig2-data1.zip › Figure 2ΓÇösource data 1/Figure 2A/011125-Fig2A_gmcl1_gmcl1_p21_p21_10(Chemiluminescence).tif]

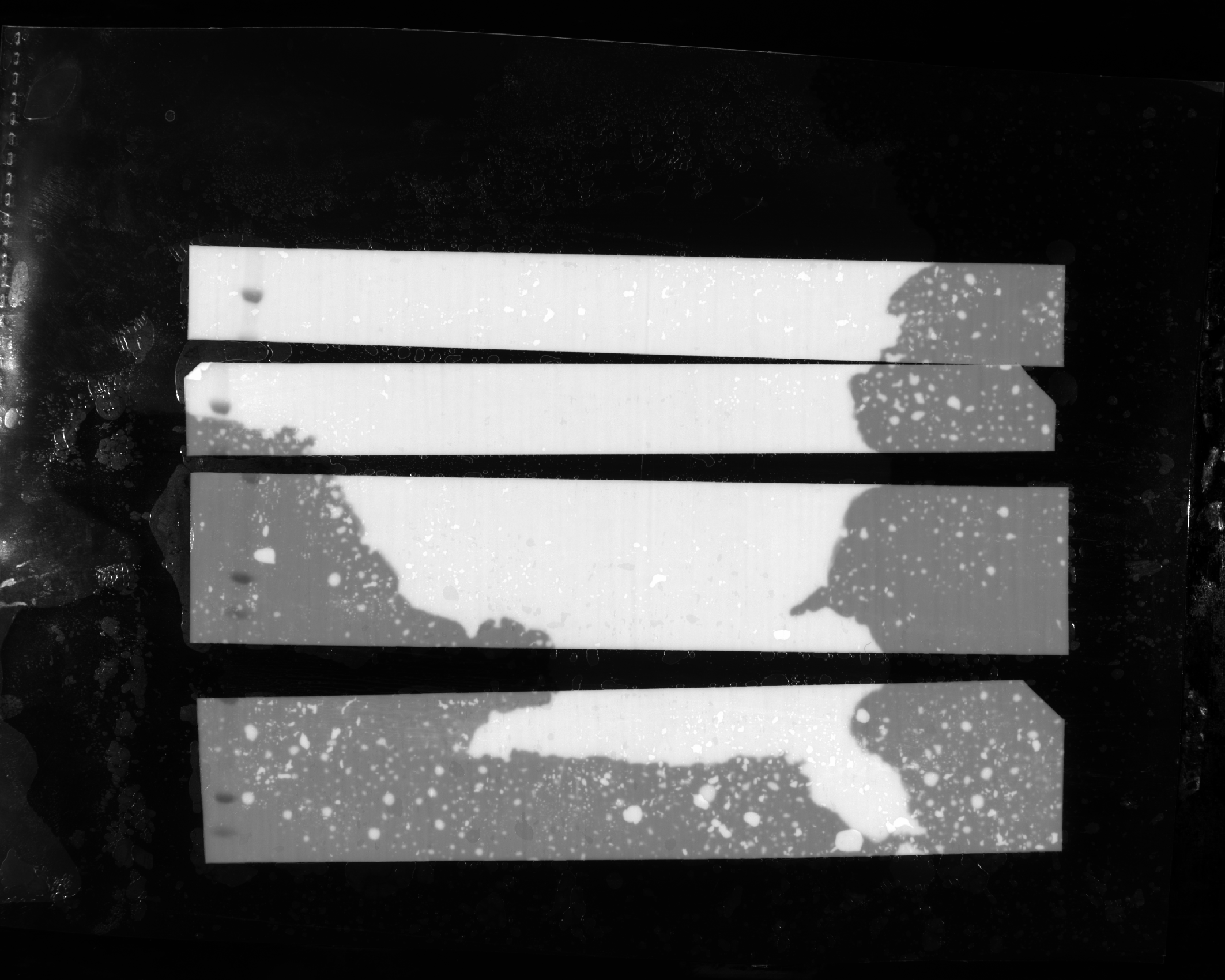

Supplement: Figure 2—source data 1. [file elife-106730-fig2-data1.zip › Figure 2ΓÇösource data 1/Figure 2A/011125-Fig2A_gmcl1_gmcl1_p21_p21_11(Colorimetric).tif]

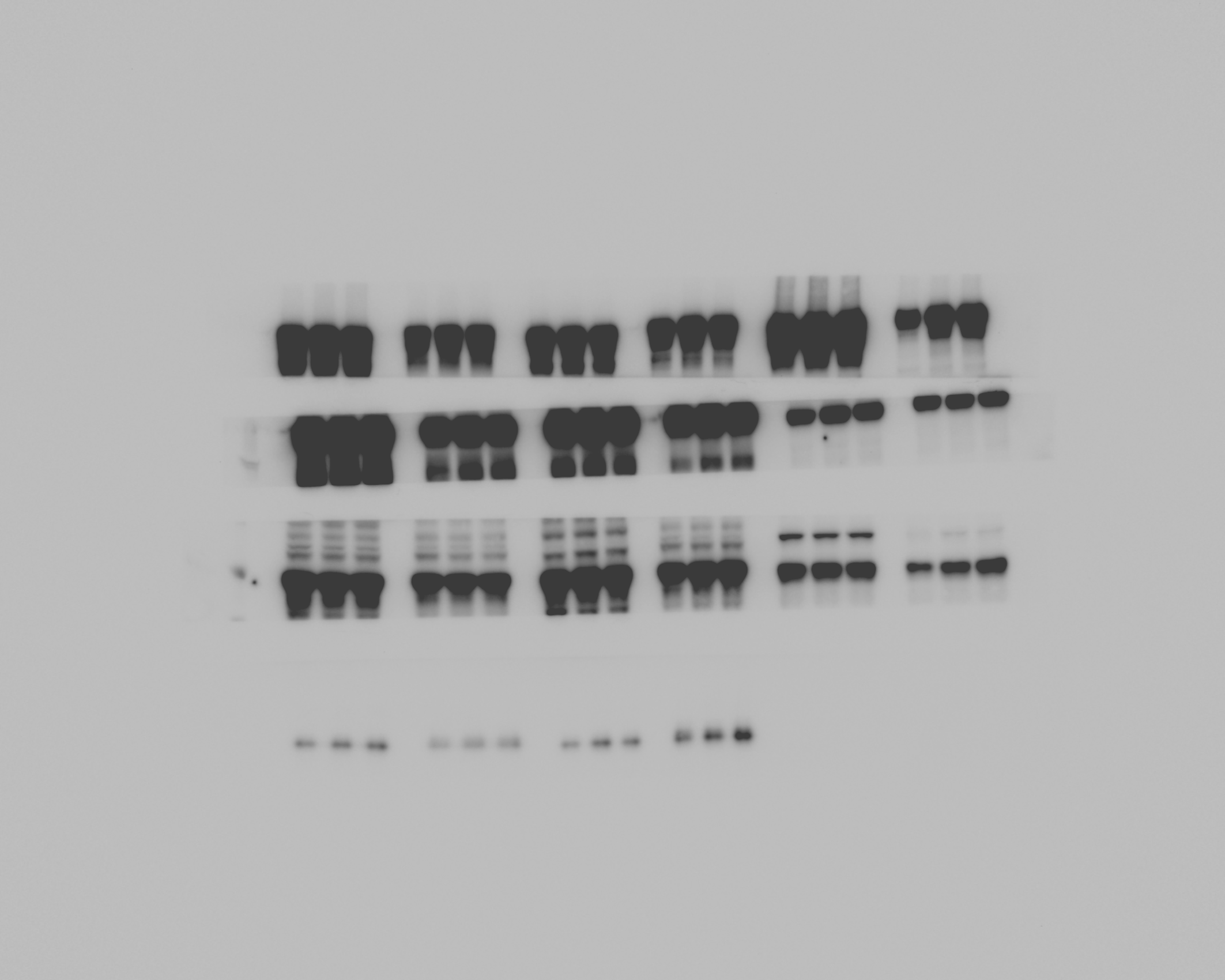

Supplement: Figure 2—source data 1. [file elife-106730-fig2-data1.zip › Figure 2ΓÇösource data 1/Figure 2A/011525-Fig2A_53bp1_usp28_p53_p21_5(Chemiluminescence_Background).tif]

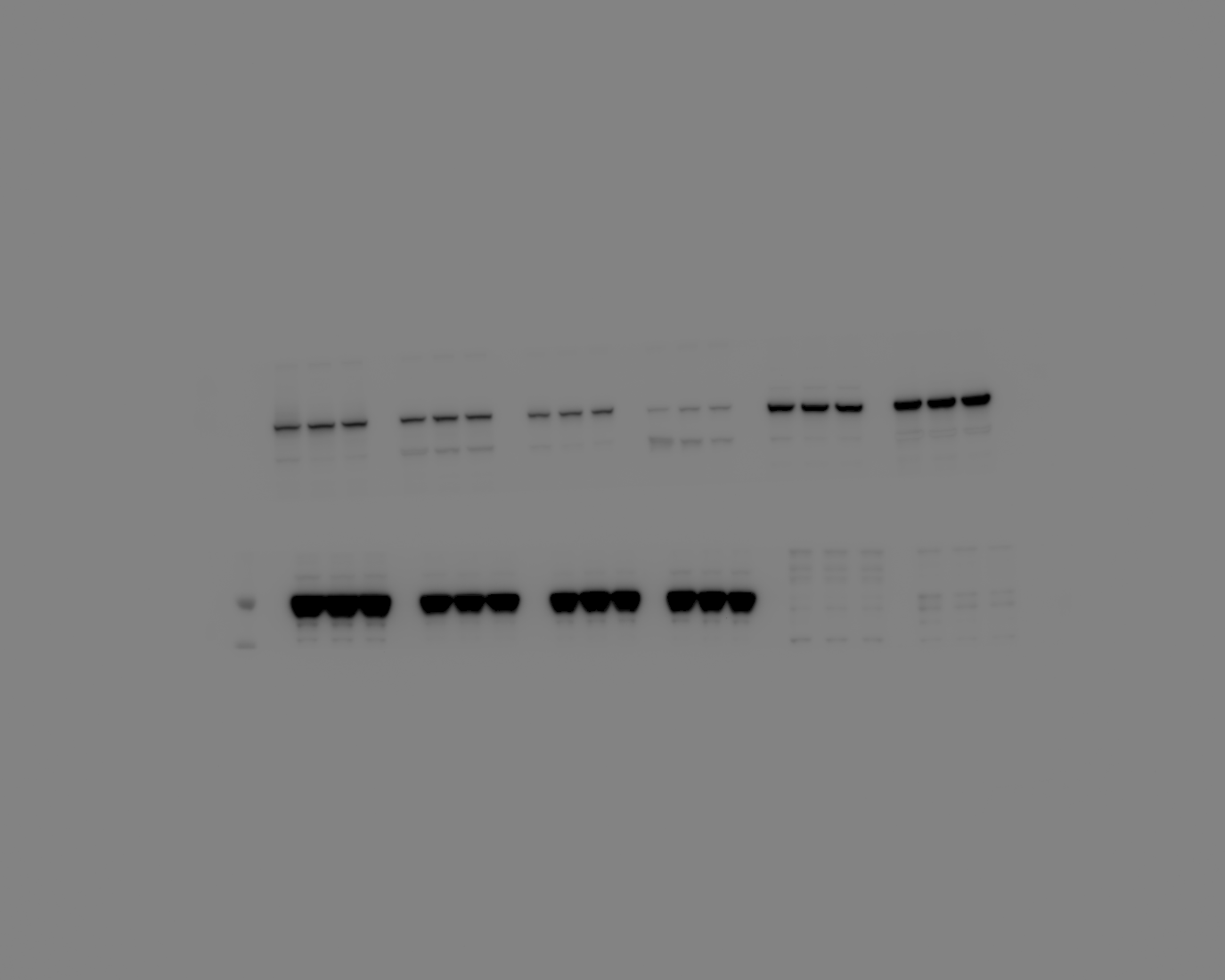

Supplement: Figure 2—source data 1. [file elife-106730-fig2-data1.zip › Figure 2ΓÇösource data 1/Figure 2A/010625-Fig2A_parp_tubulin_3(Chemiluminescence).raw16.png]

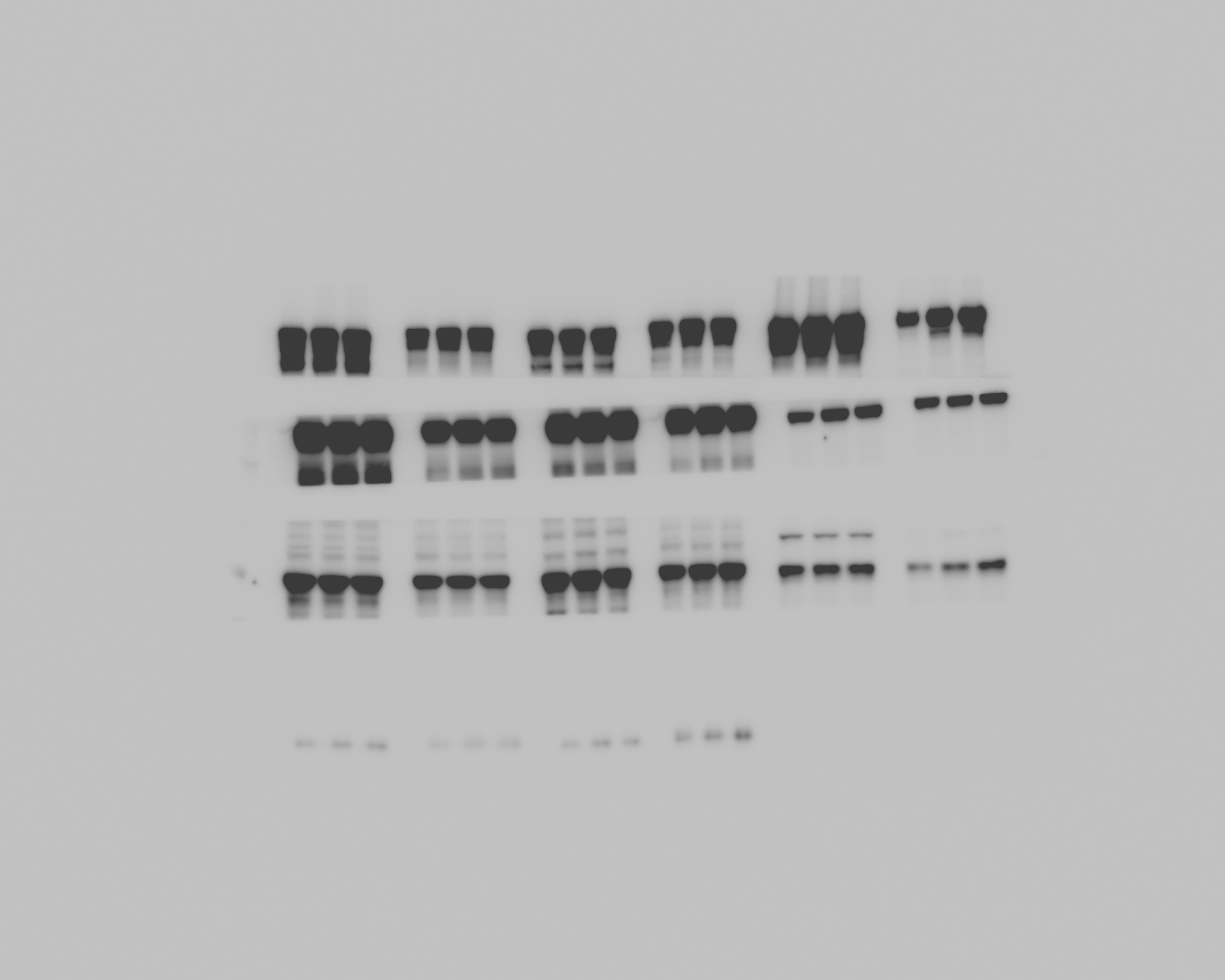

Supplement: Figure 2—source data 1. [file elife-106730-fig2-data1.zip › Figure 2ΓÇösource data 1/Figure 2A/011525-Fig2A_53bp1_usp28_p53_p21_4(Chemiluminescence_Background).tif]

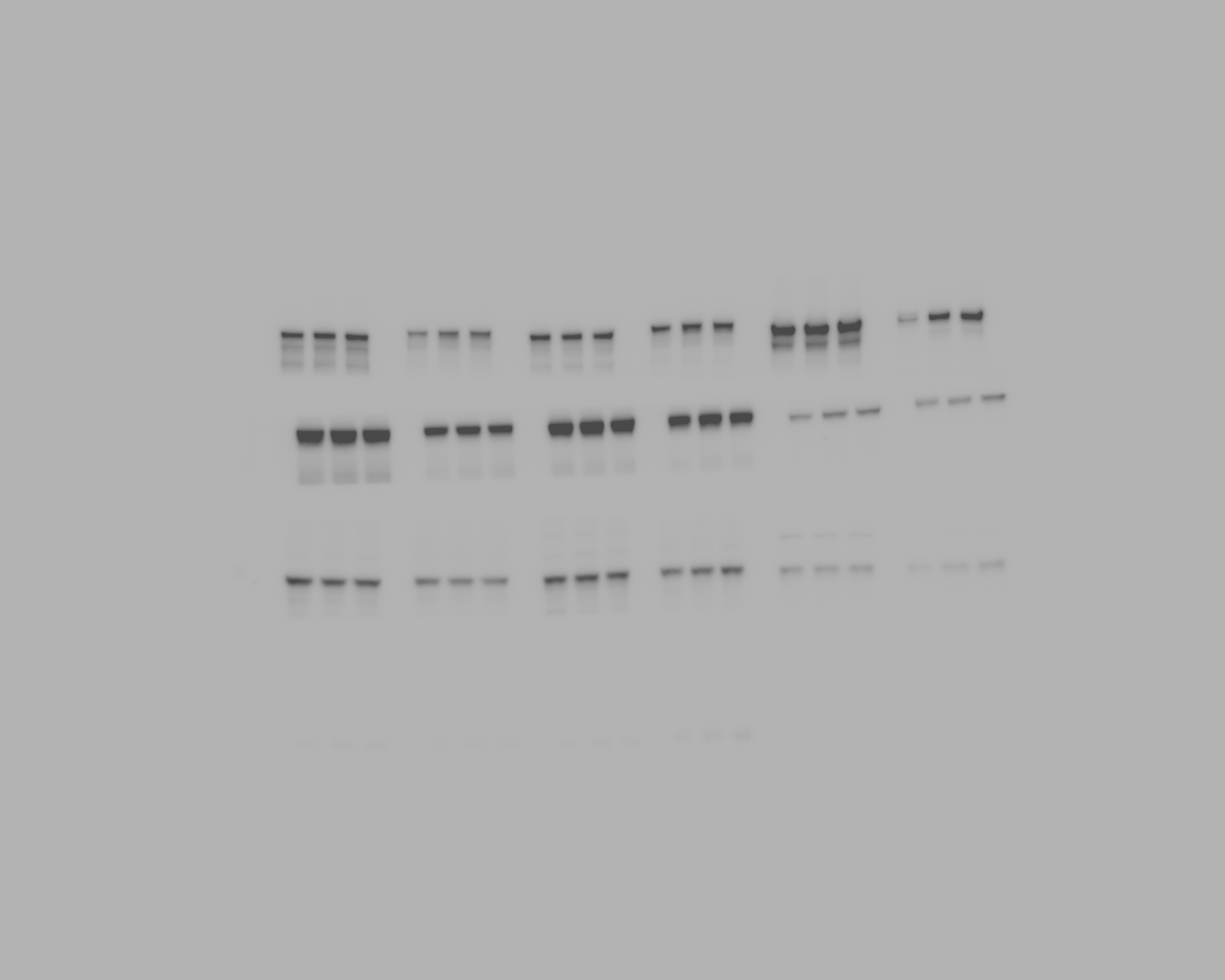

Supplement: Figure 2—source data 1. [file elife-106730-fig2-data1.zip › Figure 2ΓÇösource data 1/Figure 2A/011525-Fig2A_53bp1_usp28_p53_p21_2(Chemiluminescence_Background).tif]

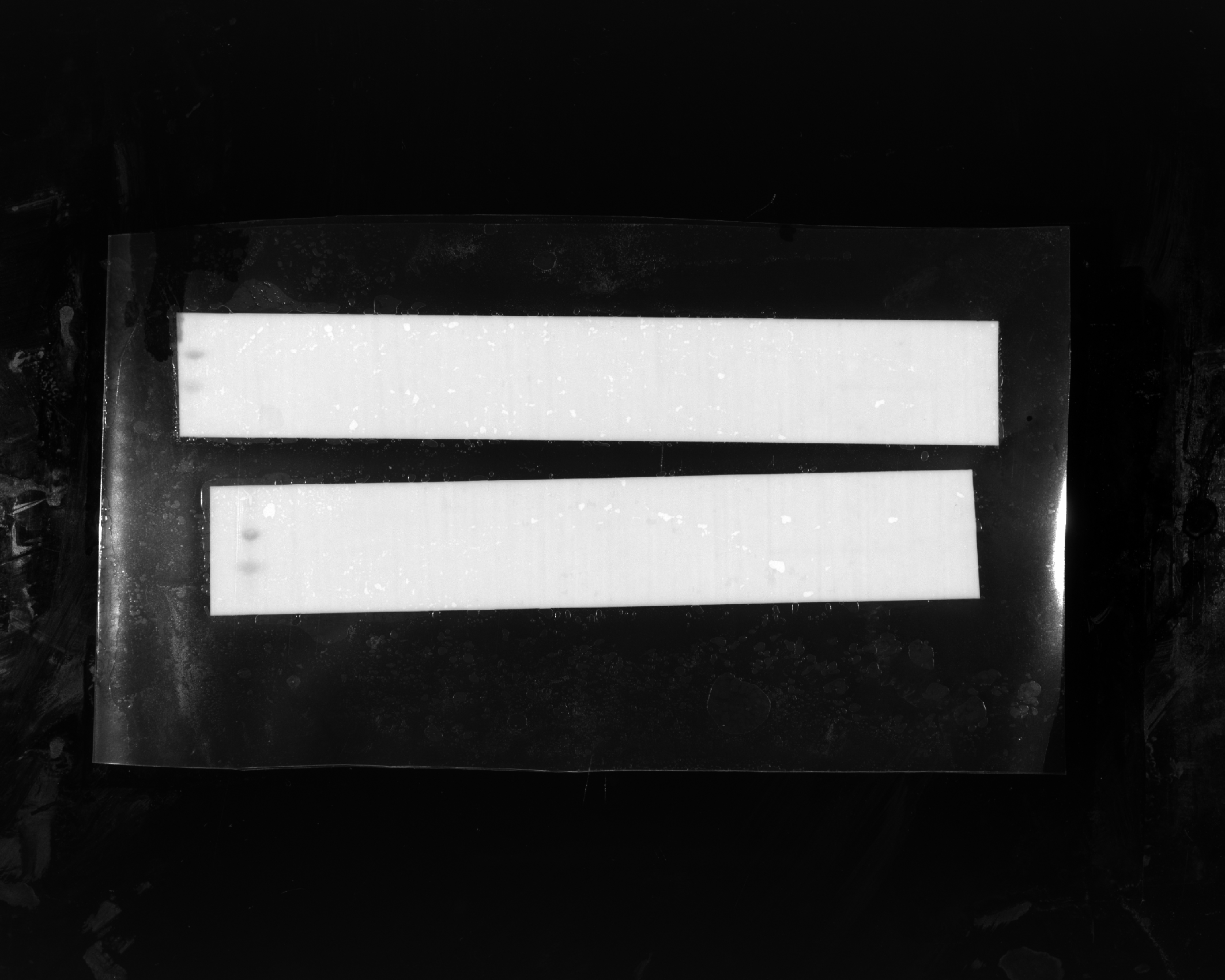

Supplement: Figure 2—source data 1. [file elife-106730-fig2-data1.zip › Figure 2ΓÇösource data 1/Figure 2A/Fig2A&B_pH3_pH3_11(Colorimetric).tif]

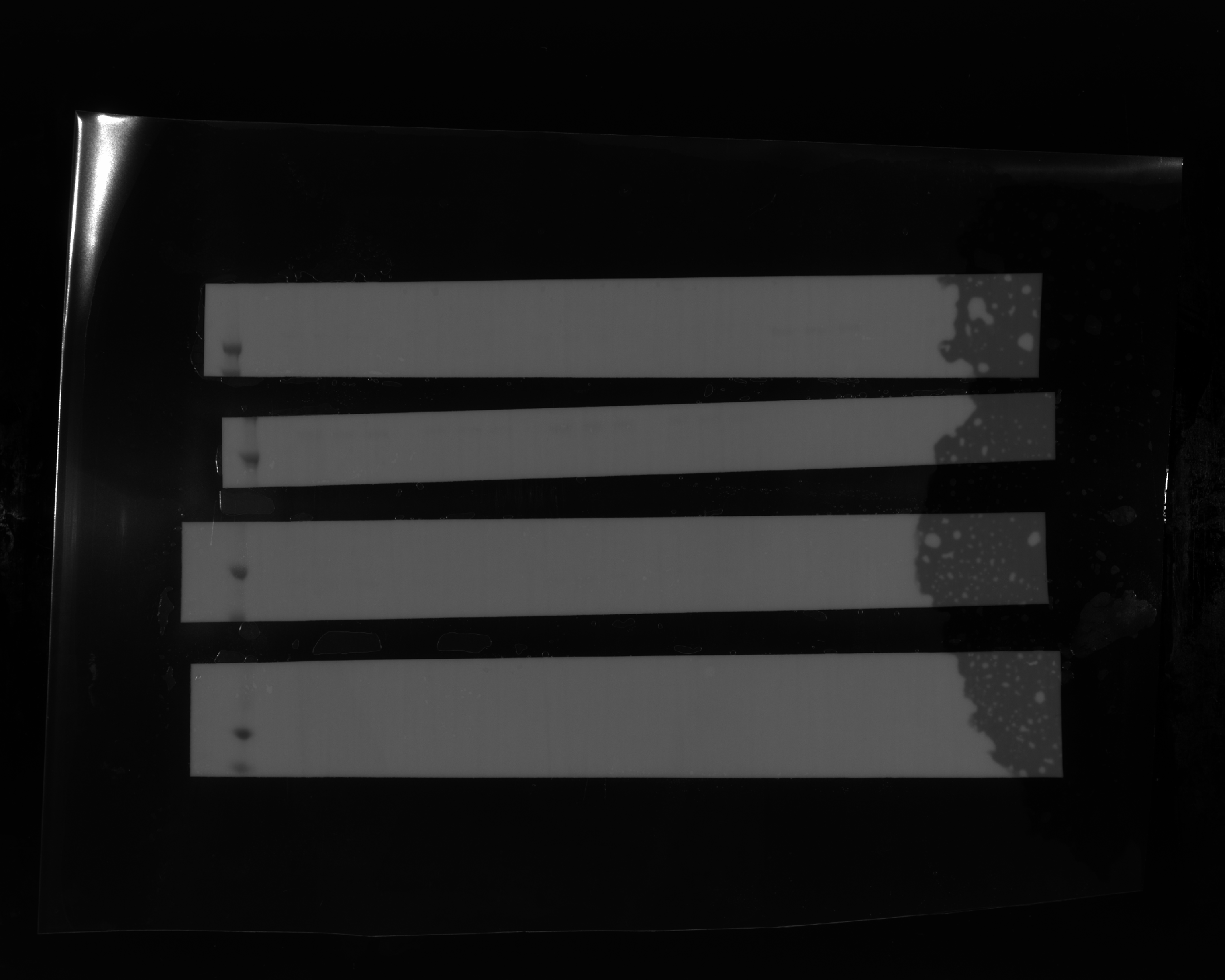

Supplement: Figure 2—source data 1. [file elife-106730-fig2-data1.zip › Figure 2ΓÇösource data 1/Figure 2A/011525-Fig2A_53bp1_usp28_p53_p21_6(Colorimetric).tif]

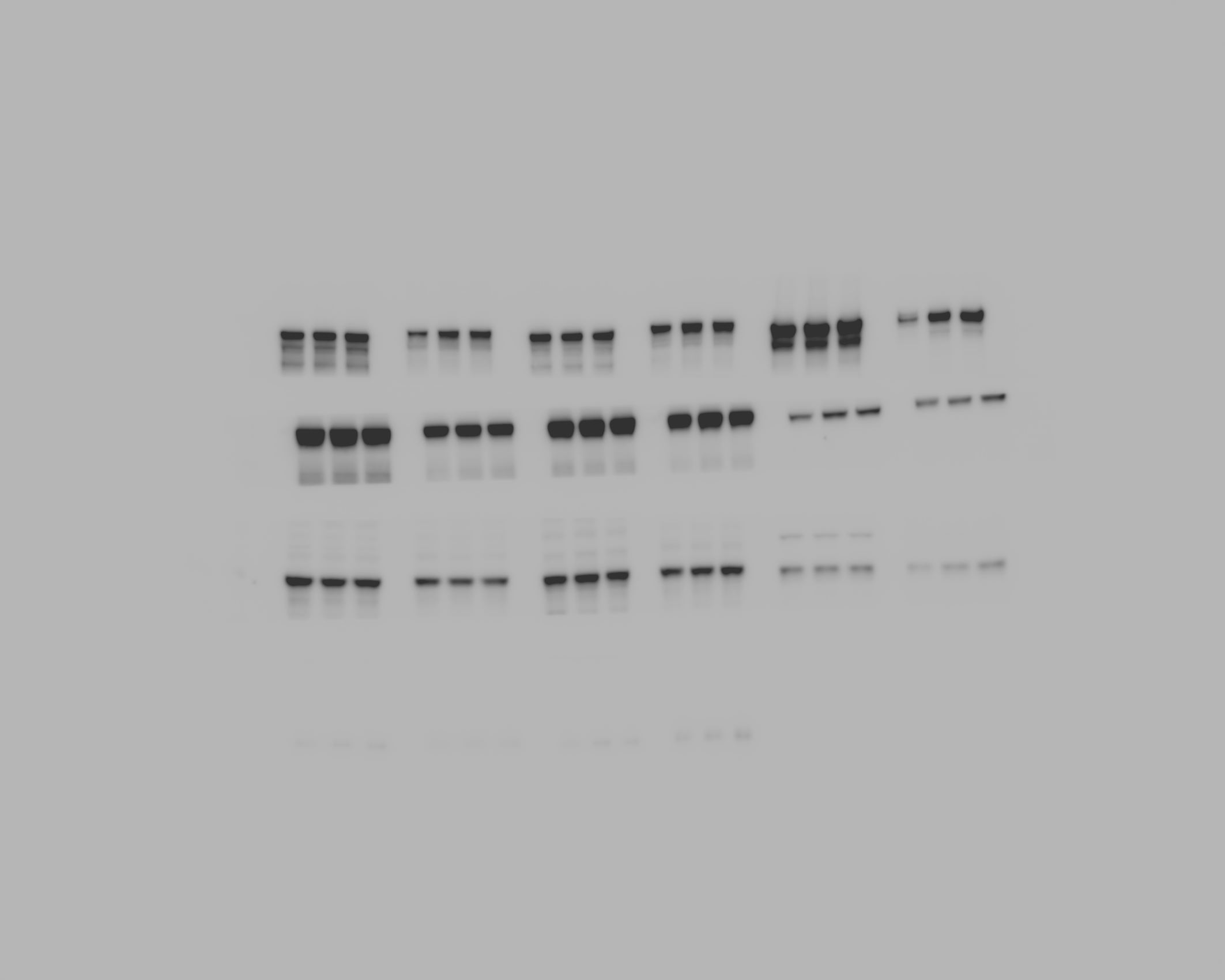

Supplement: Figure 2—source data 1. [file elife-106730-fig2-data1.zip › Figure 2ΓÇösource data 1/Figure 2A/011525-Fig2A_53bp1_usp28_p53_p21_3(Chemiluminescence_Background).tif]

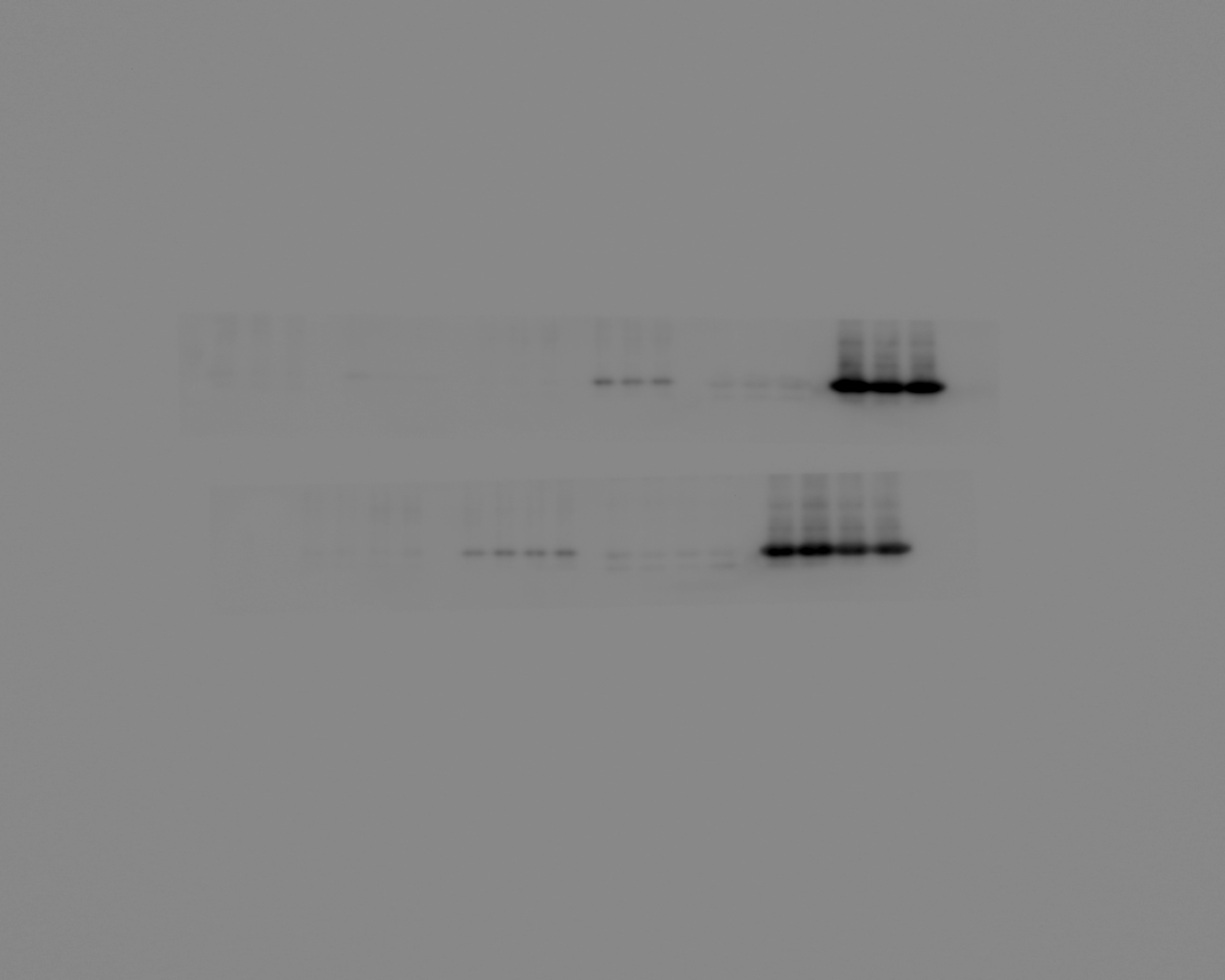

Supplement: Figure 2—source data 1. [file elife-106730-fig2-data1.zip › Figure 2ΓÇösource data 1/Figure 2A/Fig2A&B_pH3_pH3_09(Chemiluminescence).png]

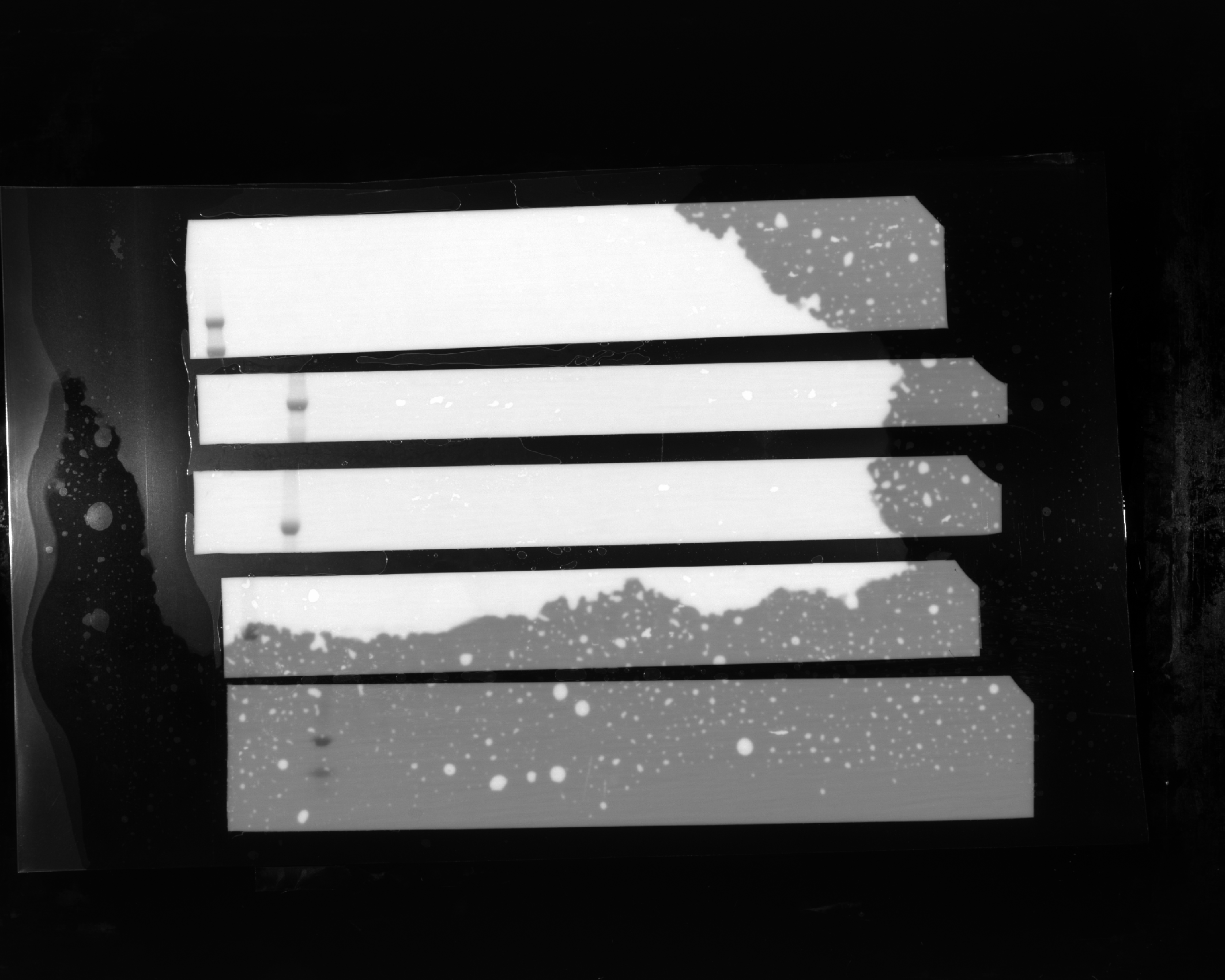

Supplement: Figure 2—source data 1. [file elife-106730-fig2-data1.zip › Figure 2ΓÇösource data 1/Figure2C(53BP1)/022625-G1-CHX_53bp1_usp28_Flag_tubulin_H3_11(Colorimetric).tif]

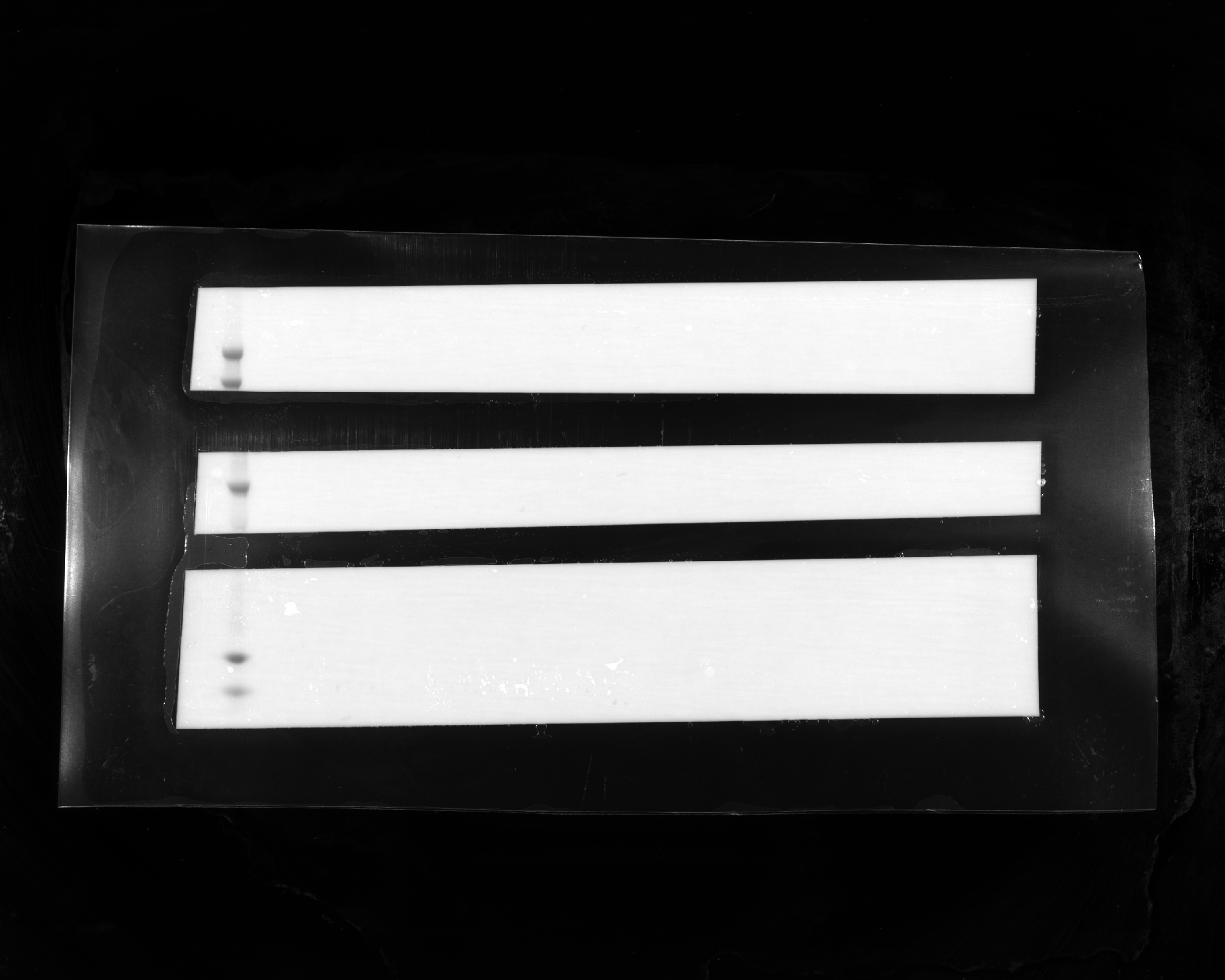

Supplement: Figure 2—source data 1. [file elife-106730-fig2-data1.zip › Figure 2ΓÇösource data 1/Figure2C(53BP1)/022525-G1-CHX_53bp1_Cul3_p21_11(Colorimetric).tif]

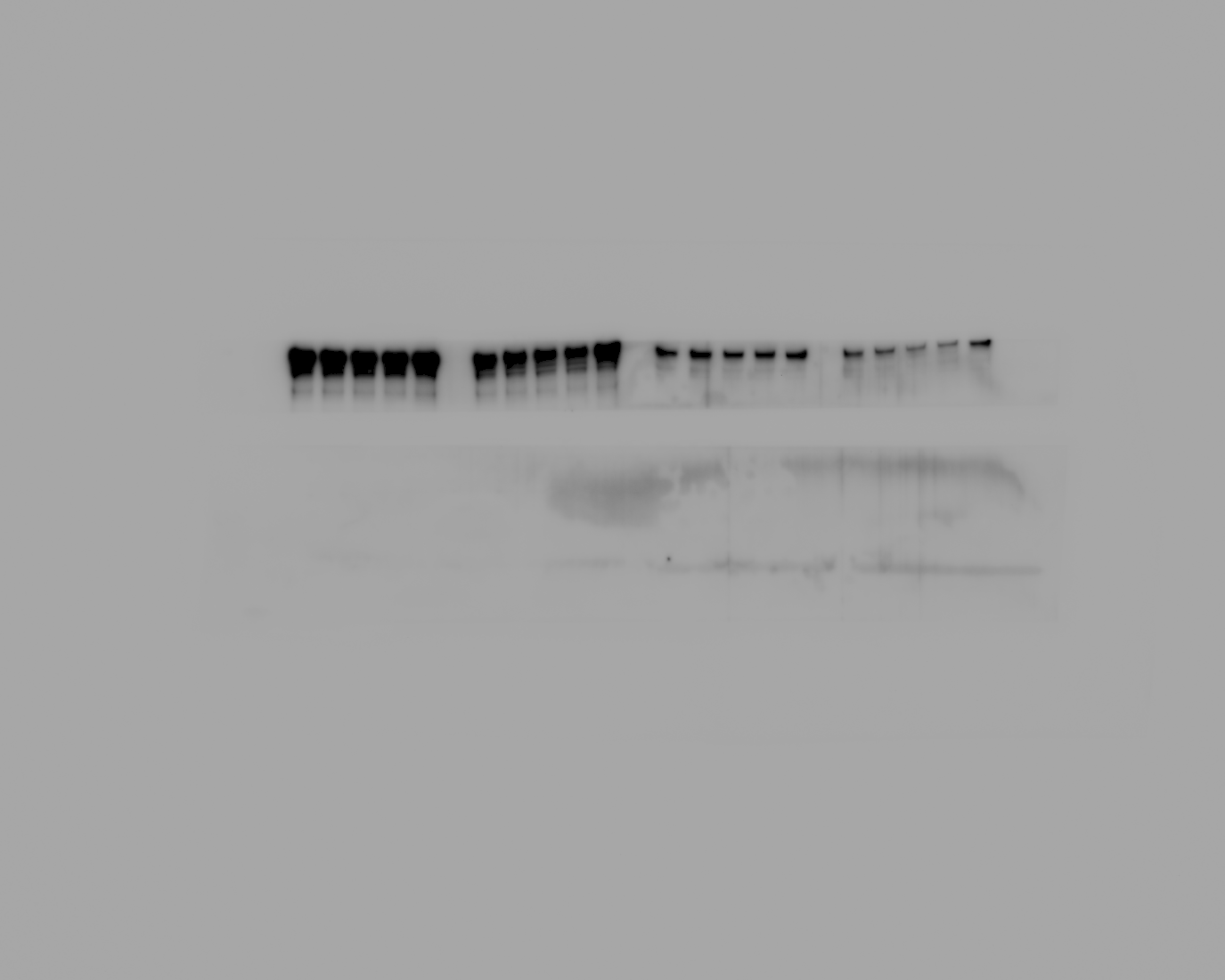

Supplement: Figure 2—source data 1. [file elife-106730-fig2-data1.zip › Figure 2ΓÇösource data 1/Figure2C(53BP1)/020425-2ndRun_53bp1_H3_5(Chemiluminescence).raw16.png]

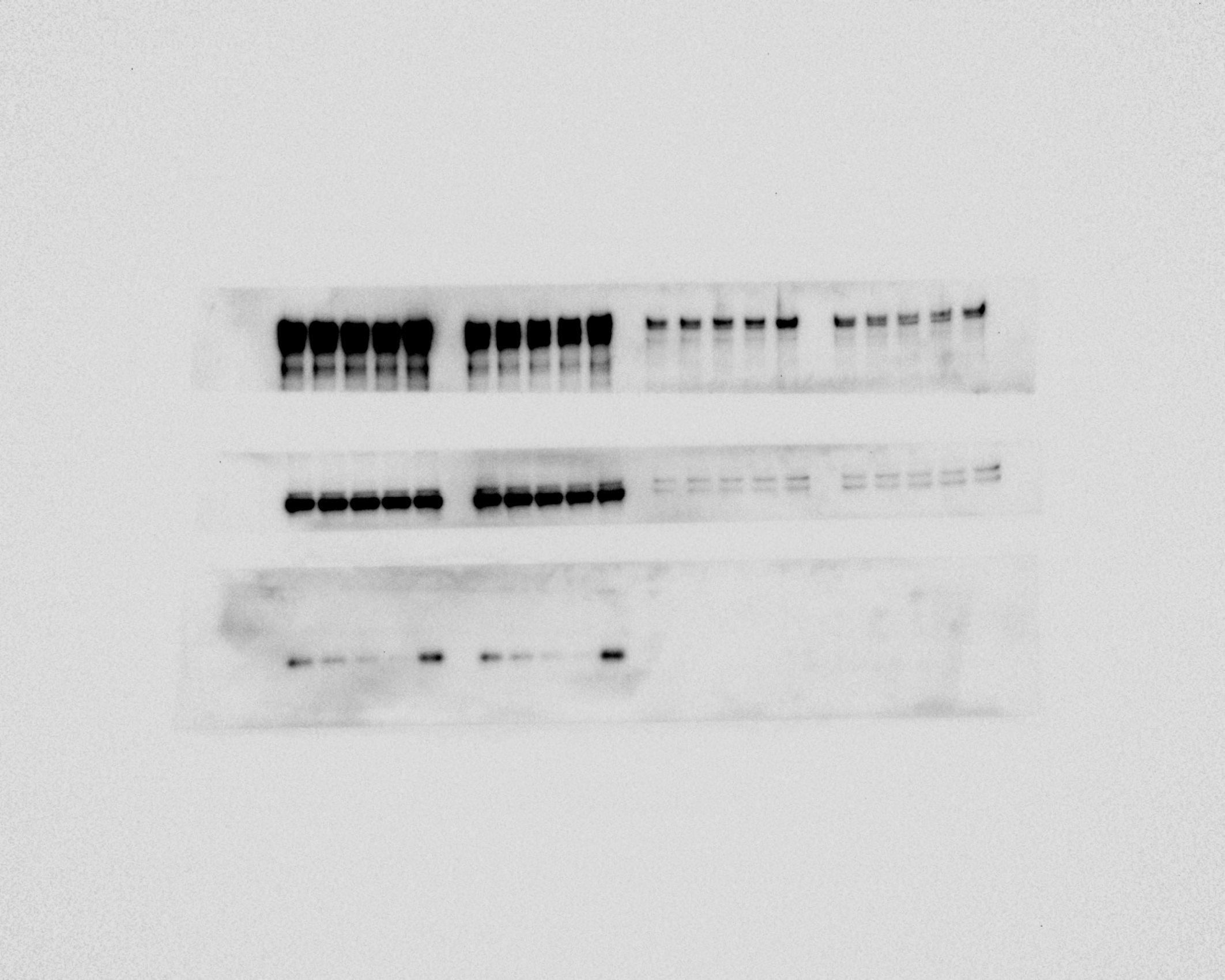

Supplement: Figure 2—source data 1. [file elife-106730-fig2-data1.zip › Figure 2ΓÇösource data 1/Figure2C(53BP1)/022525-G1-CHX_53bp1_Cul3_p21_05(Chemiluminescence).tif]

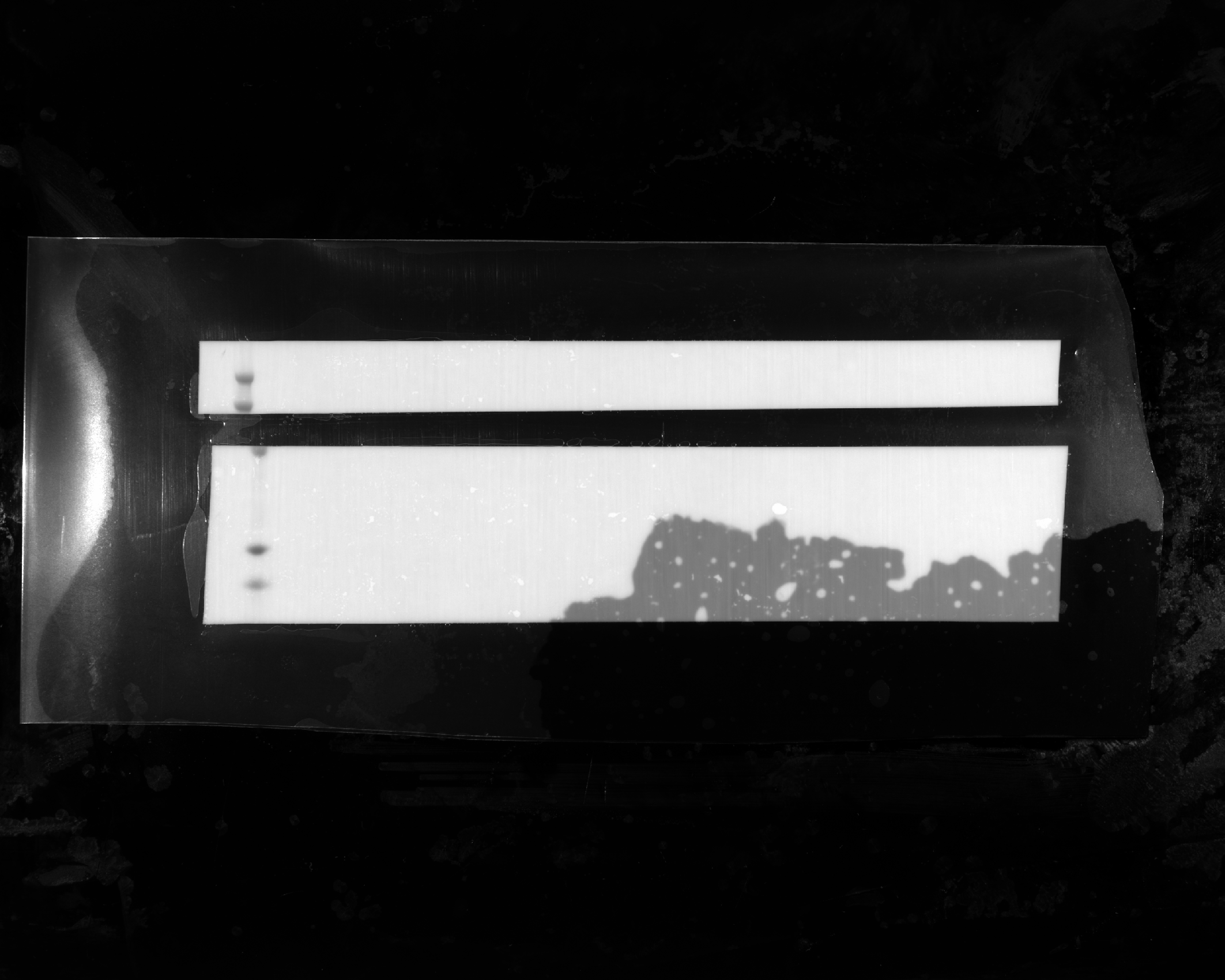

Supplement: Figure 2—source data 1. [file elife-106730-fig2-data1.zip › Figure 2ΓÇösource data 1/Figure2C(53BP1)/020425-2ndRun_53bp1_H3_6(Colorimetric).tif]

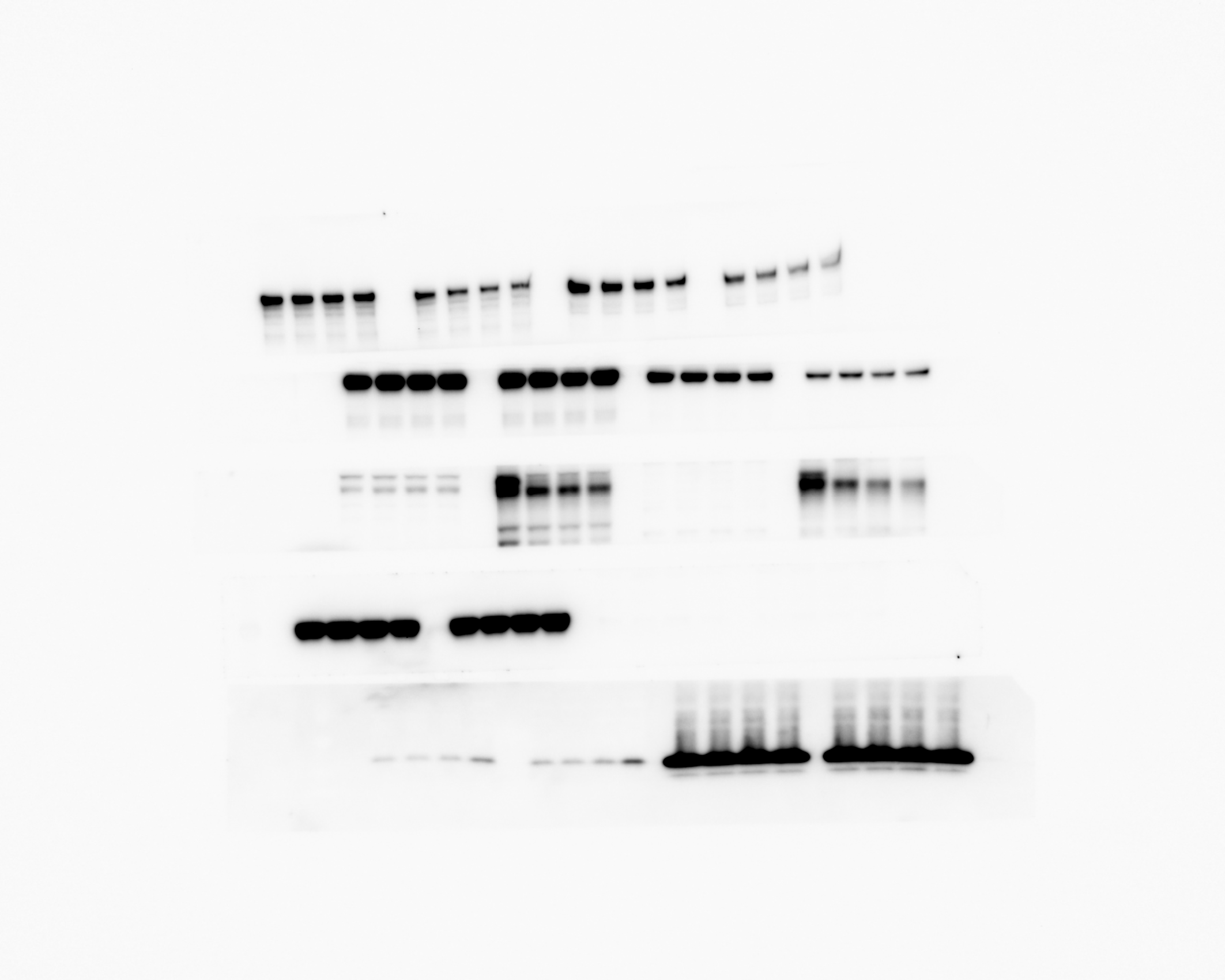

Supplement: Figure 2—source data 1. [file elife-106730-fig2-data1.zip › Figure 2ΓÇösource data 1/Figure2C(53BP1)/022625-G1-CHX_53bp1_usp28_Flag_tubulin_H3_04(Chemiluminescence).tif]

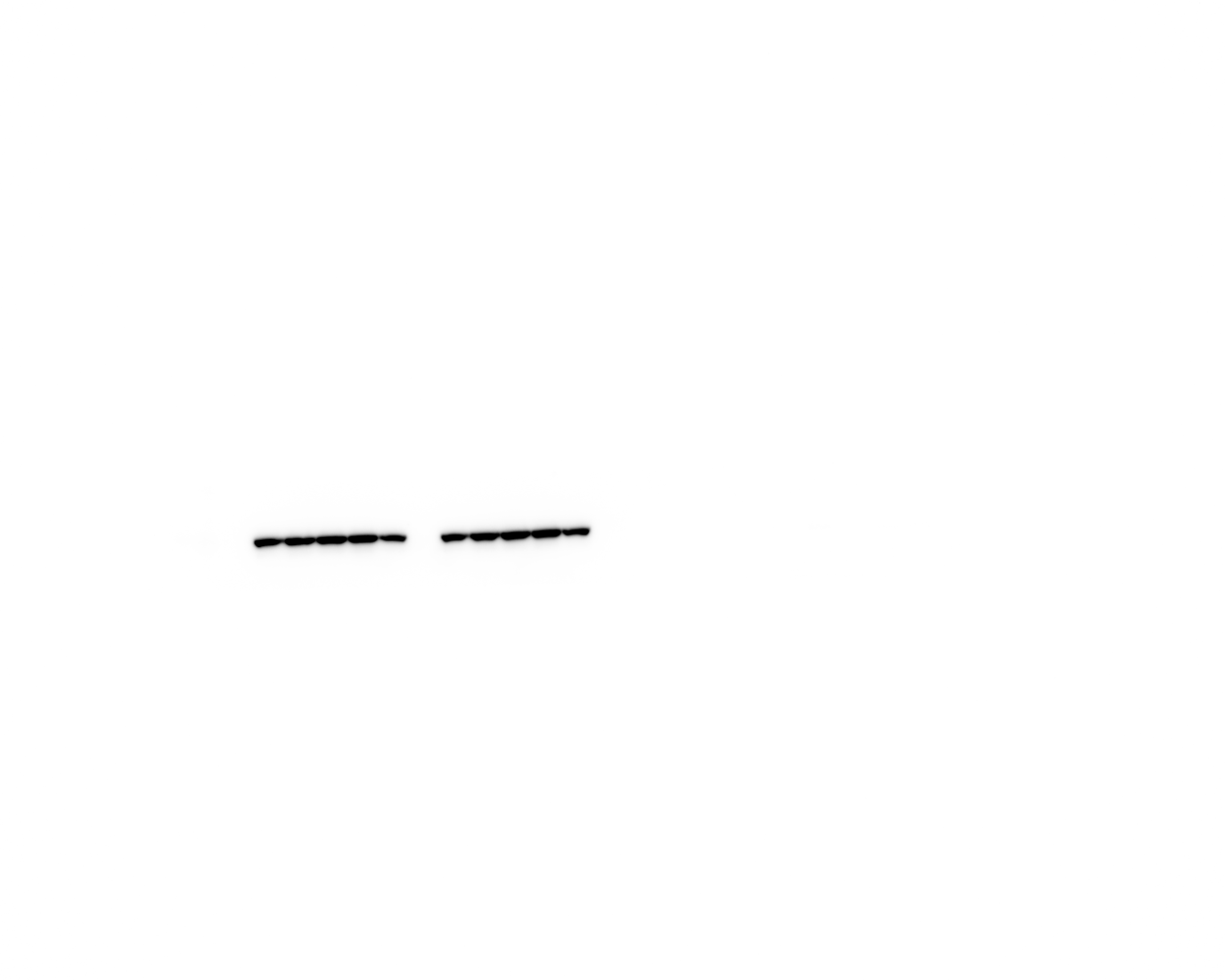

Supplement: Figure 2—source data 1. [file elife-106730-fig2-data1.zip › Figure 2ΓÇösource data 1/Figure 2C/020425-G1_tubulin_5(Chemiluminescence).raw16.tif]

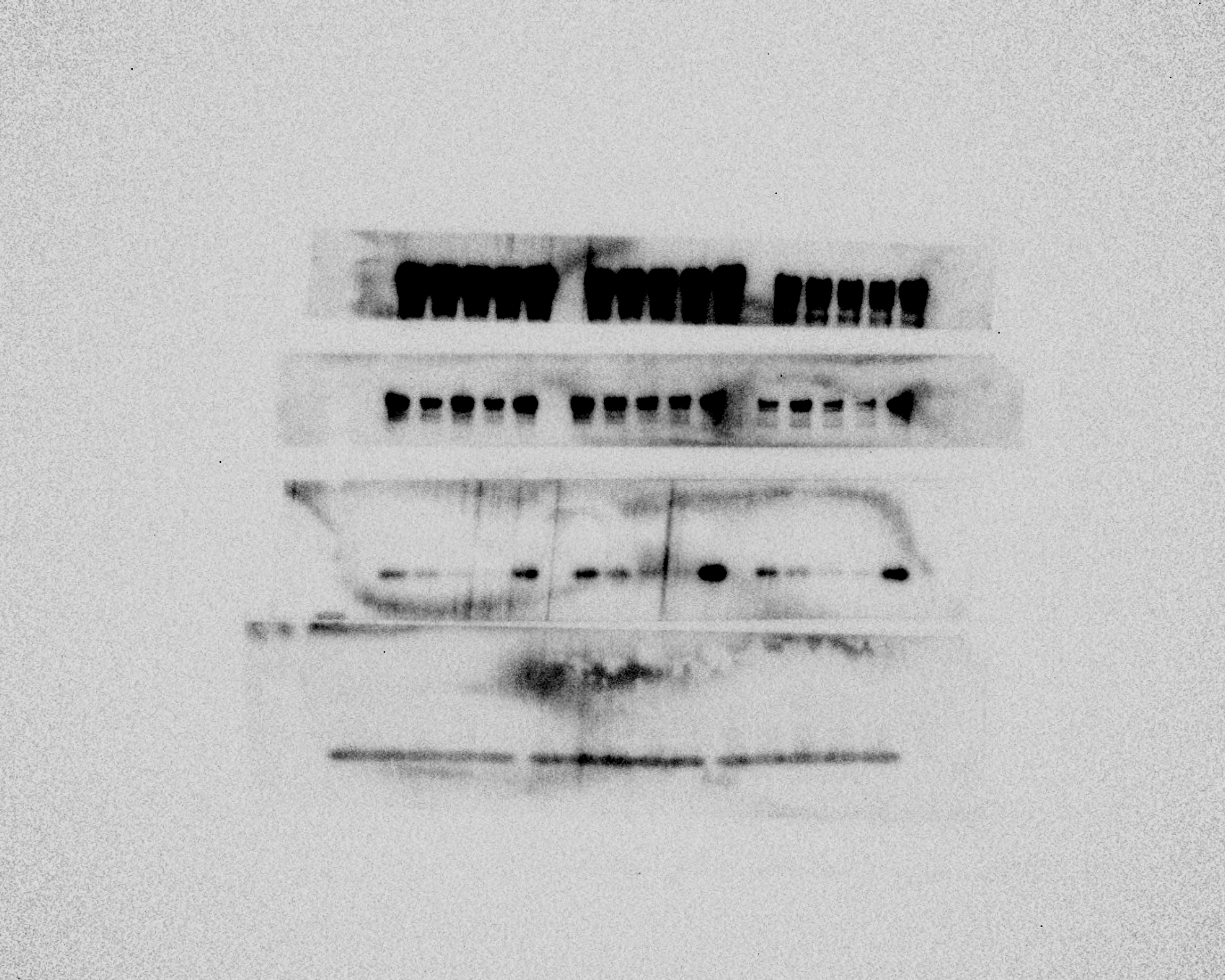

Supplement: Figure 2—source data 1. [file elife-106730-fig2-data1.zip › Figure 2ΓÇösource data 1/Figure 2C/020425-G1_CSK-Chro_53bp1_CSK_p21_Chro_H3_05(Chemiluminescence).tif]

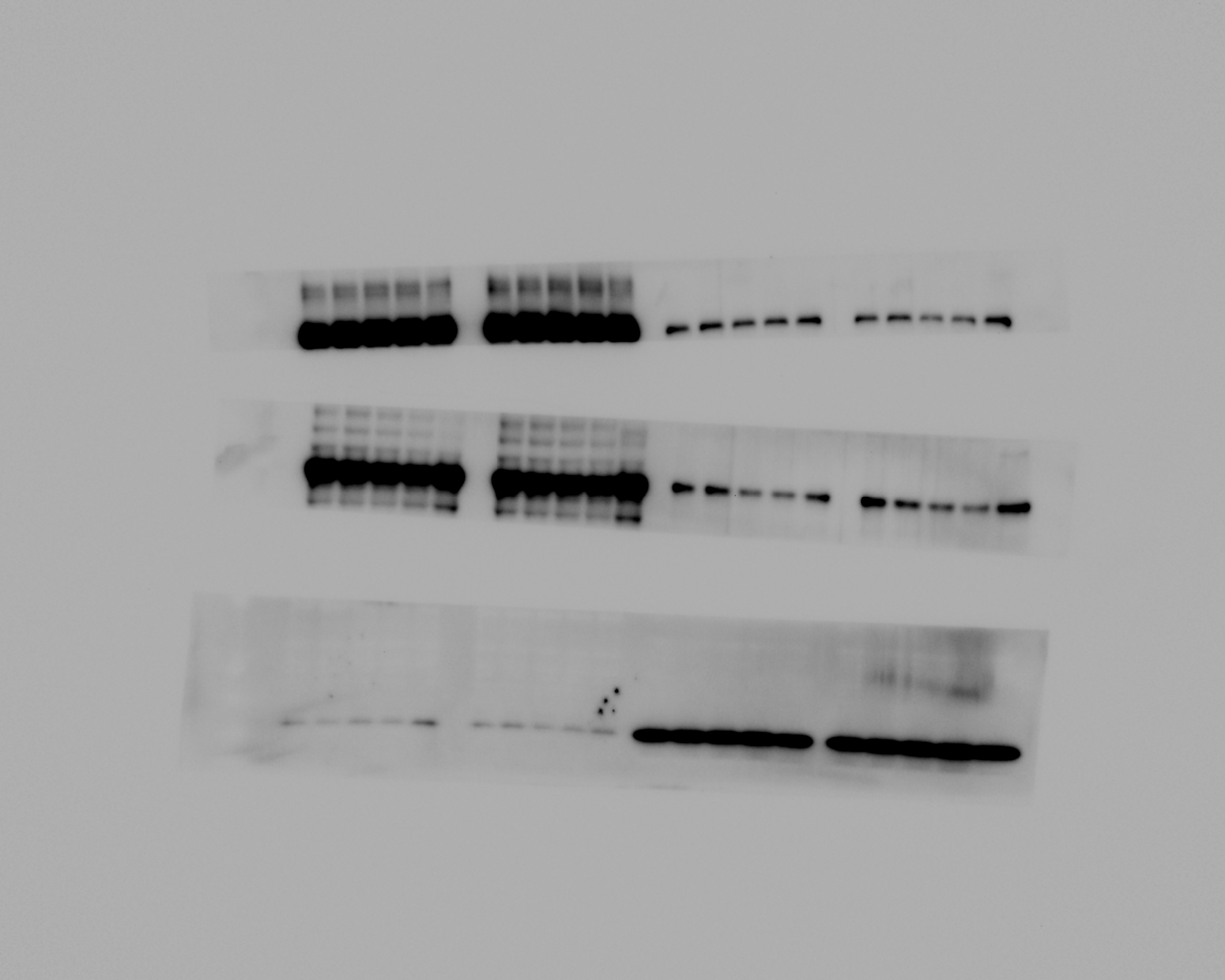

Supplement: Figure 2—source data 1. [file elife-106730-fig2-data1.zip › Figure 2ΓÇösource data 1/Figure 2C/020425-2ndrun_usp28_p53_H3_4(Chemiluminescence).tif]

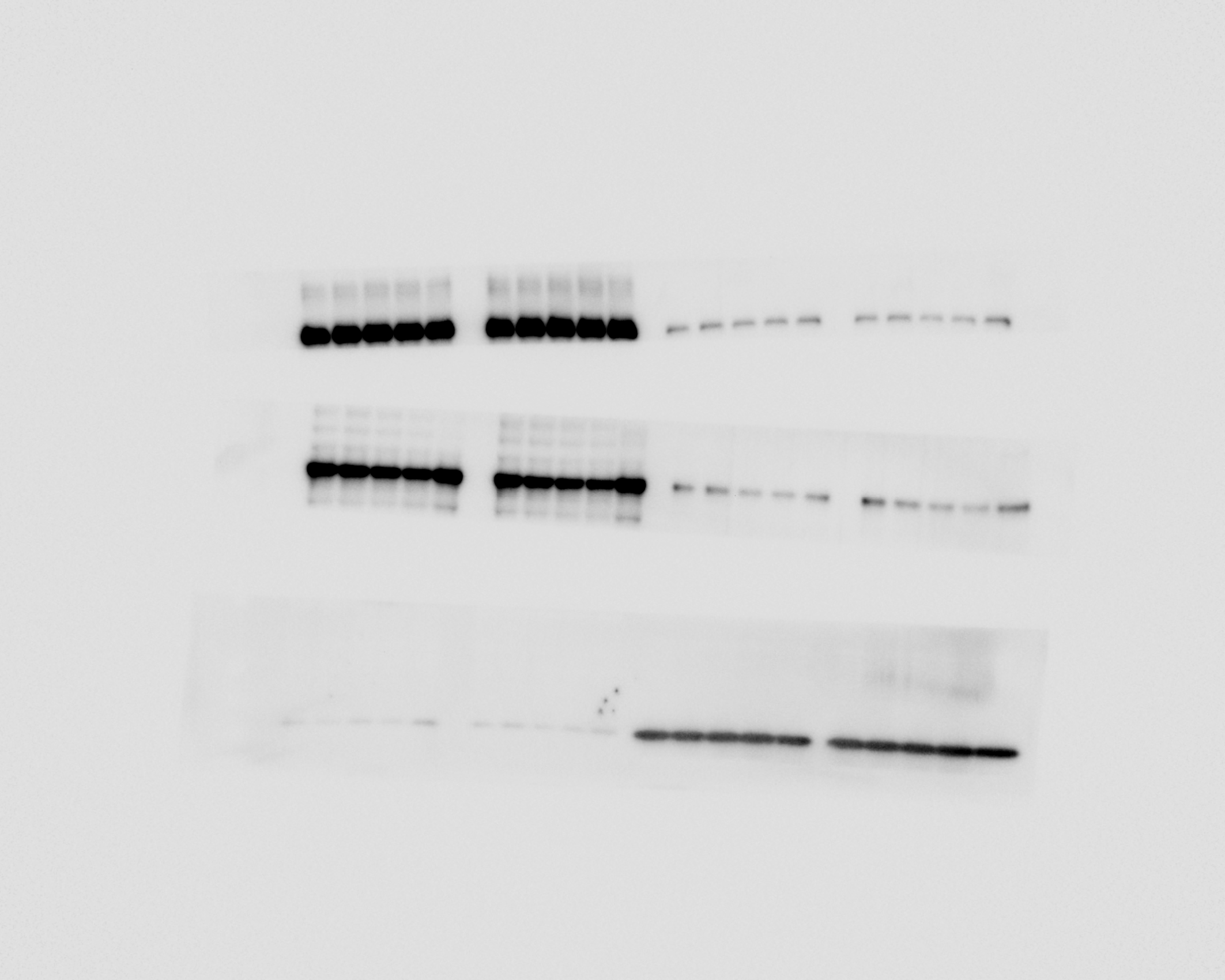

Supplement: Figure 2—source data 1. [file elife-106730-fig2-data1.zip › Figure 2ΓÇösource data 1/Figure 2C/020425-2ndrun_usp28_p53_H3_2(Chemiluminescence).tif]

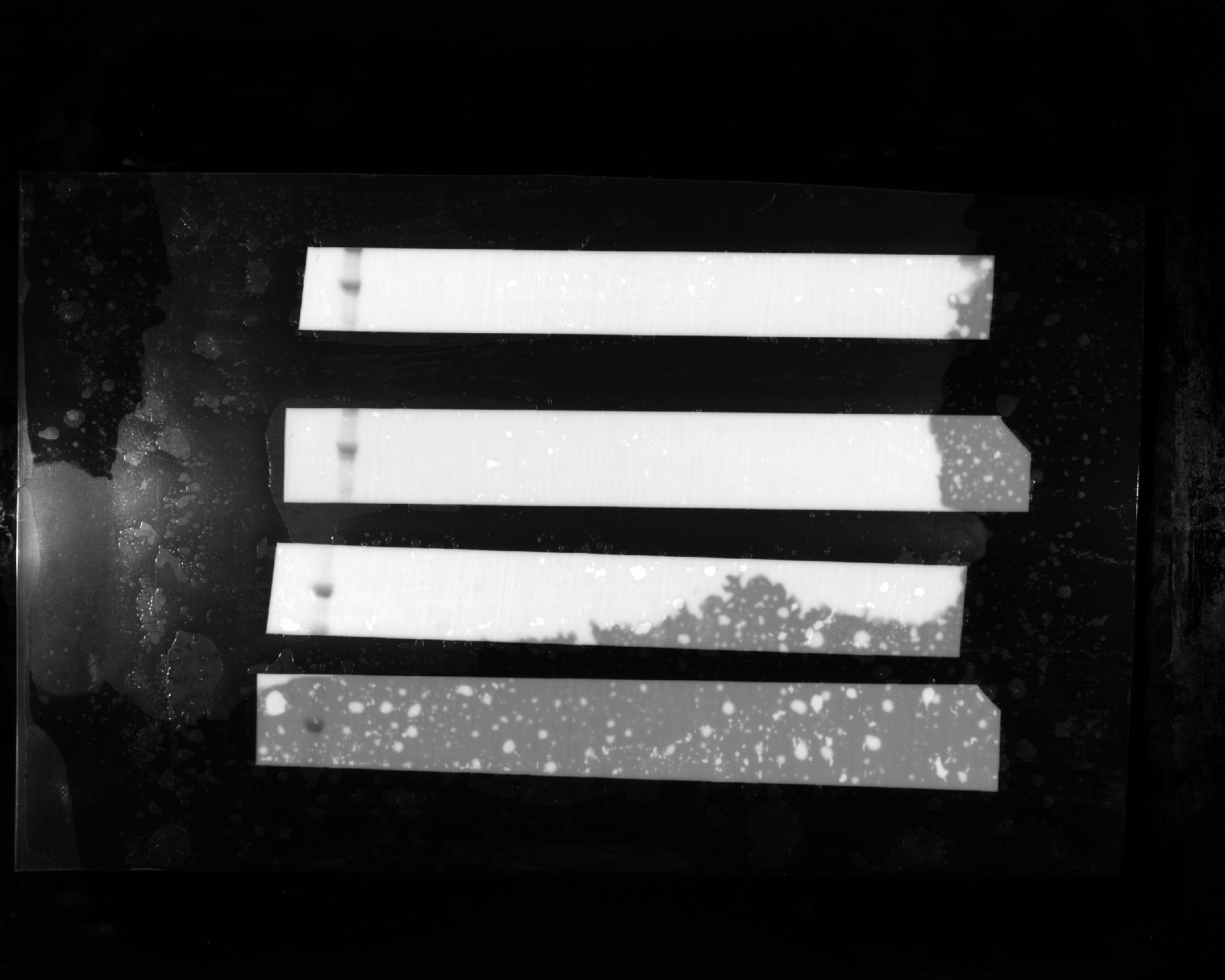

Supplement: Figure 2—source data 1. [file elife-106730-fig2-data1.zip › Figure 2ΓÇösource data 1/Figure 2C/020425-G1_Csk-Chro_cul3_gmcl1_6(Colorimetric).tif]

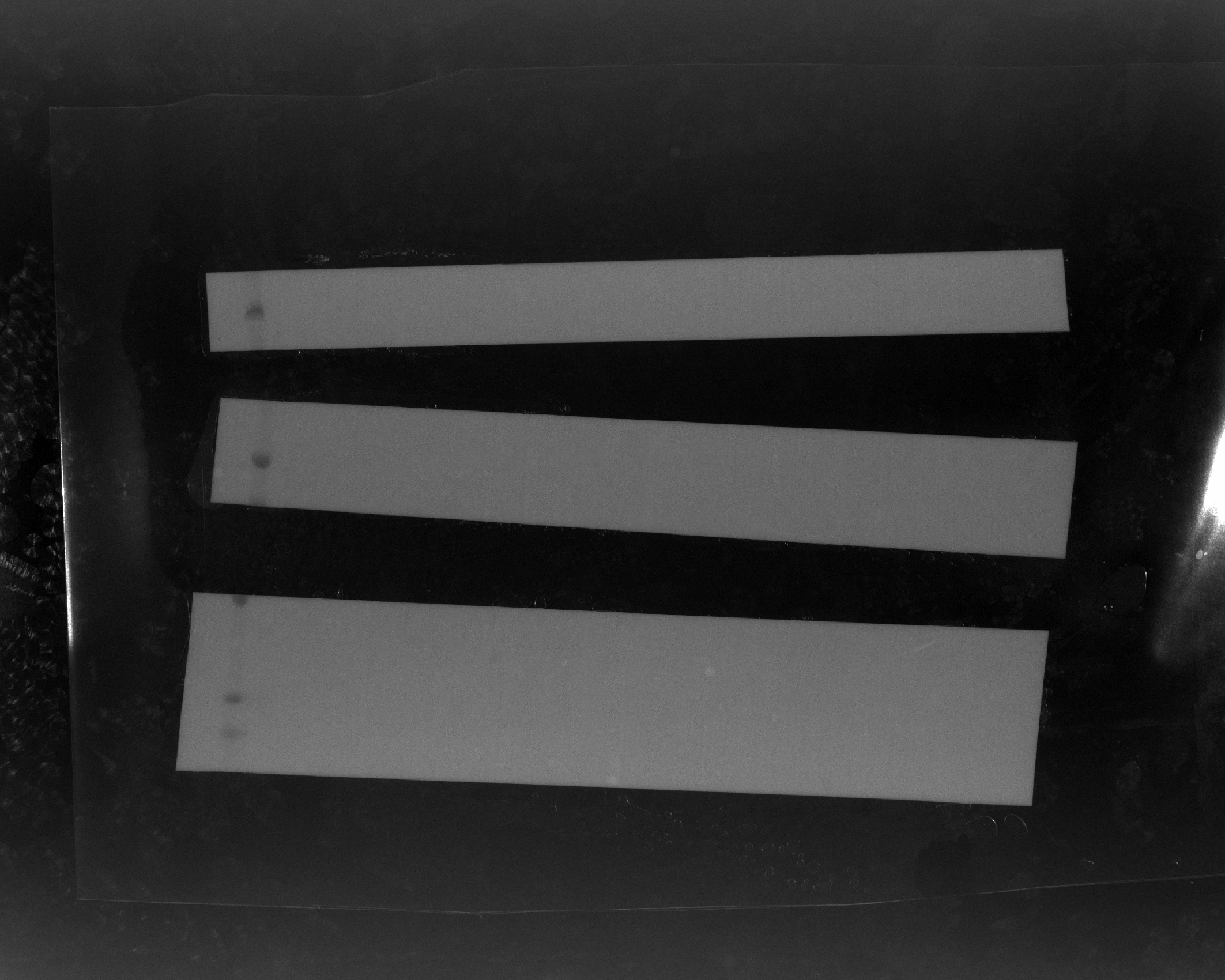

Supplement: Figure 2—source data 1. [file elife-106730-fig2-data1.zip › Figure 2ΓÇösource data 1/Figure 2C/020425-2ndrun_usp28_p53_H3_6(Colorimetric).tif]

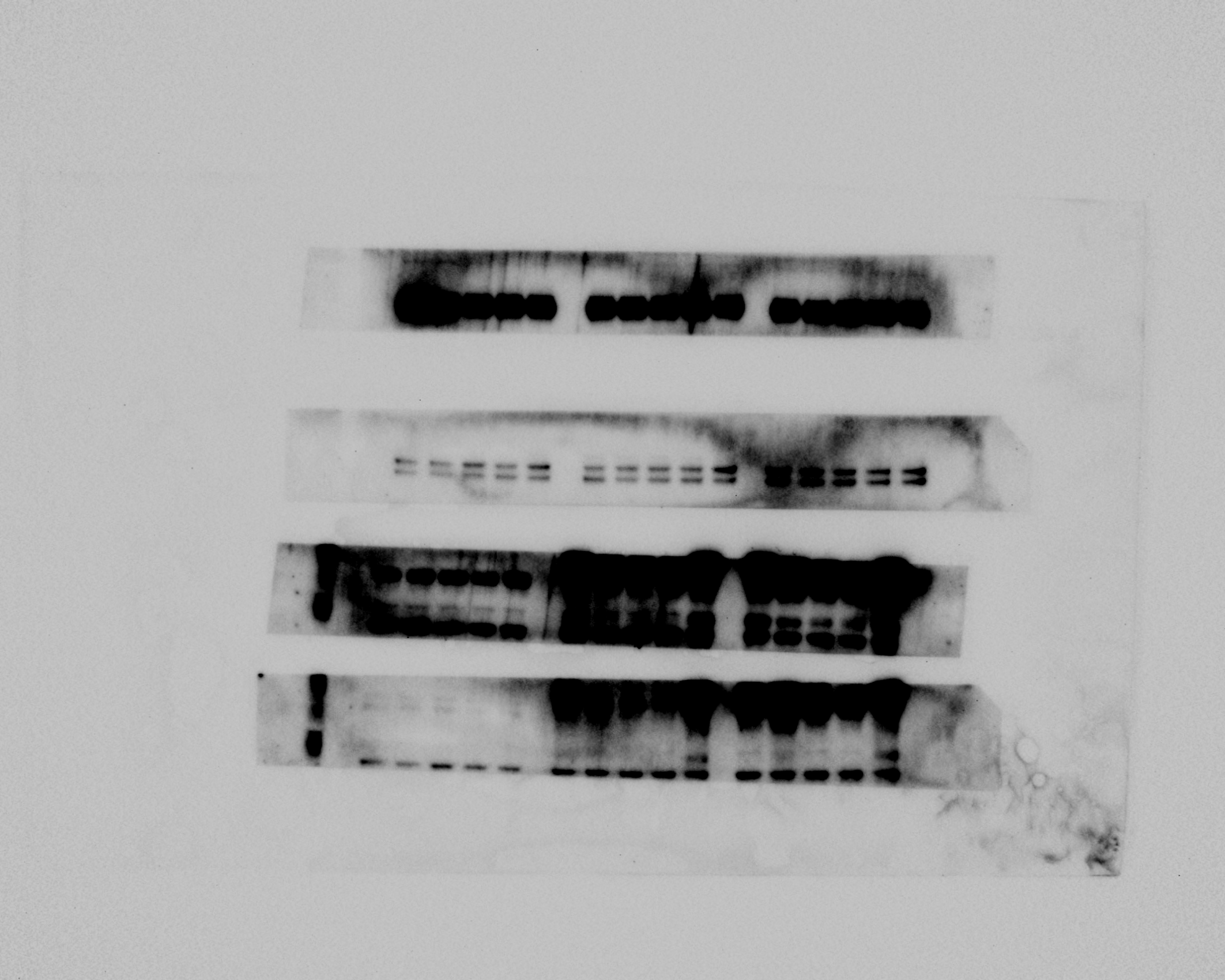

Supplement: Figure 2—source data 1. [file elife-106730-fig2-data1.zip › Figure 2ΓÇösource data 1/Figure 2C/020425-G1_Csk-Chro_cul3_gmcl1_5(Chemiluminescence).tif]

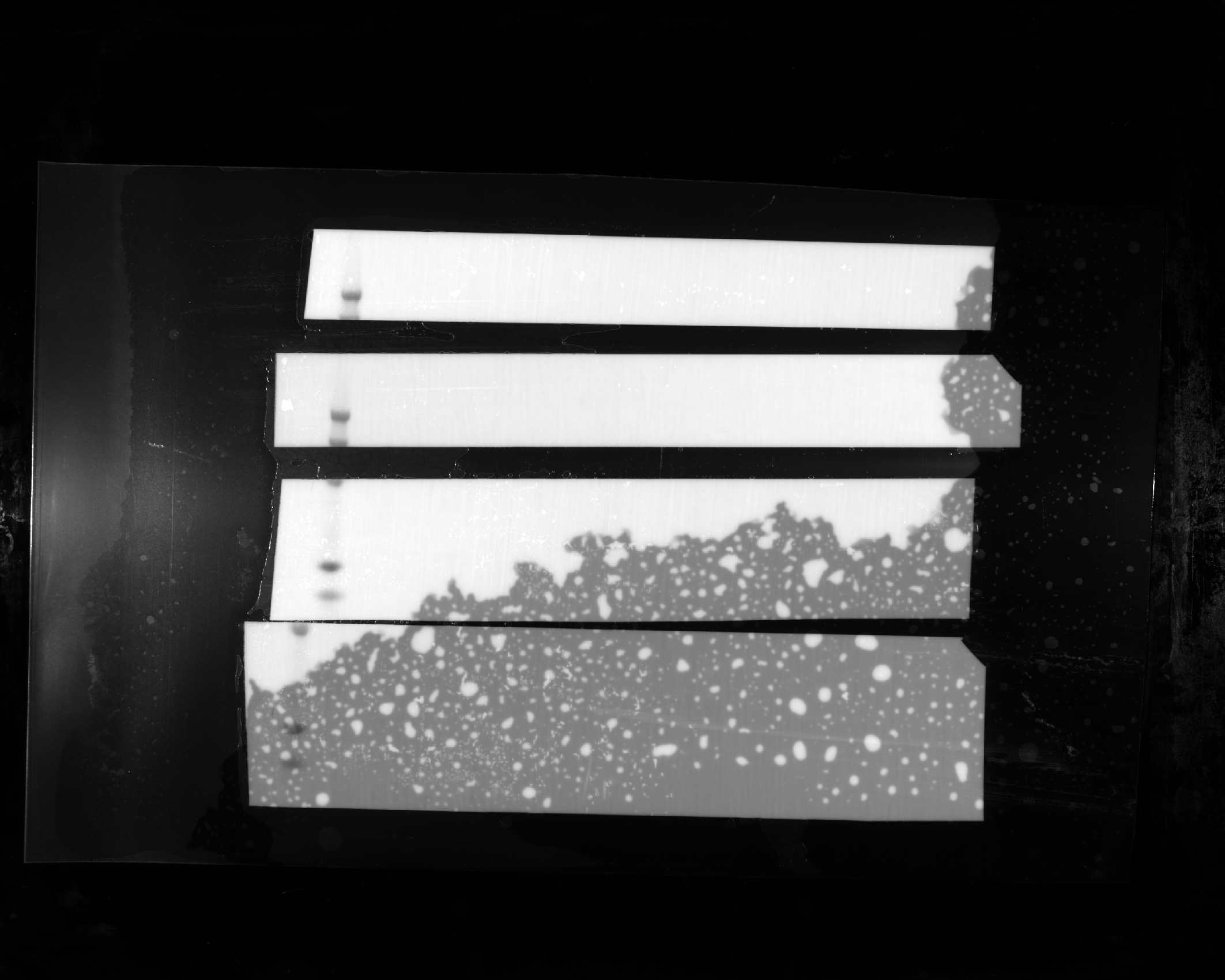

Supplement: Figure 2—source data 1. [file elife-106730-fig2-data1.zip › Figure 2ΓÇösource data 1/Figure 2C/020425-G1_CSK-Chro_53bp1_CSK_p21_Chro_H3_11(Colorimetric).tif]

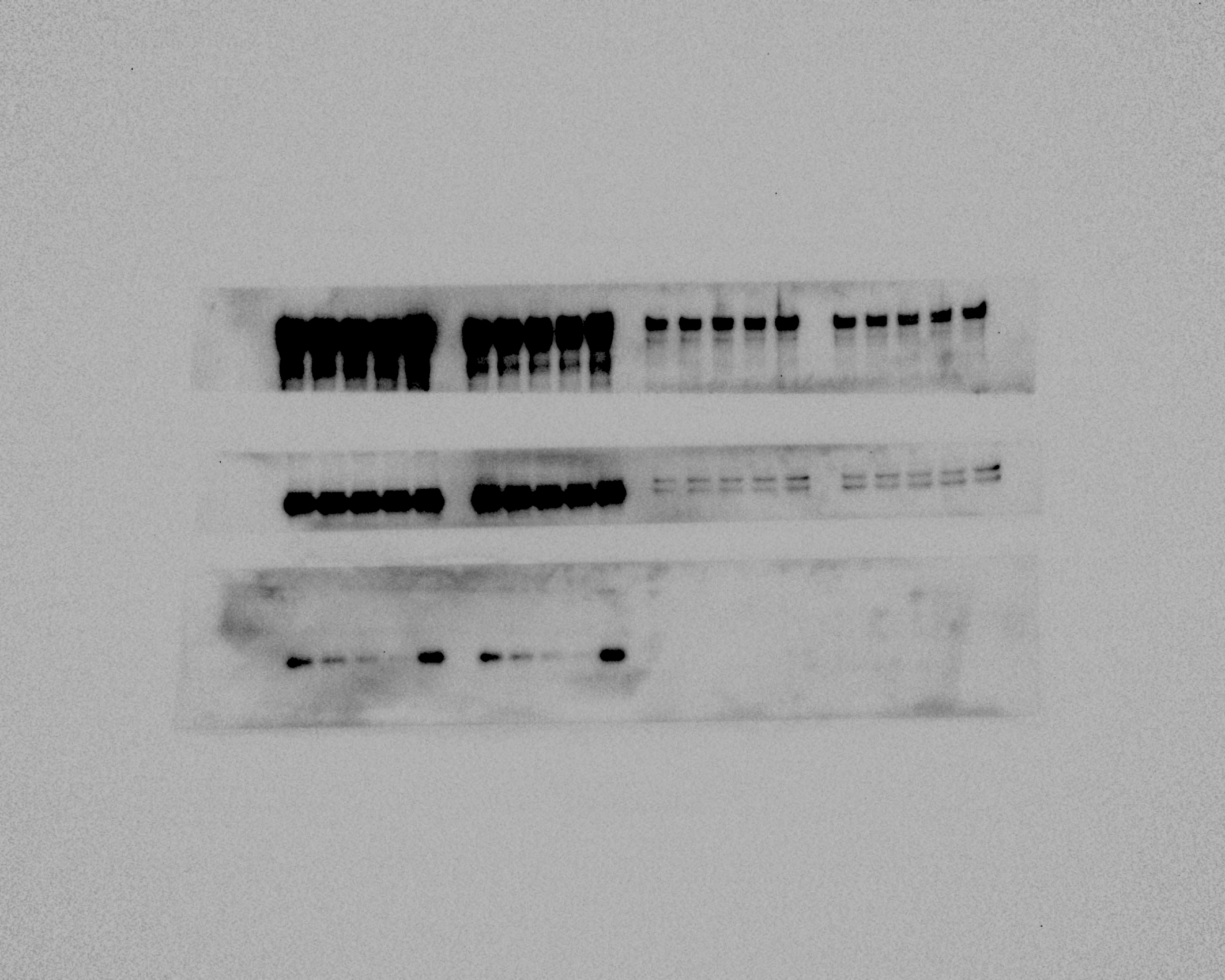

Supplement: Figure 2—source data 1. [file elife-106730-fig2-data1.zip › Figure 2ΓÇösource data 1/Figure 2C/022525-G1-CHX_53bp1_Cul3_p21_05(Chemiluminescence).tif]

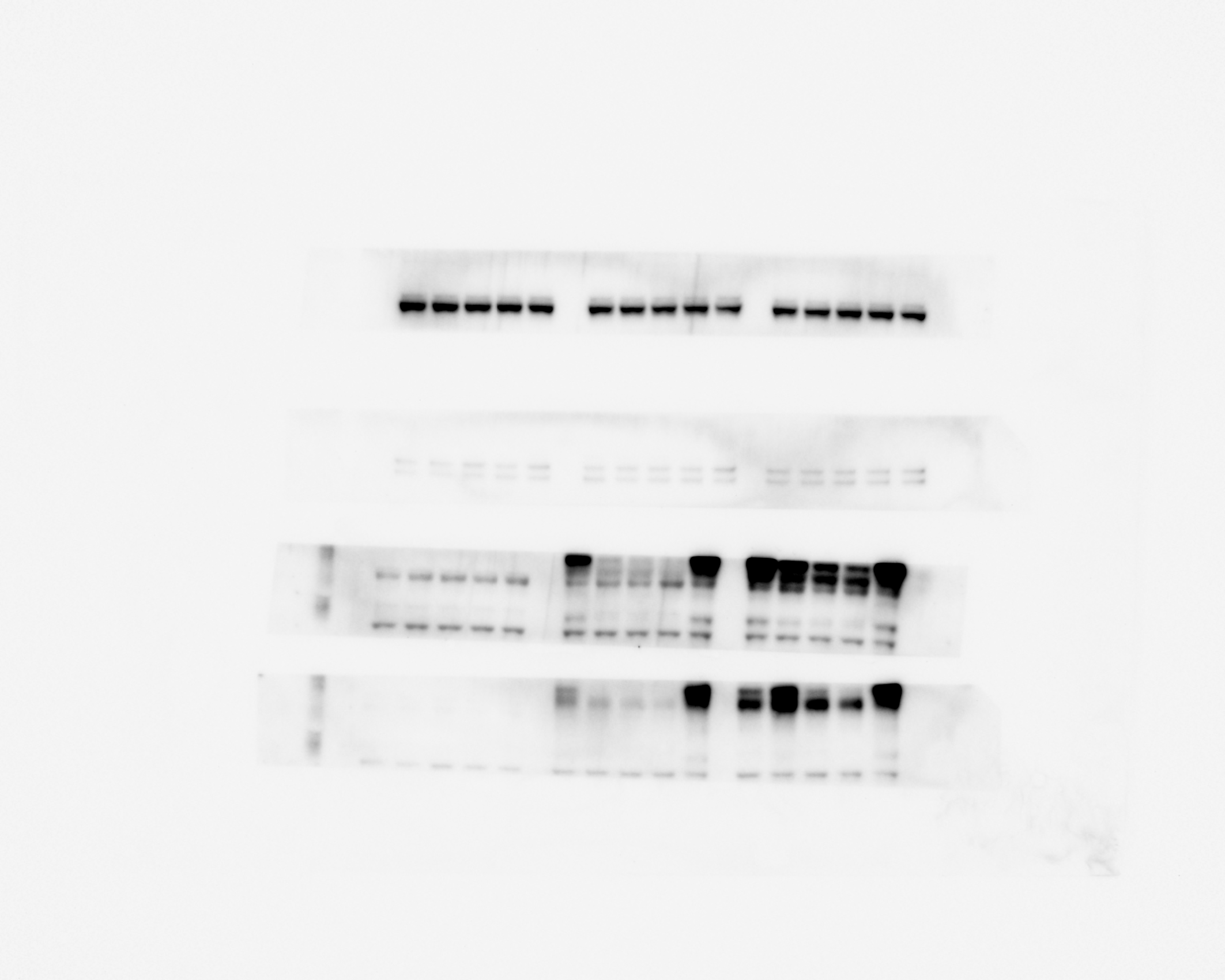

Supplement: Figure 2—source data 1. [file elife-106730-fig2-data1.zip › Figure 2ΓÇösource data 1/Figure 2C/020425-G1_Csk-Chro_cul3_gmcl1_3(Chemiluminescence).tif]

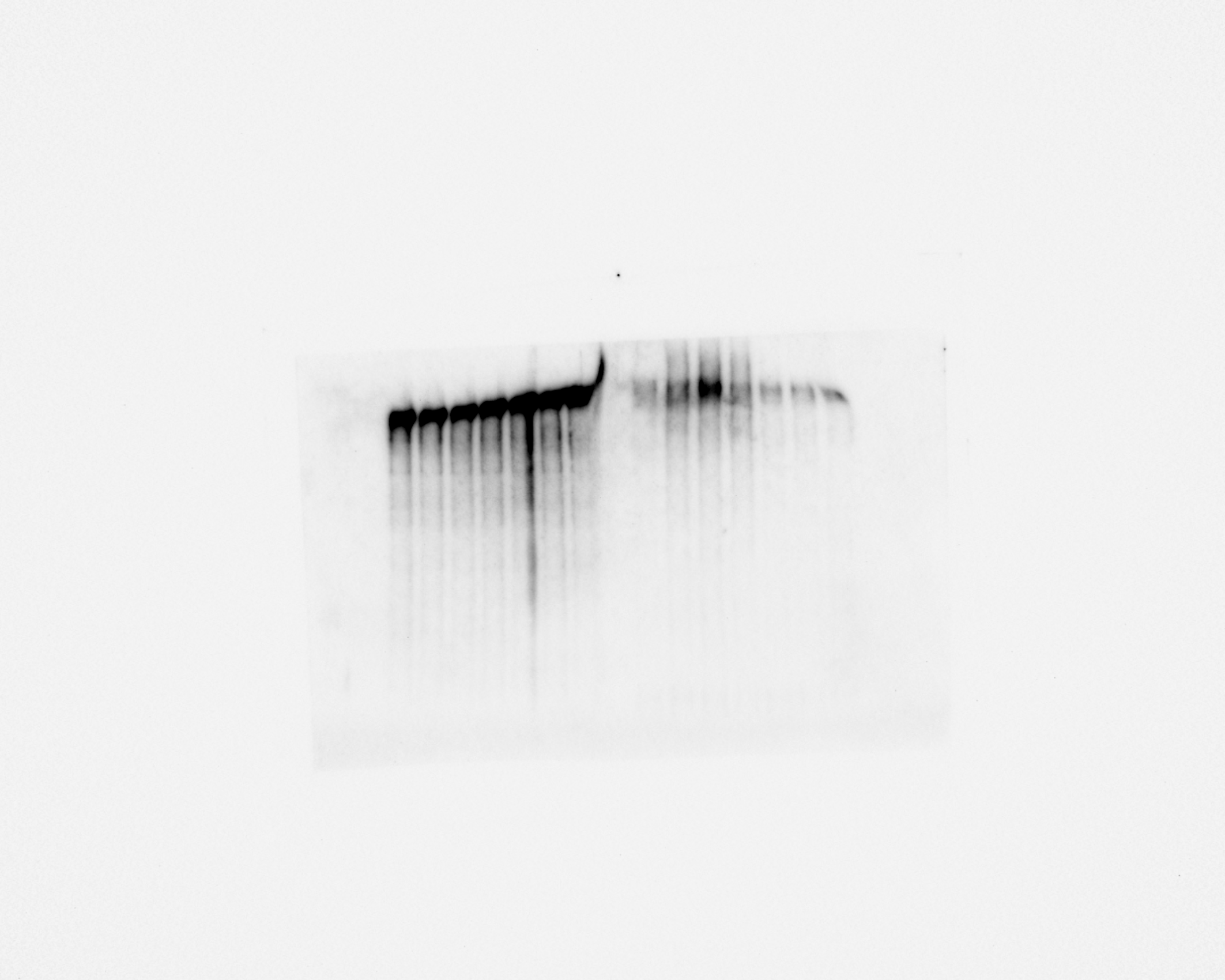

Supplement: Figure 2—source data 1. [file elife-106730-fig2-data1.zip › Figure 2ΓÇösource data 1/Figure 2D and Supplemental Figure 2B/071325-TUBE-IP_53bp1-3-8%gel_16(Chemiluminescence).tif]

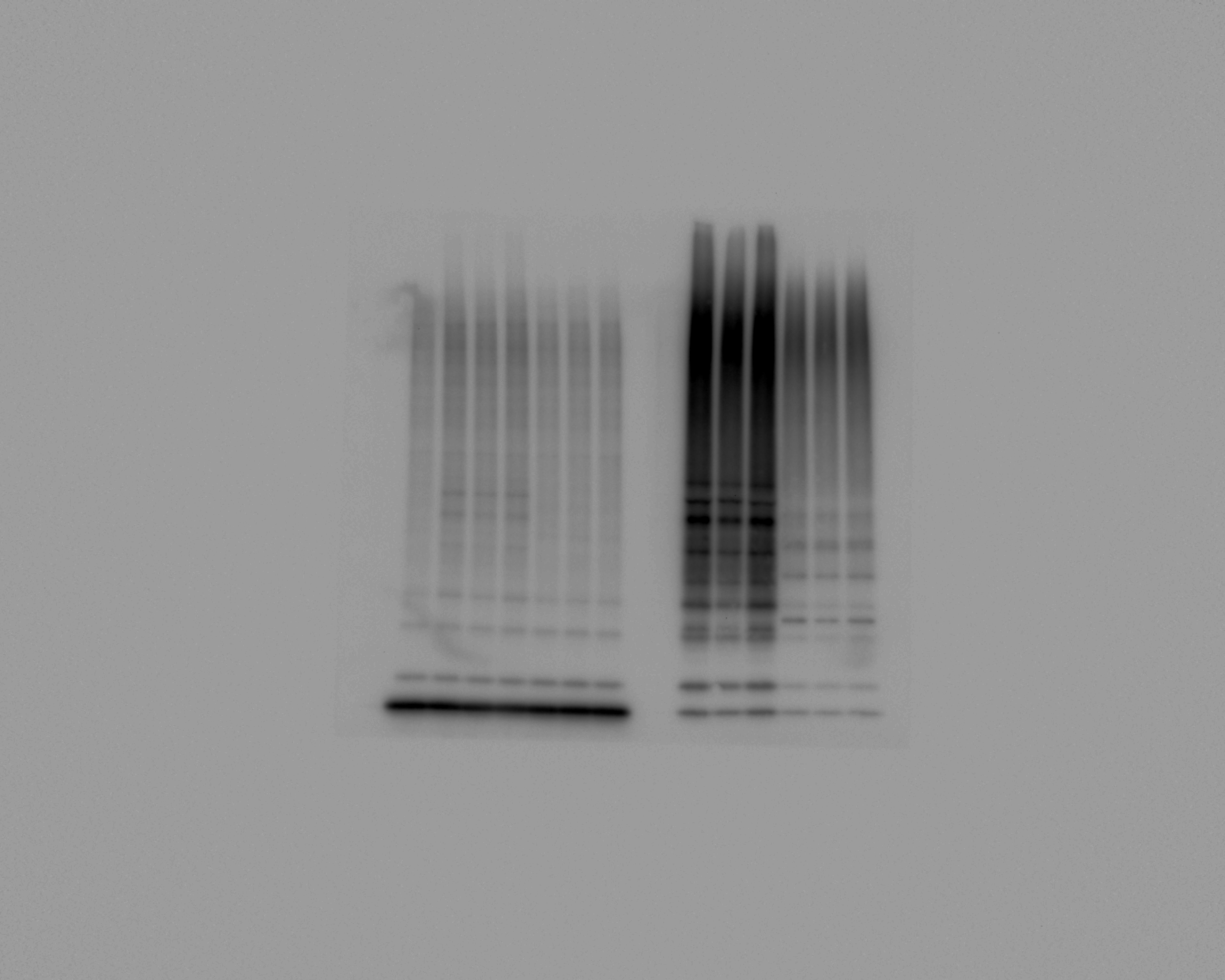

Supplement: Figure 2—source data 1. [file elife-106730-fig2-data1.zip › Figure 2ΓÇösource data 1/Figure 2D and Supplemental Figure 2B/071325-TUBE-IP_Ub_3(Chemiluminescence).tif]

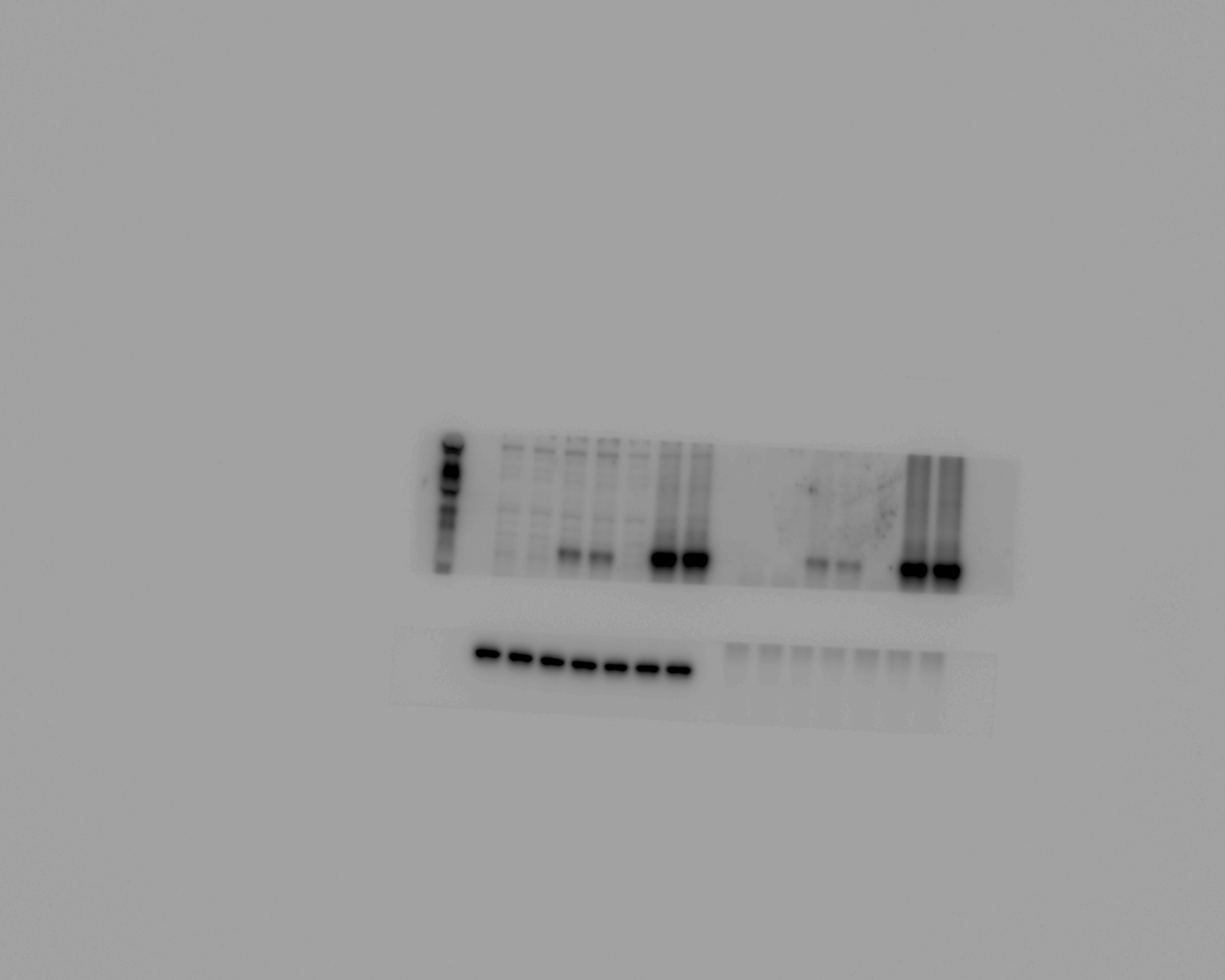

Supplement: Figure 2—source data 1. [file elife-106730-fig2-data1.zip › Figure 2ΓÇösource data 1/Figure 2D and Supplemental Figure 2B/071325-TUBE-IP_gmcl1_betaAvtin_2(Chemiluminescence).tif]

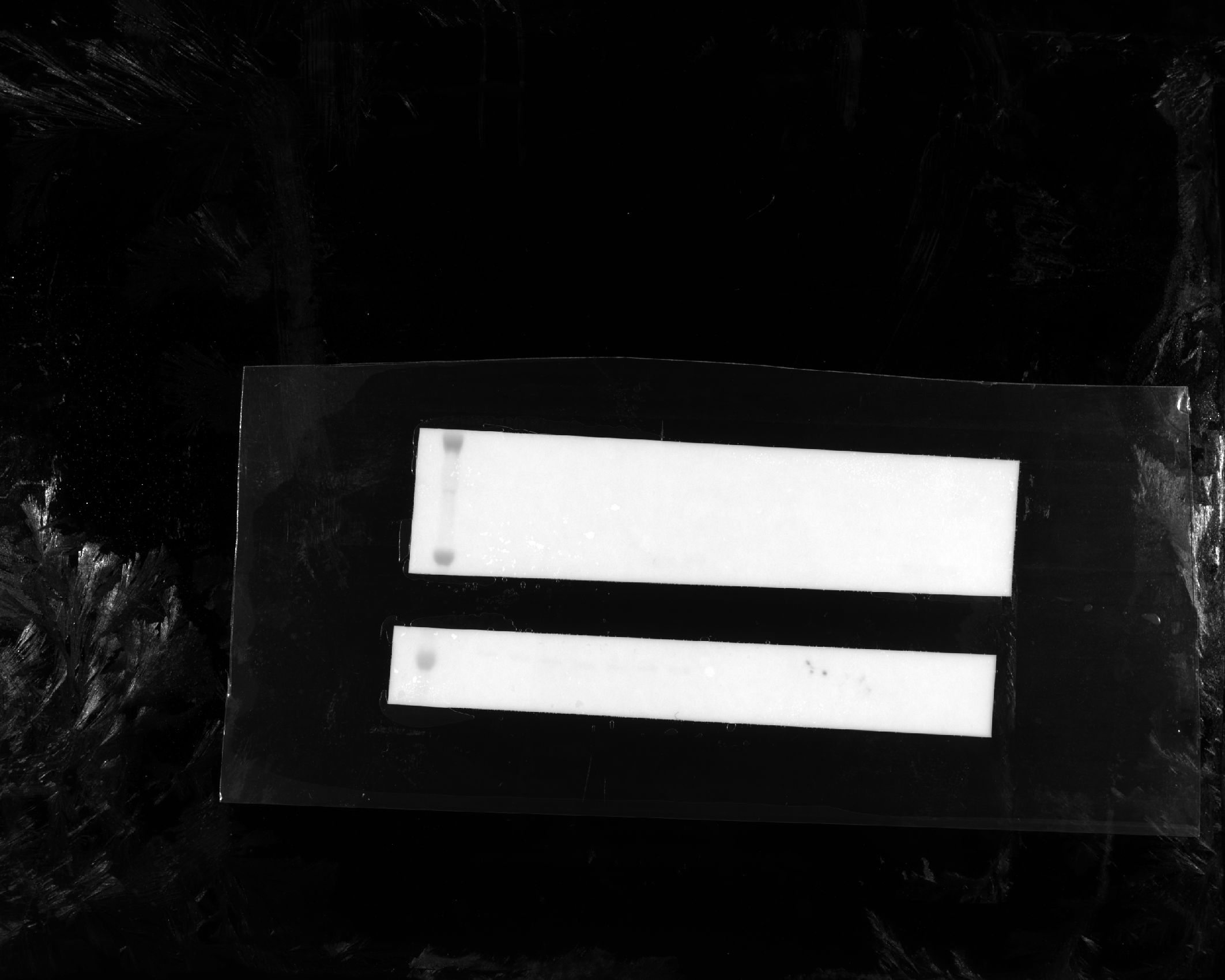

Supplement: Figure 2—source data 1. [file elife-106730-fig2-data1.zip › Figure 2ΓÇösource data 1/Figure 2D and Supplemental Figure 2B/071325-TUBE-IP_gmcl1_betaAvtin_6(Colorimetric).tif]

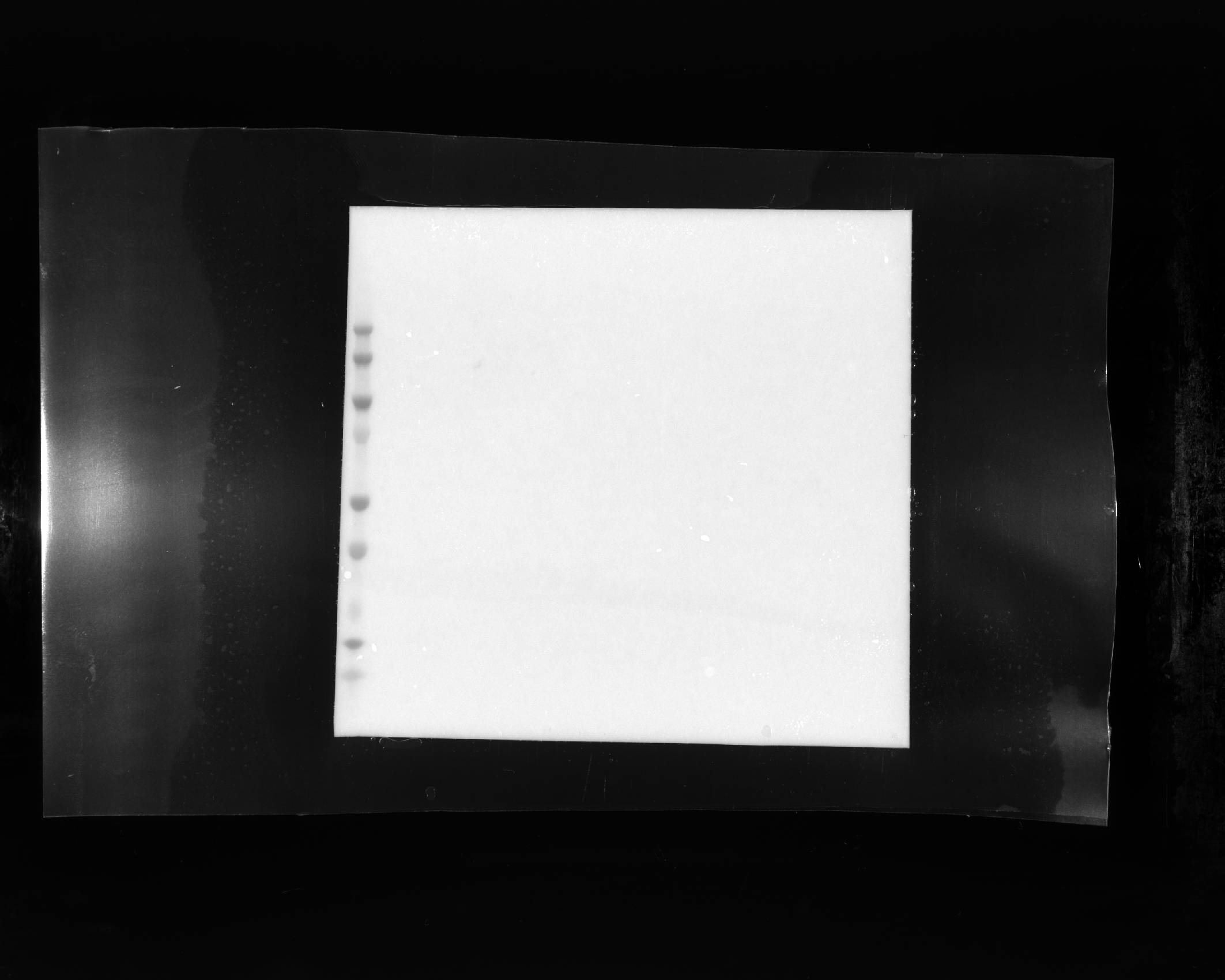

Supplement: Figure 2—source data 1. [file elife-106730-fig2-data1.zip › Figure 2ΓÇösource data 1/Figure 2D and Supplemental Figure 2B/071325-TUBE-IP_Ub_6(Colorimetric).tif]

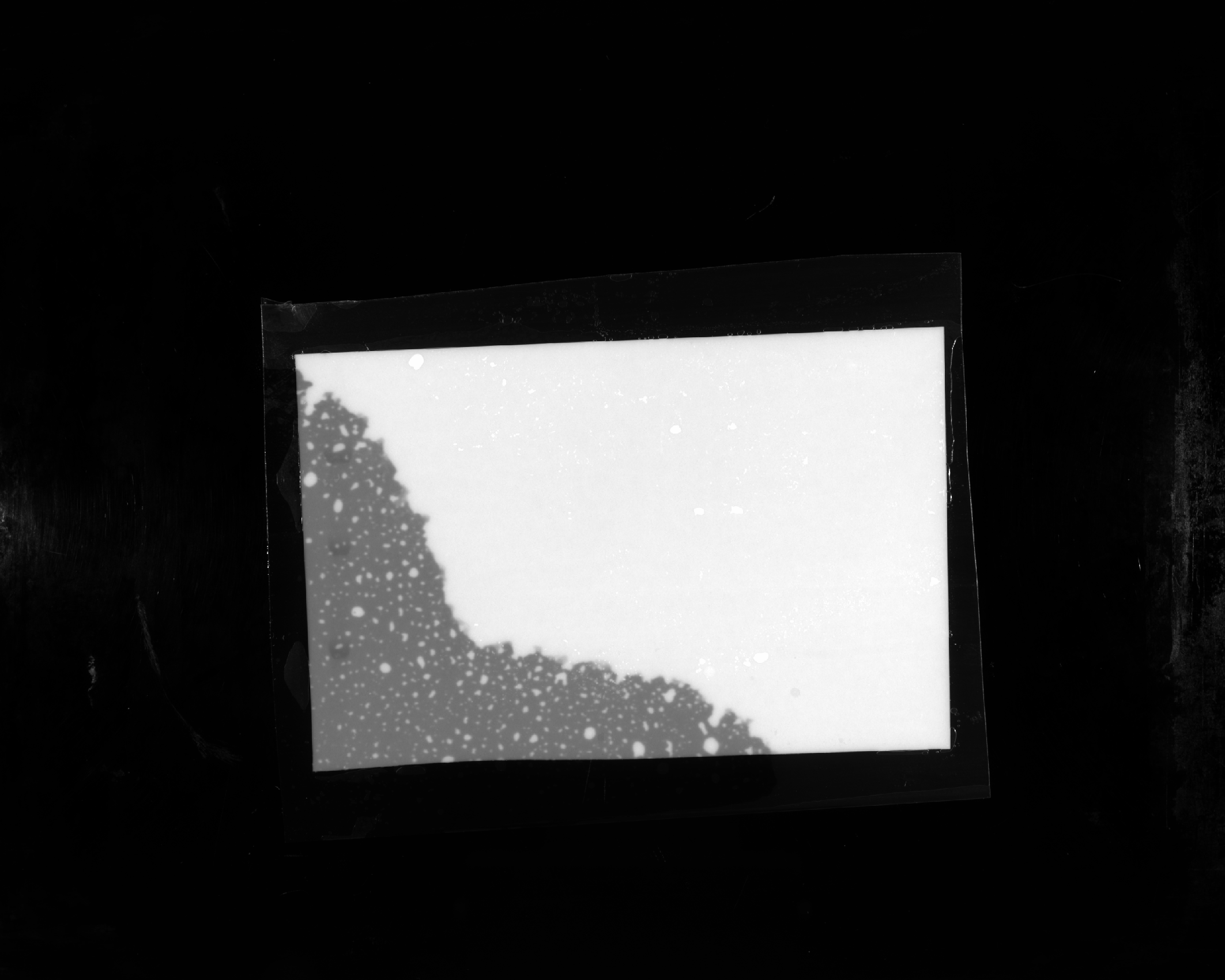

Supplement: Figure 2—source data 1. [file elife-106730-fig2-data1.zip › Figure 2ΓÇösource data 1/Figure 2D and Supplemental Figure 2B/071325-TUBE-IP_53bp1-3-8%gel_17(Colorimetric).tif]

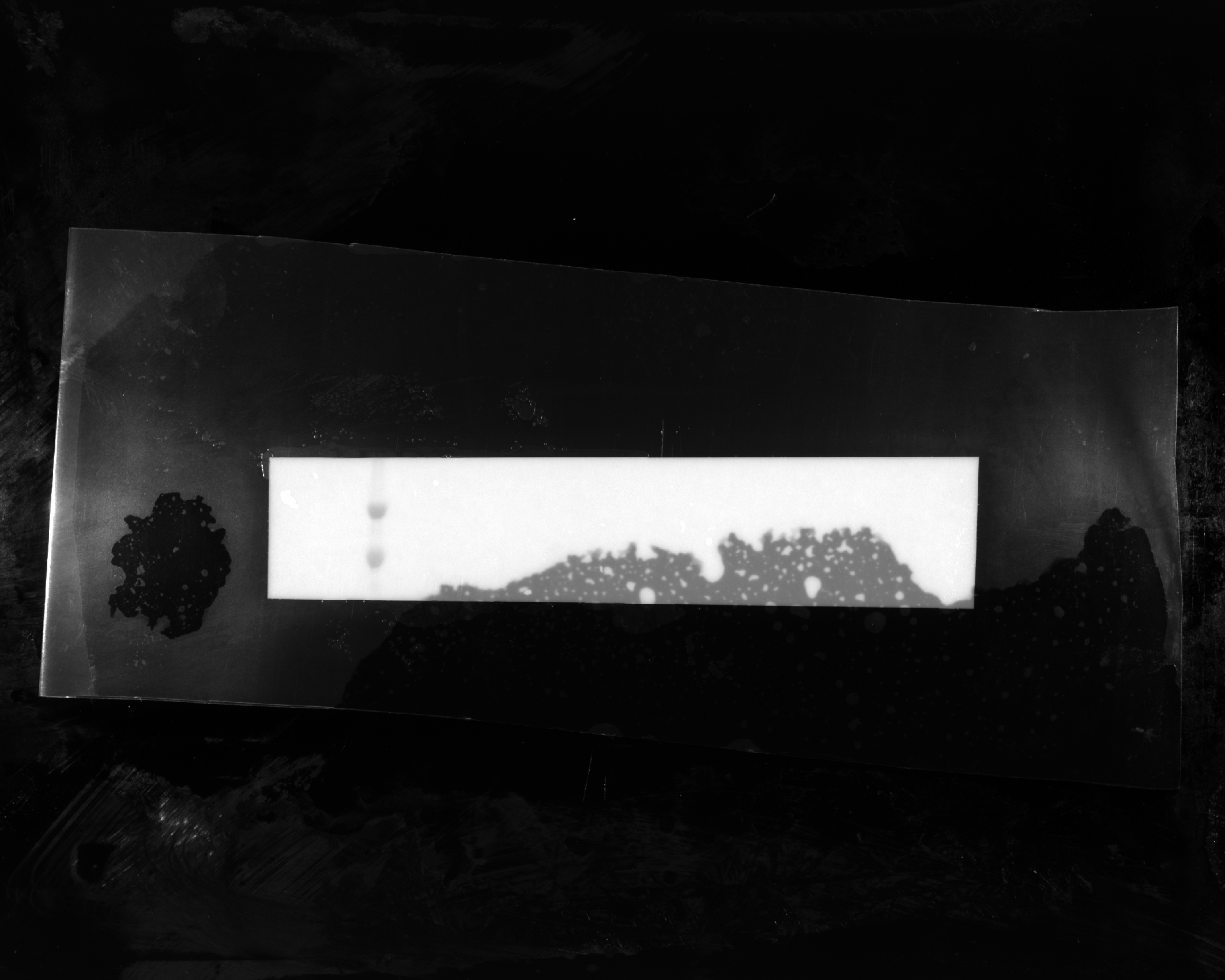

Supplement: Figure 2—source data 1. [file elife-106730-fig2-data1.zip › Figure 2ΓÇösource data 1/Figure 2D and Supplemental Figure 2B/FLAG(TUBE)_2_6(Colorimetric).tif]

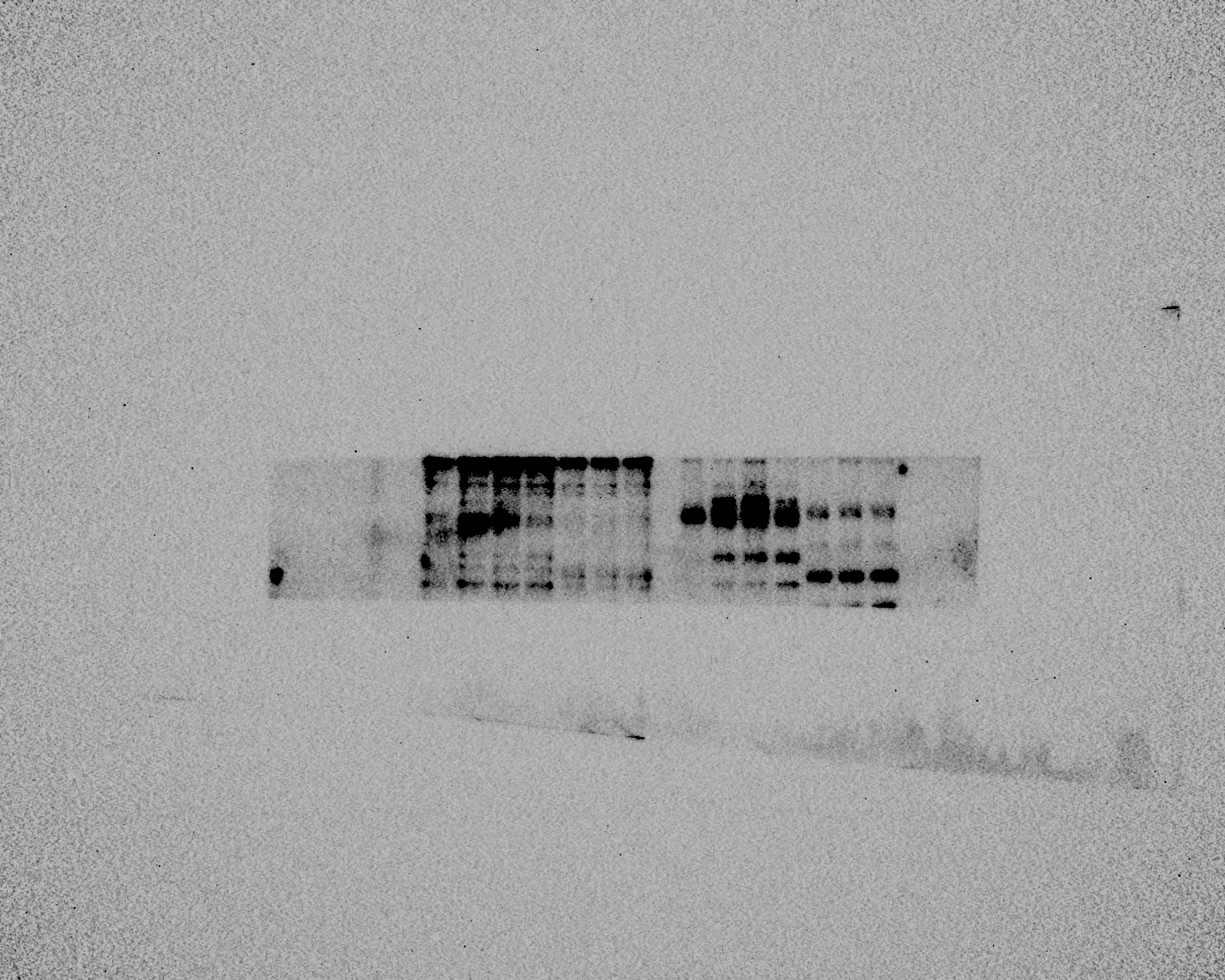

Supplement: Figure 2—source data 1. [file elife-106730-fig2-data1.zip › Figure 2ΓÇösource data 1/Figure 2D and Supplemental Figure 2B/FLAG(TUBE)_2_4(Chemiluminescence).tif]

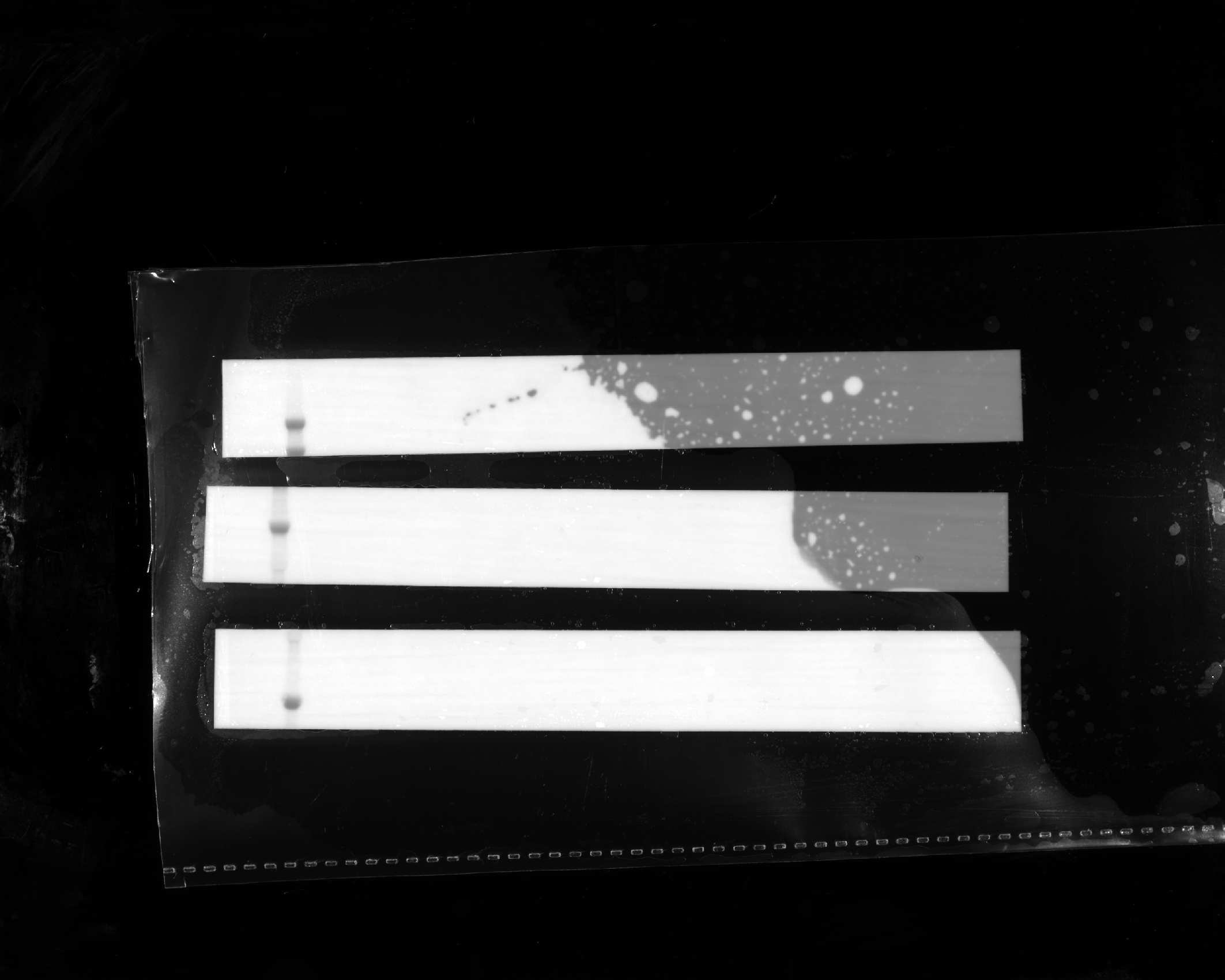

Supplement: Figure 2—source data 1. [file elife-106730-fig2-data1.zip › Figure 2ΓÇösource data 1/FIgure 2B and Supplemetal Figure 2A/122724-M_S1618_cul3_gmcl1_11(Colorimetric).tif]

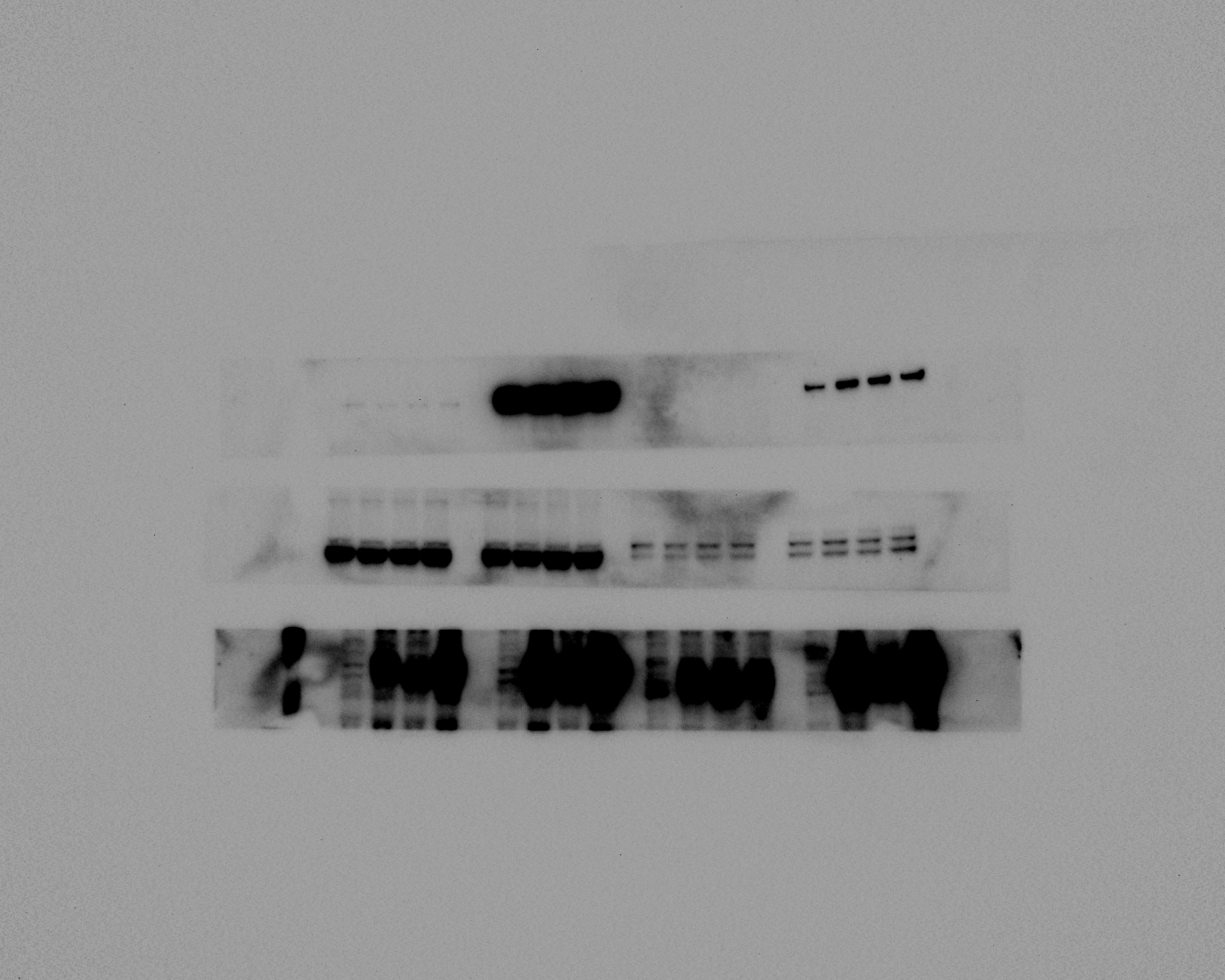

Supplement: Figure 2—source data 1. [file elife-106730-fig2-data1.zip › Figure 2ΓÇösource data 1/FIgure 2B and Supplemetal Figure 2A/122724-M_S1618_cul3_gmcl1_10(Chemiluminescence).raw16.png]

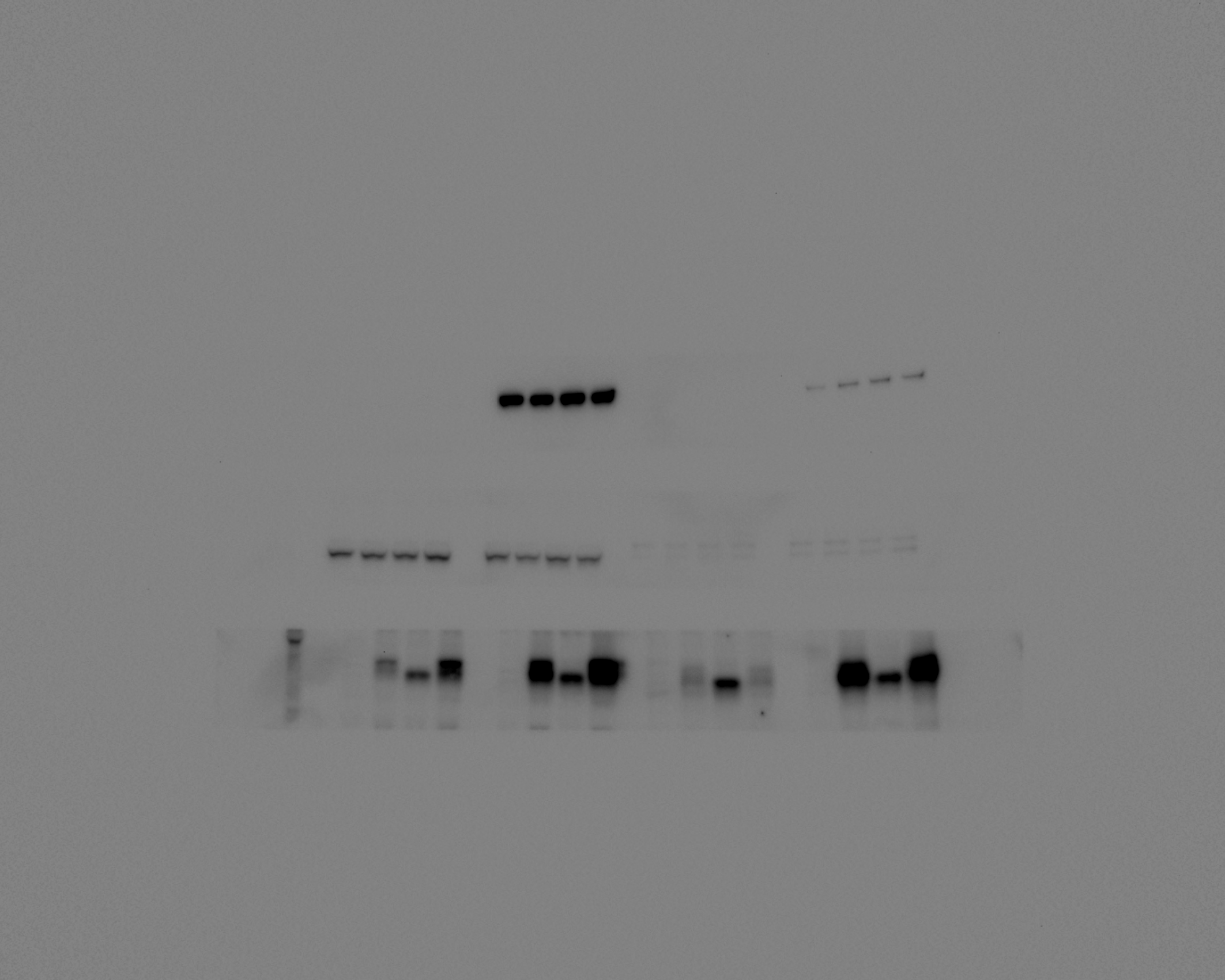

Supplement: Figure 2—source data 1. [file elife-106730-fig2-data1.zip › Figure 2ΓÇösource data 1/FIgure 2B and Supplemetal Figure 2A/122724-M_S1618_cul3_gmcl1_04(Chemiluminescence).tif]

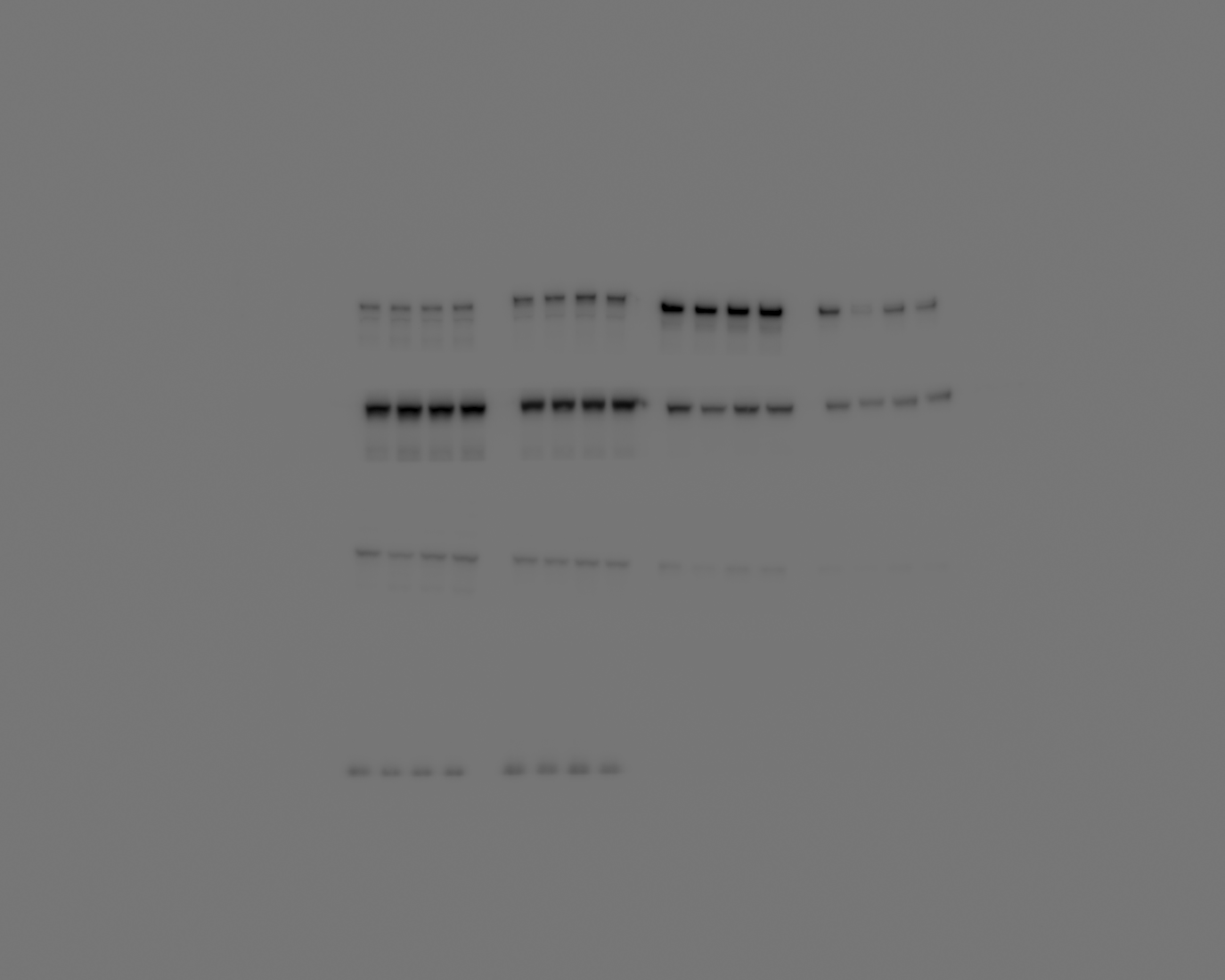

Supplement: Figure 2—source data 1. [file elife-106730-fig2-data1.zip › Figure 2ΓÇösource data 1/FIgure 2B and Supplemetal Figure 2A/122724-M_53bp1_usp28_p53_p21_2(Chemiluminescence).raw16.tif]

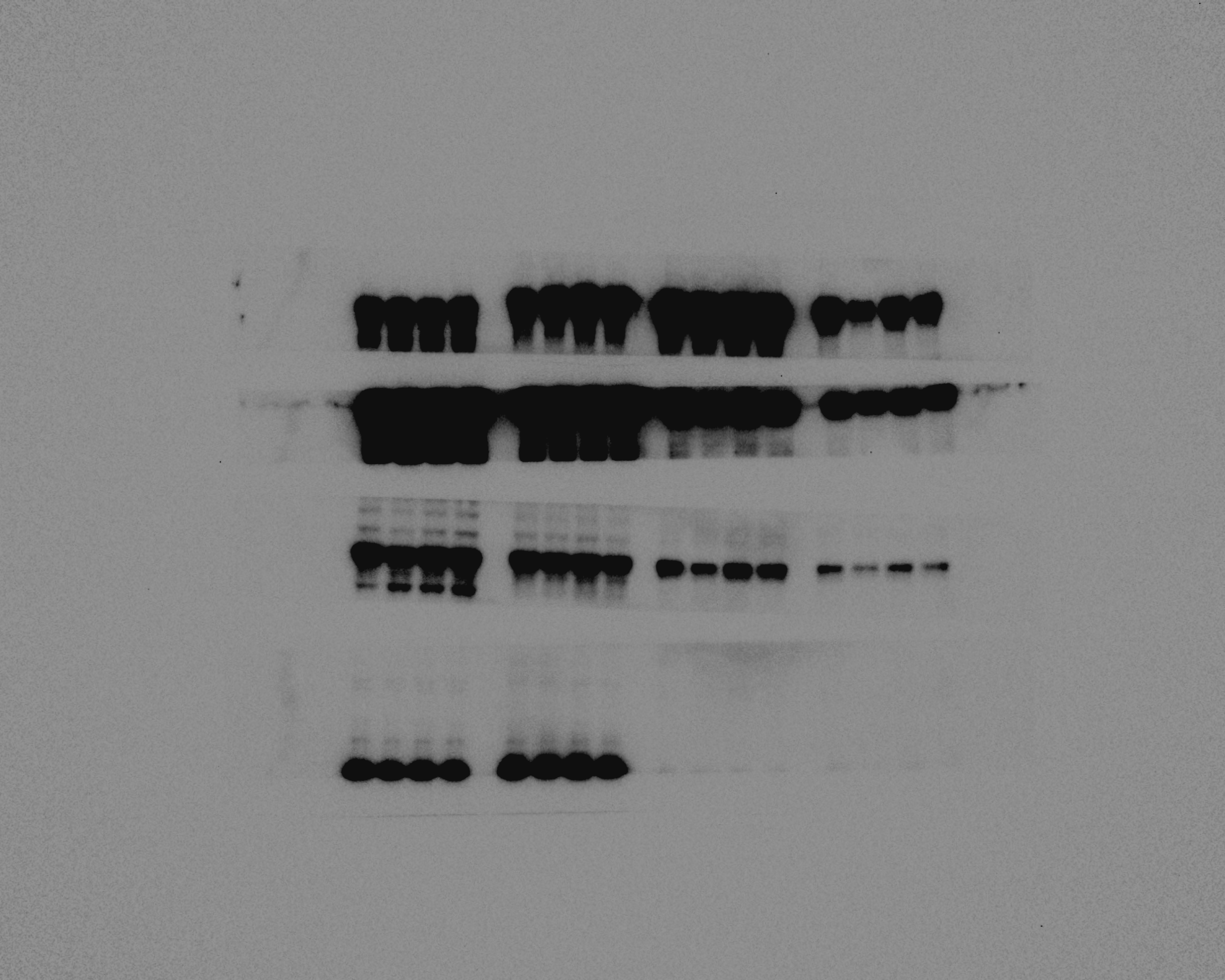

Supplement: Figure 2—source data 1. [file elife-106730-fig2-data1.zip › Figure 2ΓÇösource data 1/FIgure 2B and Supplemetal Figure 2A/122724-M_53bp1_usp28_p53_p21_5(Chemiluminescence).tif]

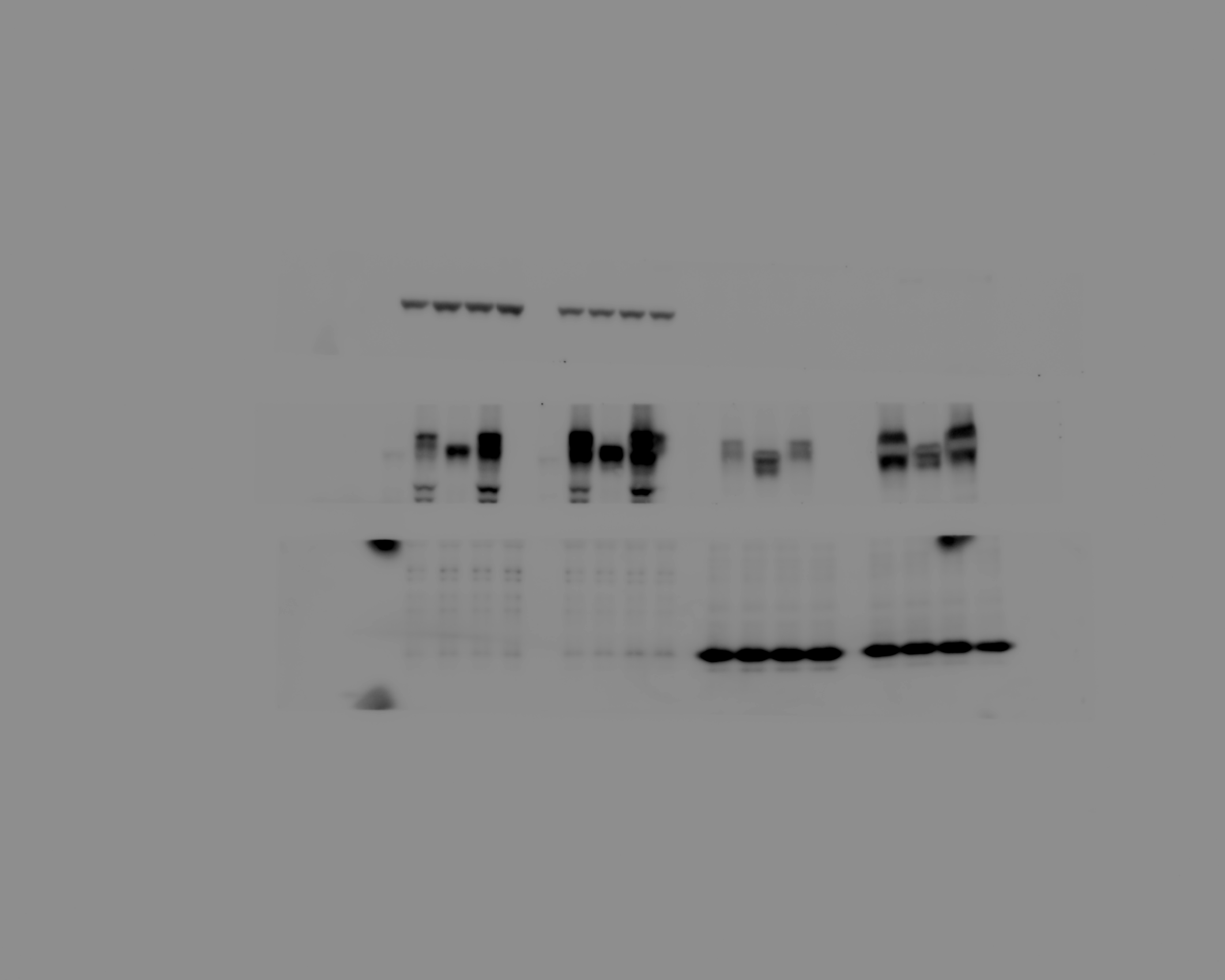

Supplement: Figure 2—source data 1. [file elife-106730-fig2-data1.zip › Figure 2ΓÇösource data 1/FIgure 2B and Supplemetal Figure 2A/122824-M_tubulin_Flag(Ms)_H3_4(Chemiluminescence).tif]

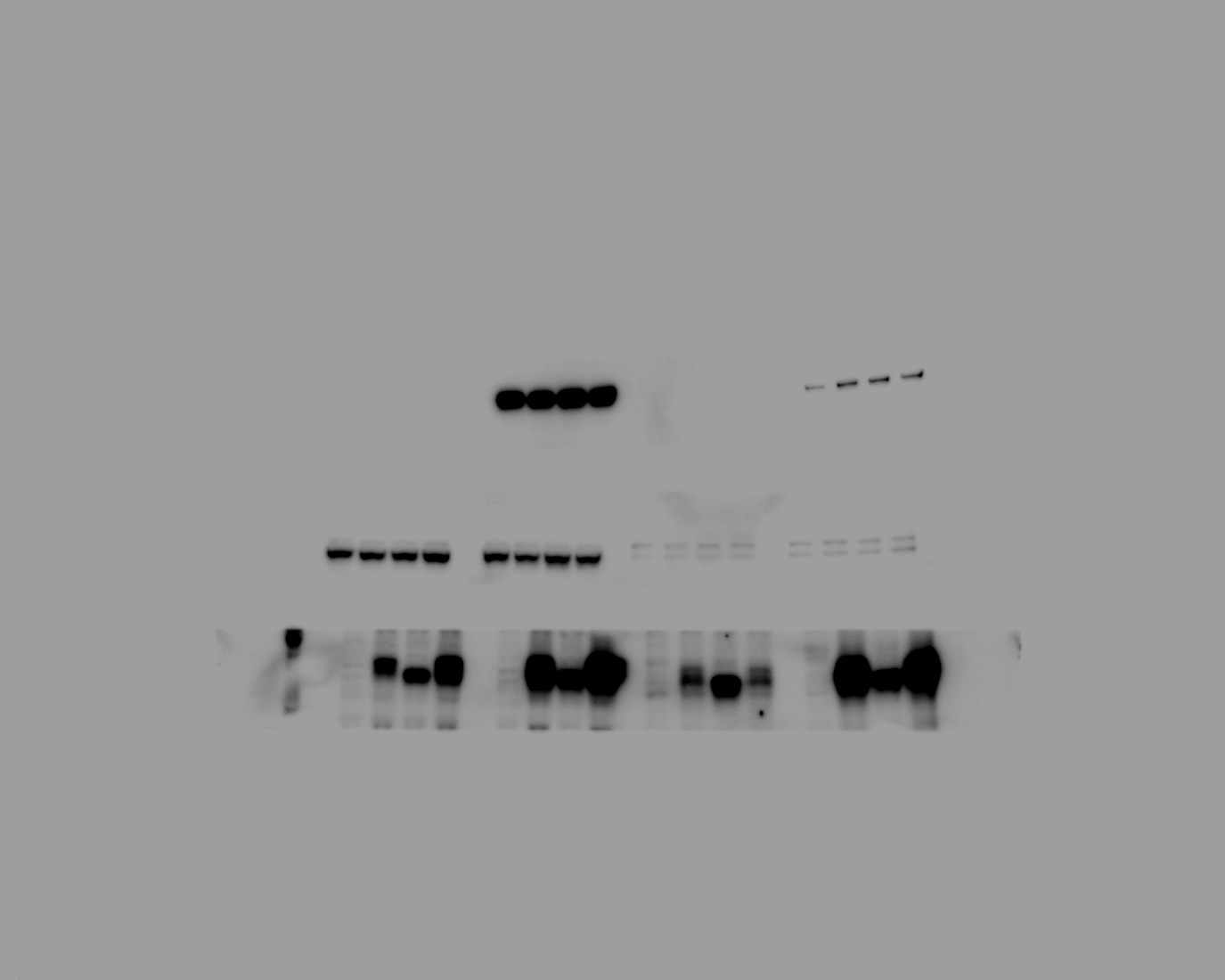

Supplement: Figure 2—source data 1. [file elife-106730-fig2-data1.zip › Figure 2ΓÇösource data 1/FIgure 2B and Supplemetal Figure 2A/122724-M_S1618_cul3_gmcl1_10(Chemiluminescence)(GMCL1).raw16.png]

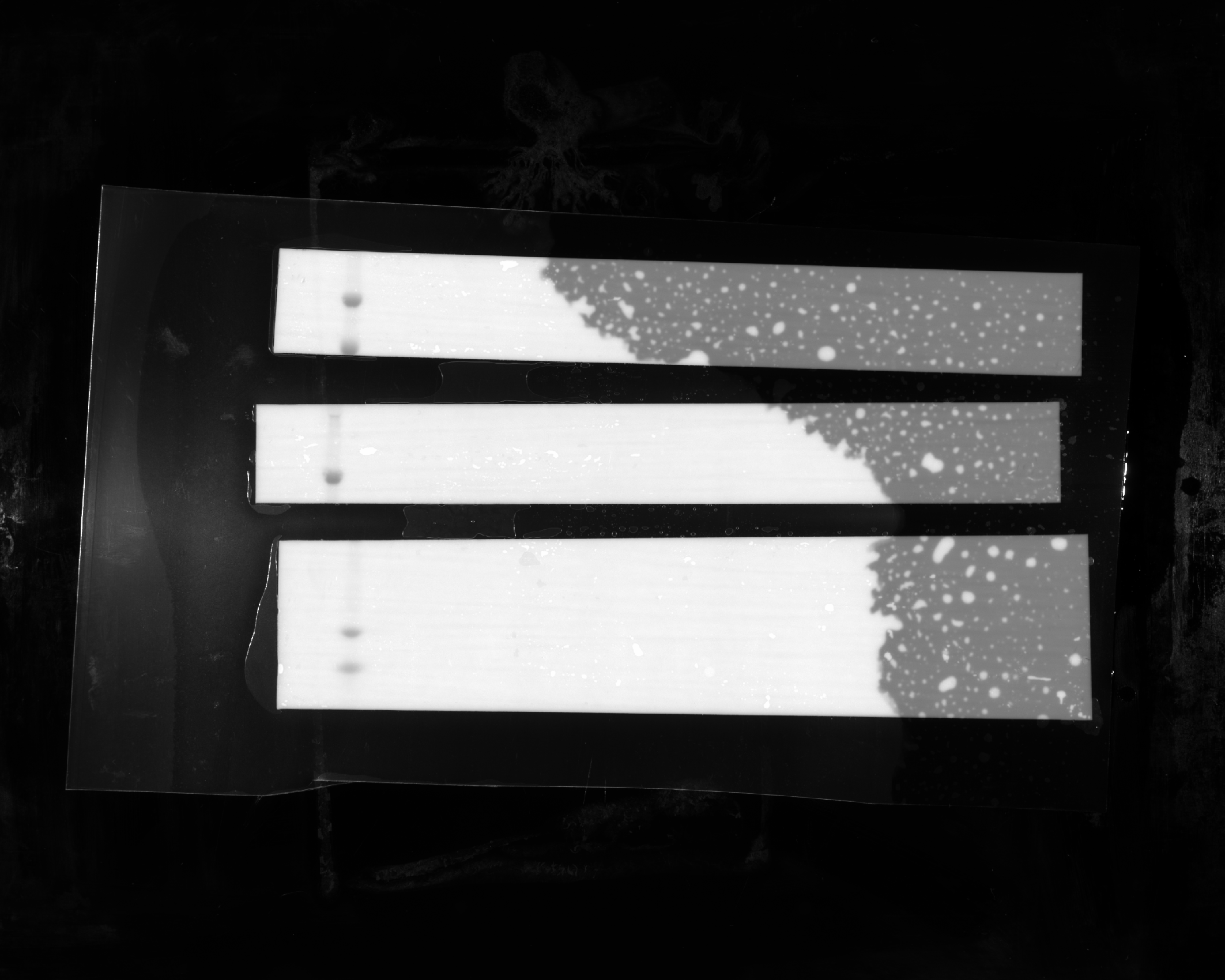

Supplement: Figure 2—source data 1. [file elife-106730-fig2-data1.zip › Figure 2ΓÇösource data 1/FIgure 2B and Supplemetal Figure 2A/122824-M_tubulin_Flag(Ms)_H3_6(Colorimetric).tif]

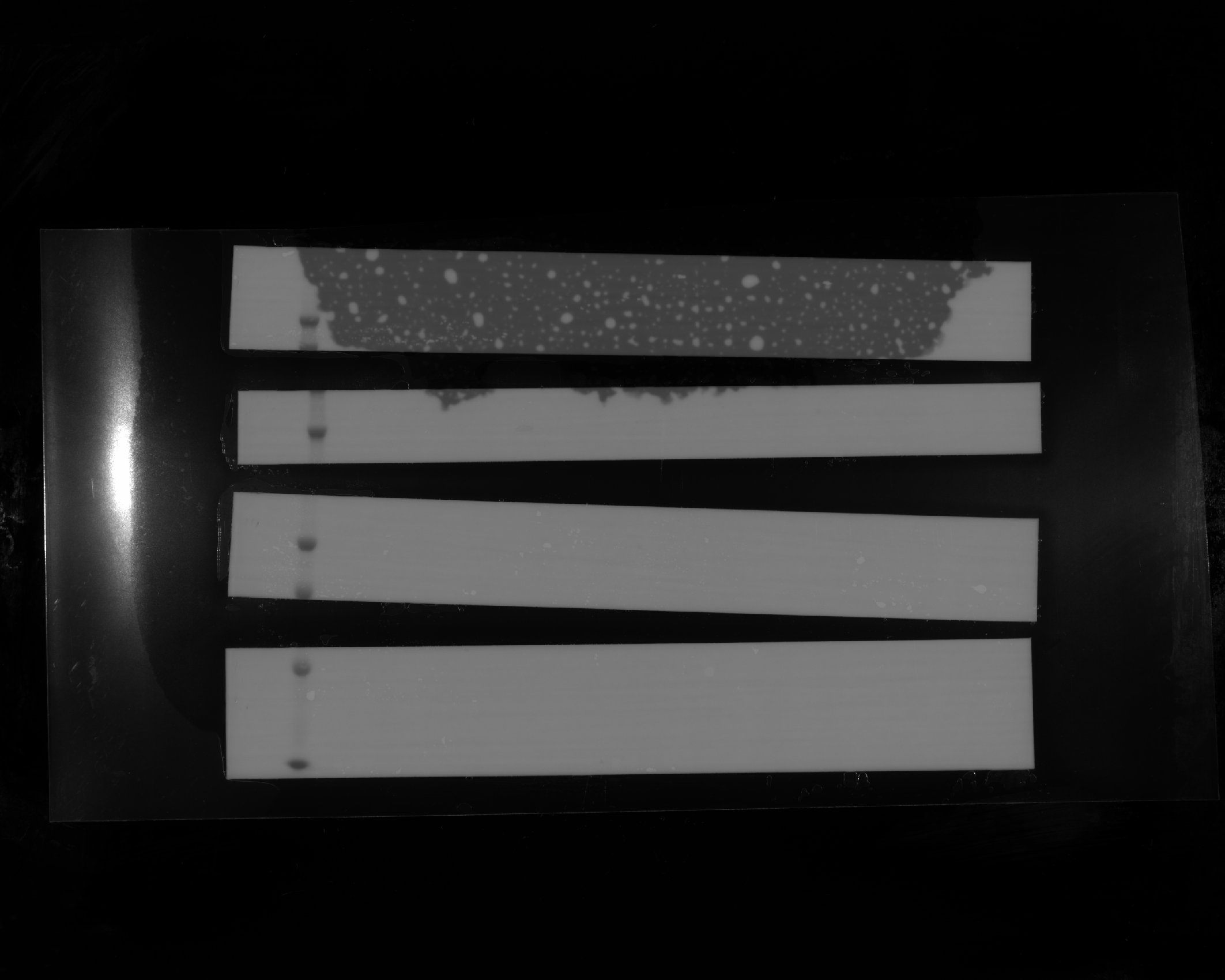

Supplement: Figure 2—source data 1. [file elife-106730-fig2-data1.zip › Figure 2ΓÇösource data 1/FIgure 2B and Supplemetal Figure 2A/122724-M_53bp1_usp28_p53_p21_6(Colorimetric).tif]

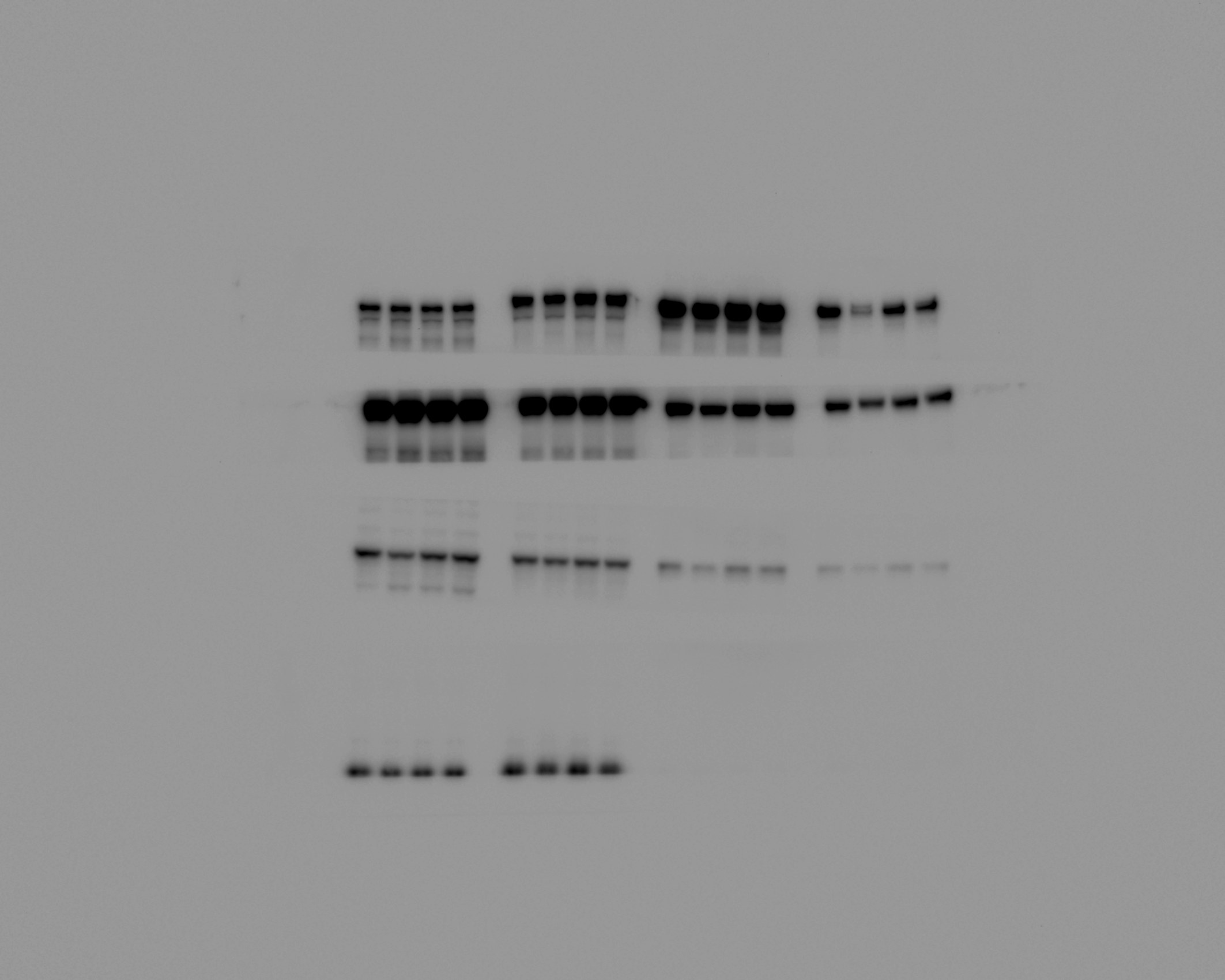

Supplement: Figure 2—source data 1. [file elife-106730-fig2-data1.zip › Figure 2ΓÇösource data 1/FIgure 2B and Supplemetal Figure 2A/122724-M_53bp1_usp28_p53_p21_3(Chemiluminescence).tif]

Figure2B and Supplemental Figure2A

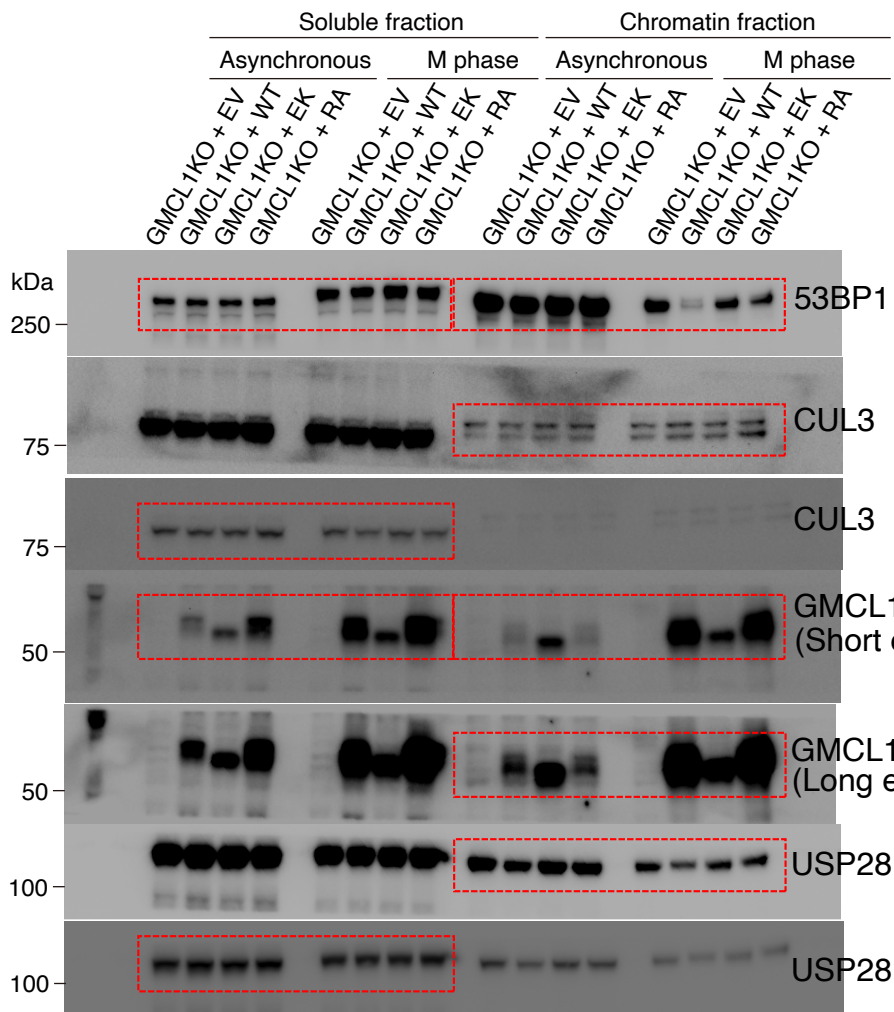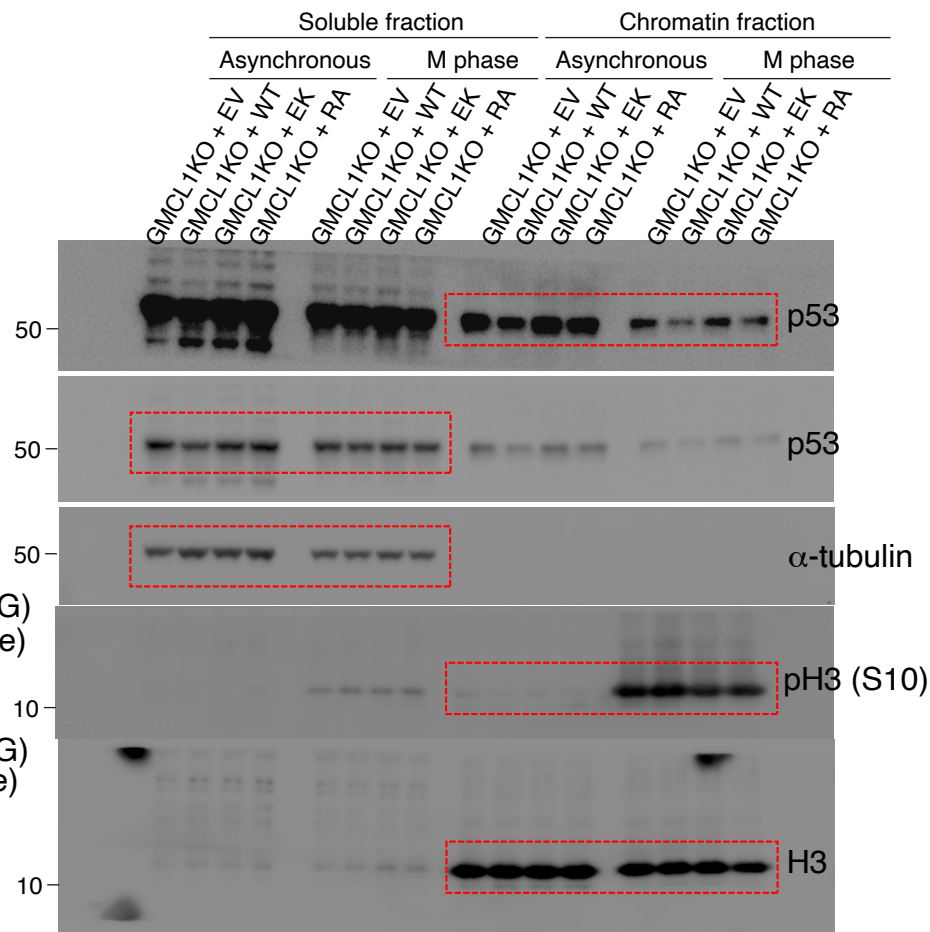

Supplement: Figure 2—source data 2. [file elife-106730-fig2-data2.zip › Figure 2ΓÇösource data 2/Figure2-2_Raw uncropped supporting Western blot files.pdf]

Figure2C

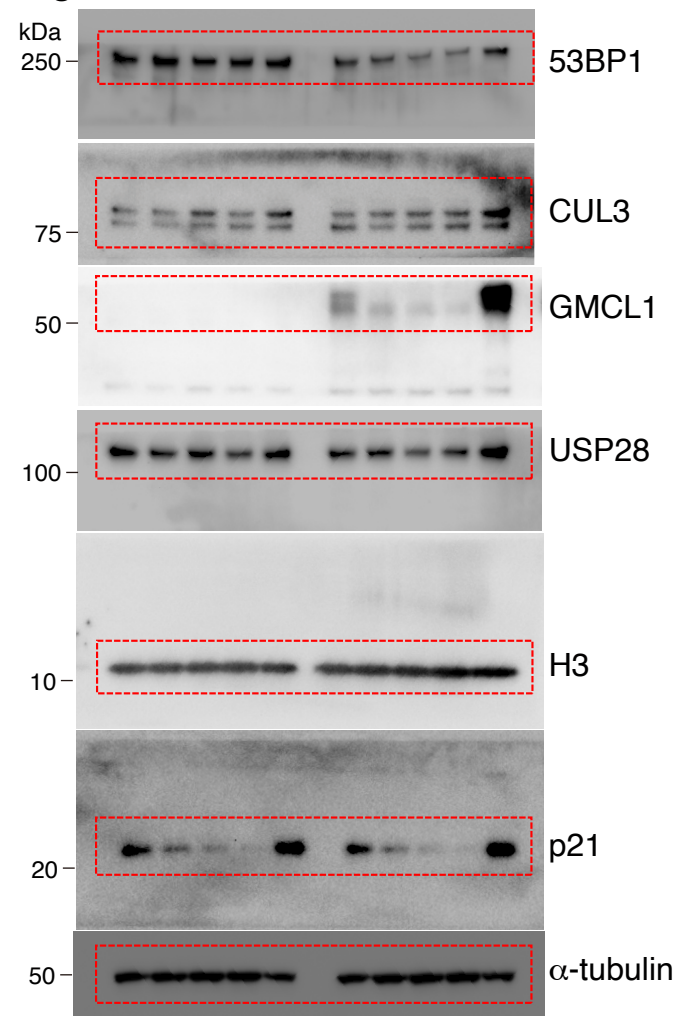

Figure2D

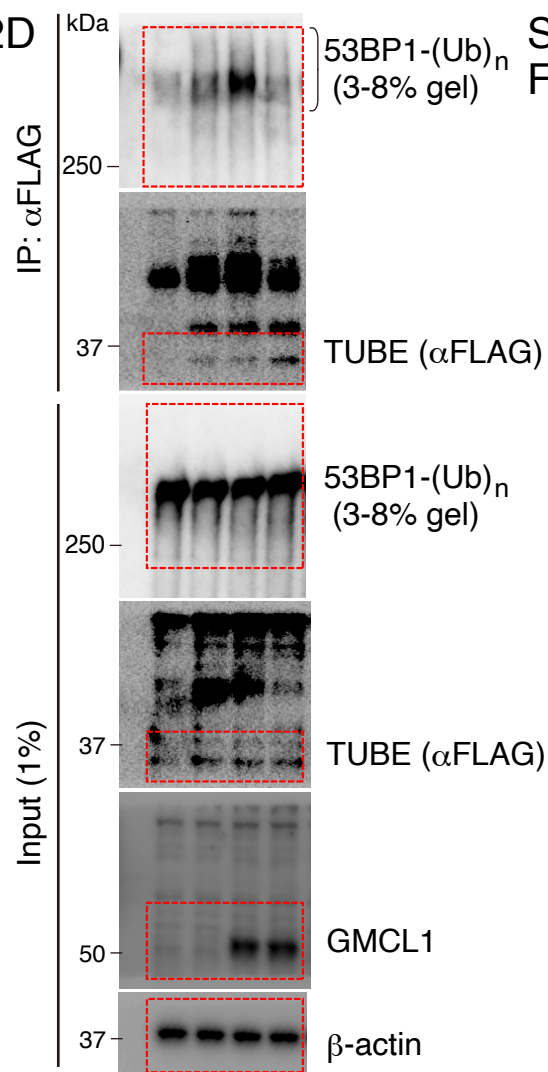

Supplemental Figure2B

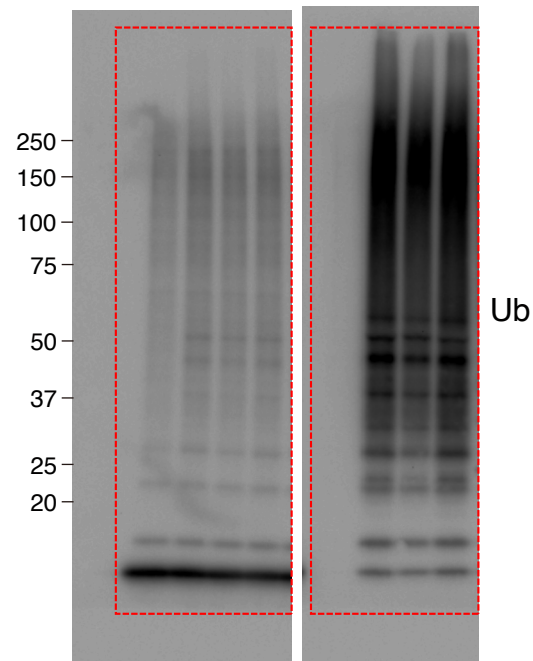

Supplement: Figure 2—source data 2. [file elife-106730-fig2-data2.zip › Figure 2ΓÇösource data 2/Figure2-3_Raw uncropped supporting Western blot files.pdf]

Figure2A

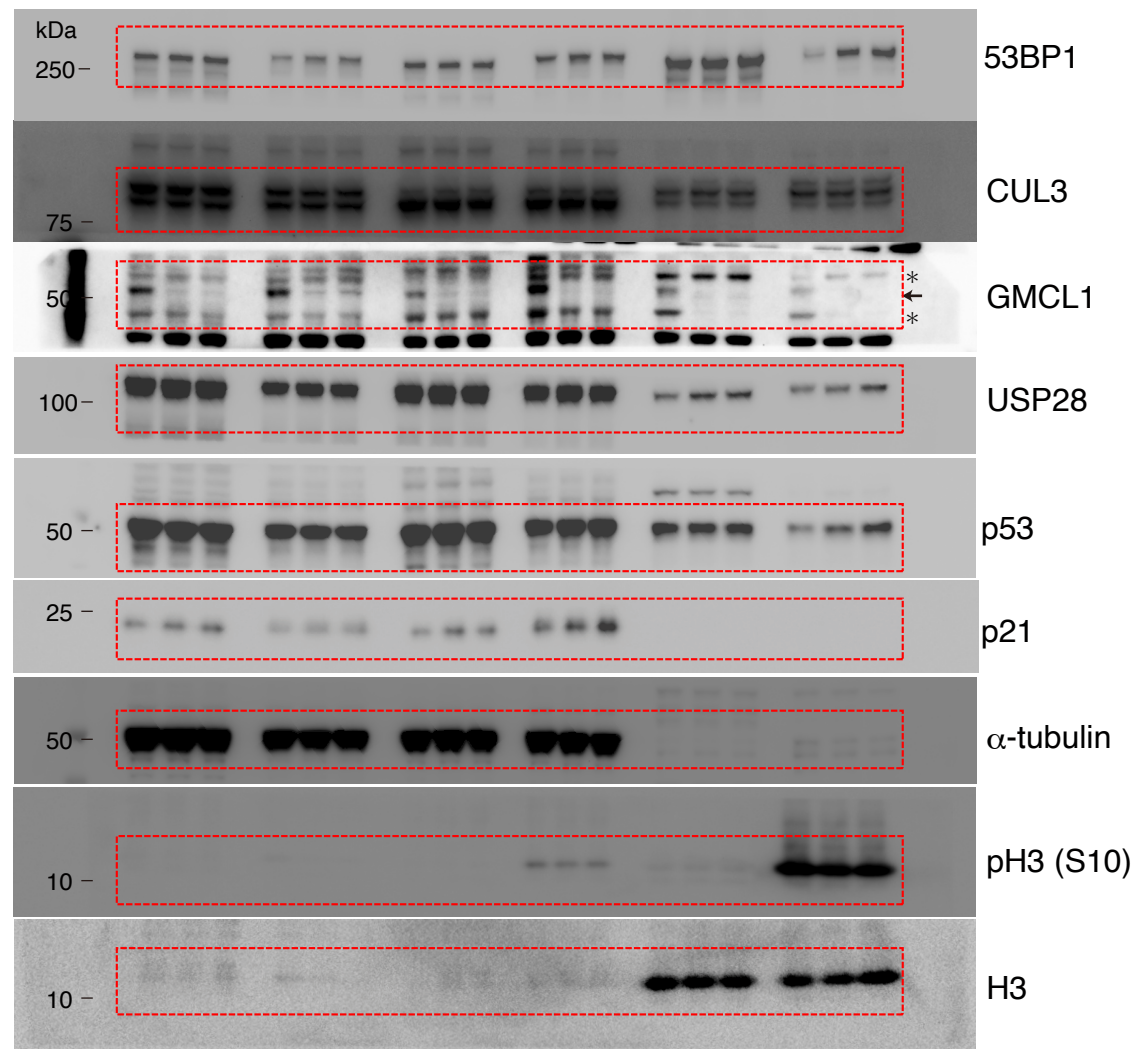

Supplement: Figure 2—source data 2. [file elife-106730-fig2-data2.zip › Figure 2ΓÇösource data 2/Figure2-1_Raw uncropped supporting Western blot files.pdf]

Figure2C (53BP1)

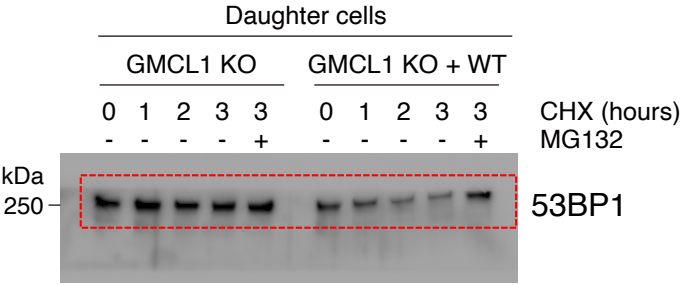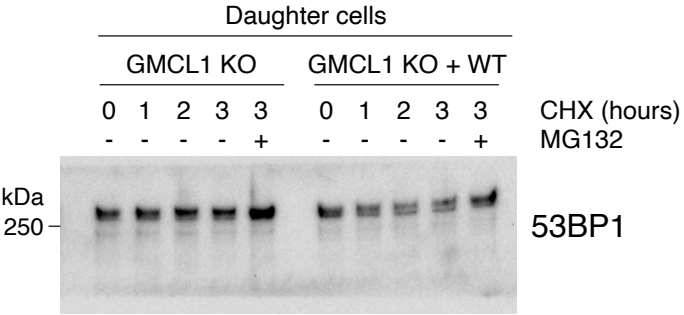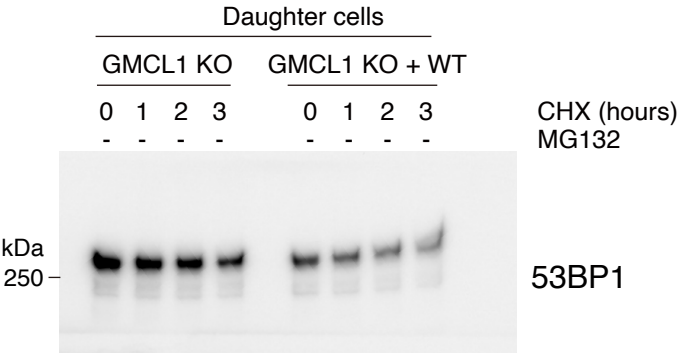

Chromatin fraction

Supplement: Figure 2—source data 2. [file elife-106730-fig2-data2.zip › Figure 2ΓÇösource data 2/Figure2C(53BP1)_Raw uncropped supporting Western blot files.pdf]

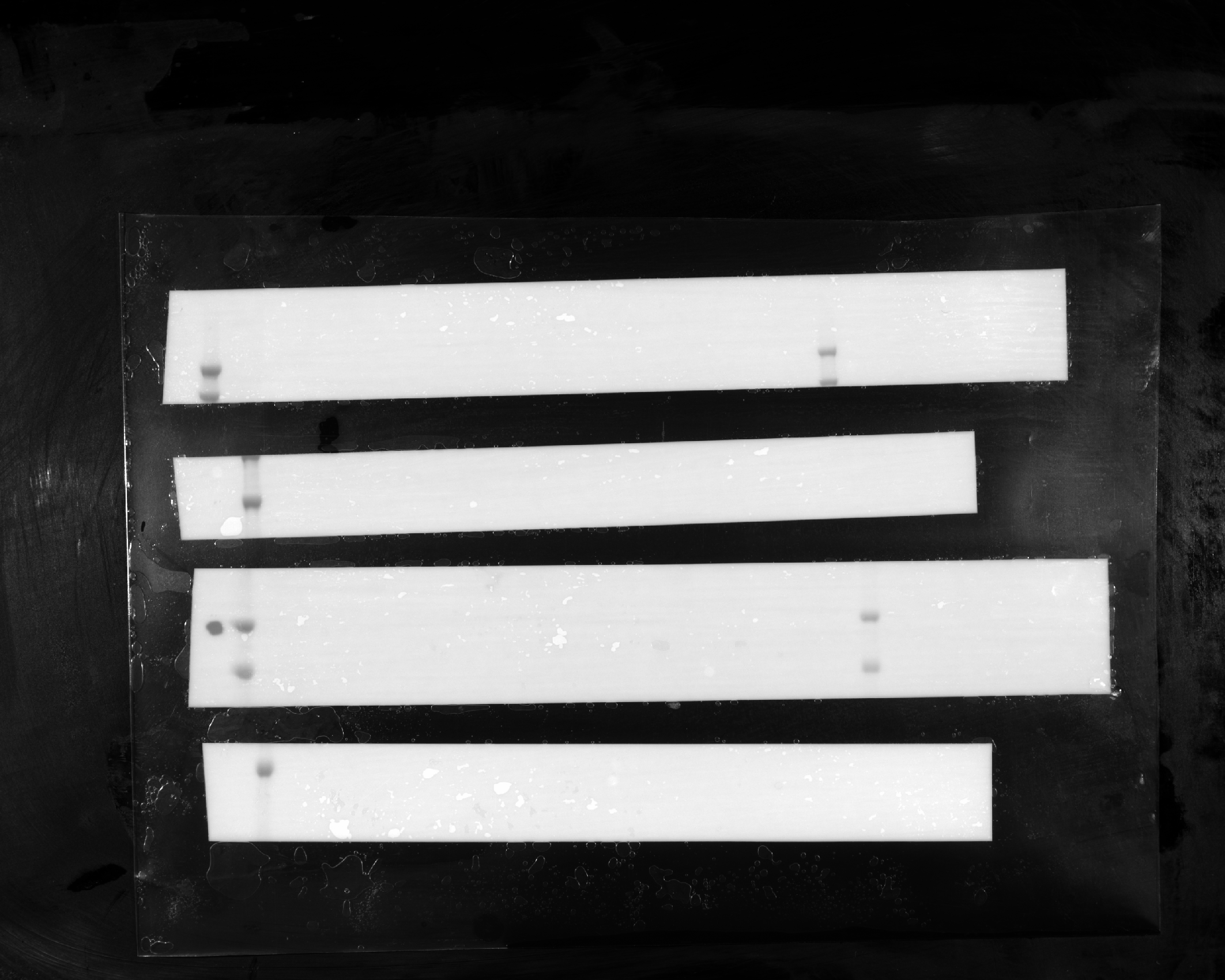

Supplement: Figure 2—figure supplement 1—source data 1. [file elife-106730-fig2-figsupp1-data1.zip › Figure 2ΓÇöfigure supplement 1ΓÇösource data 1/Figure 2ΓÇöfigure supplement 1C/111624-G1_53bp1_usp28_FLAG_CCND1_6(Colorimetric).tif]

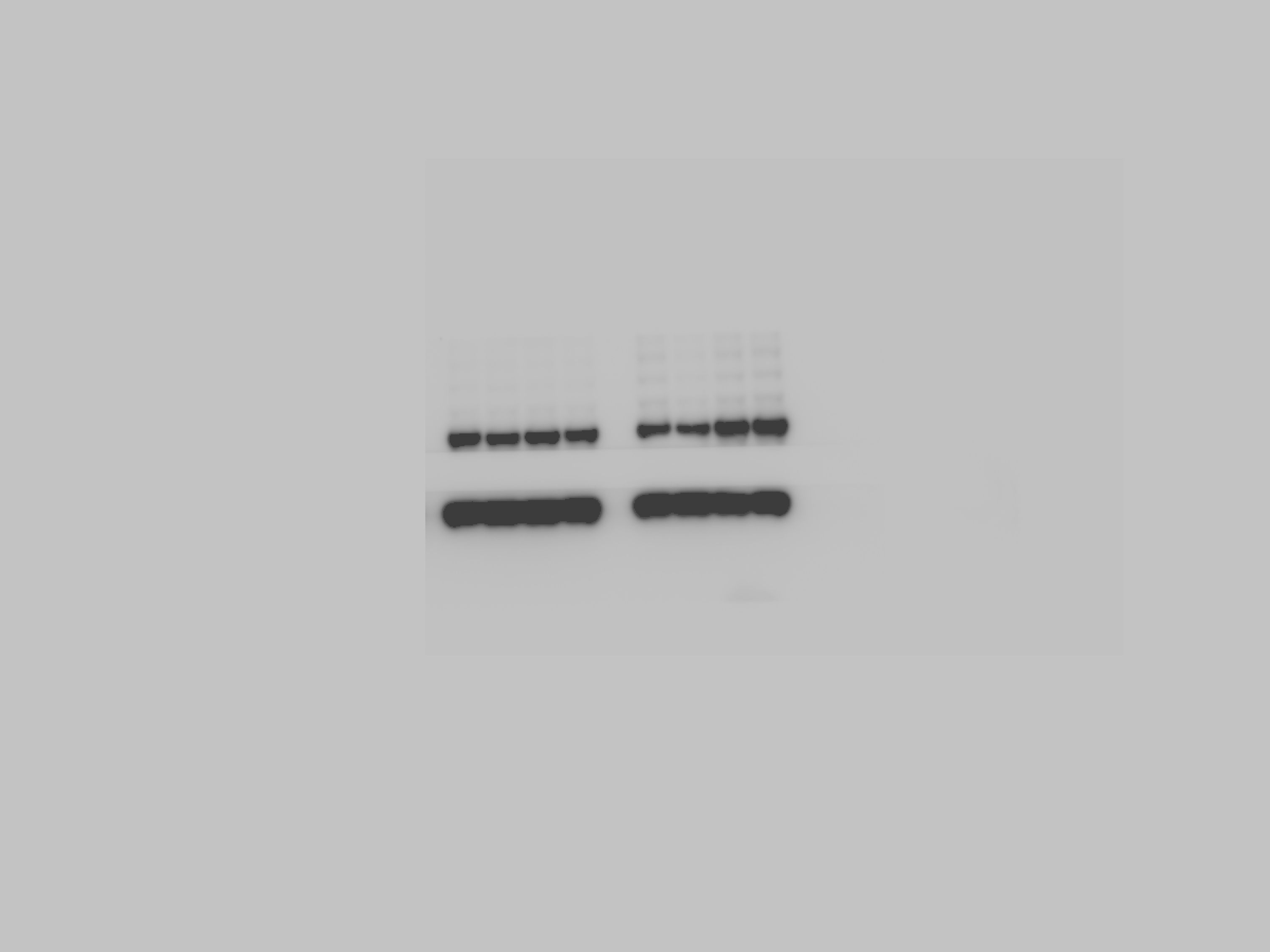

Supplement: Figure 2—figure supplement 1—source data 1. [file elife-106730-fig2-figsupp1-data1.zip › Figure 2ΓÇöfigure supplement 1ΓÇösource data 1/Figure 2ΓÇöfigure supplement 1C/111624_p53_tubulin_Background.tif]

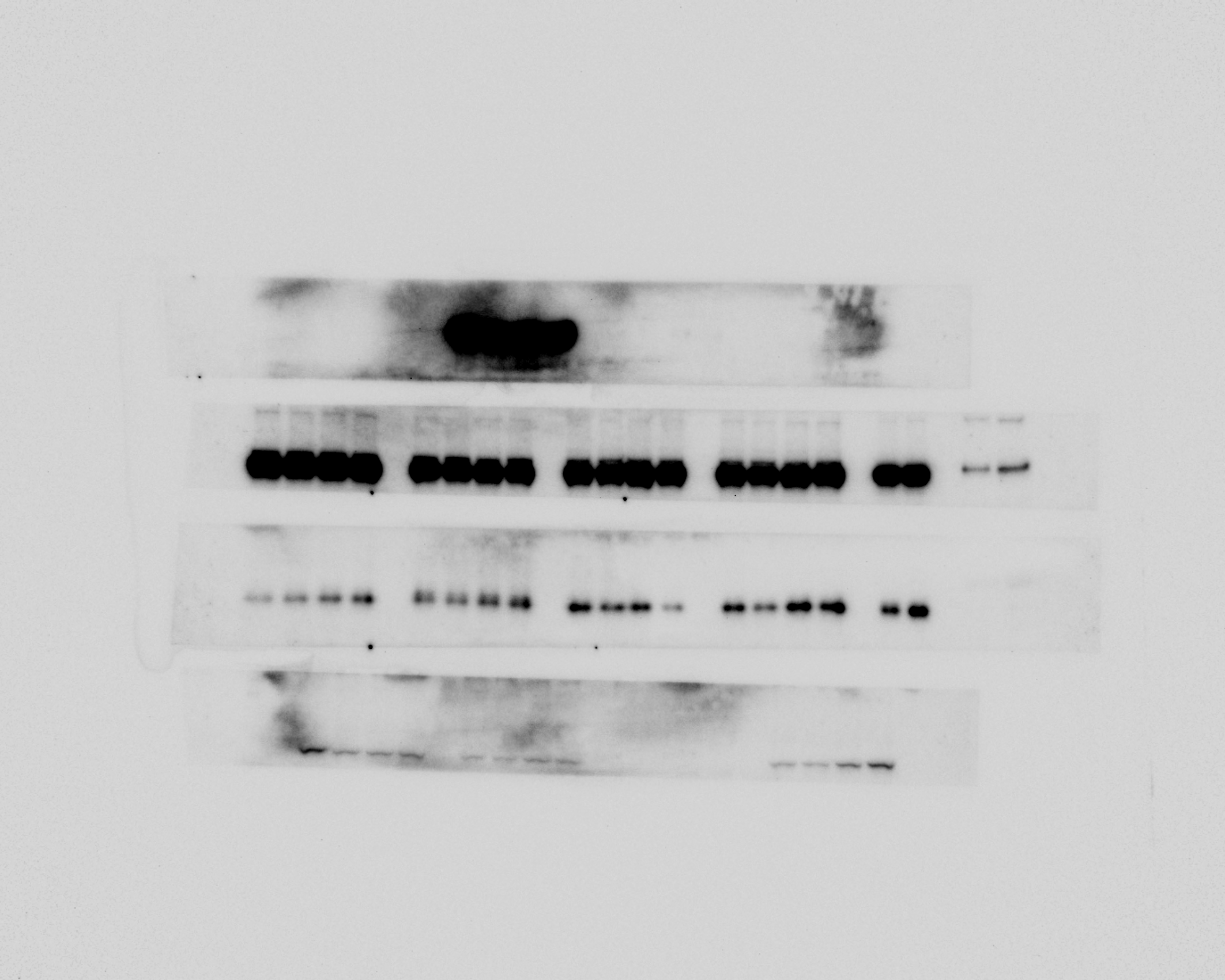

Supplement: Figure 2—figure supplement 1—source data 1. [file elife-106730-fig2-figsupp1-data1.zip › Figure 2ΓÇöfigure supplement 1ΓÇösource data 1/Figure 2ΓÇöfigure supplement 1C/111624-G1_s1618_cul3_p21_Ccna1_07(Chemiluminescence).tif]

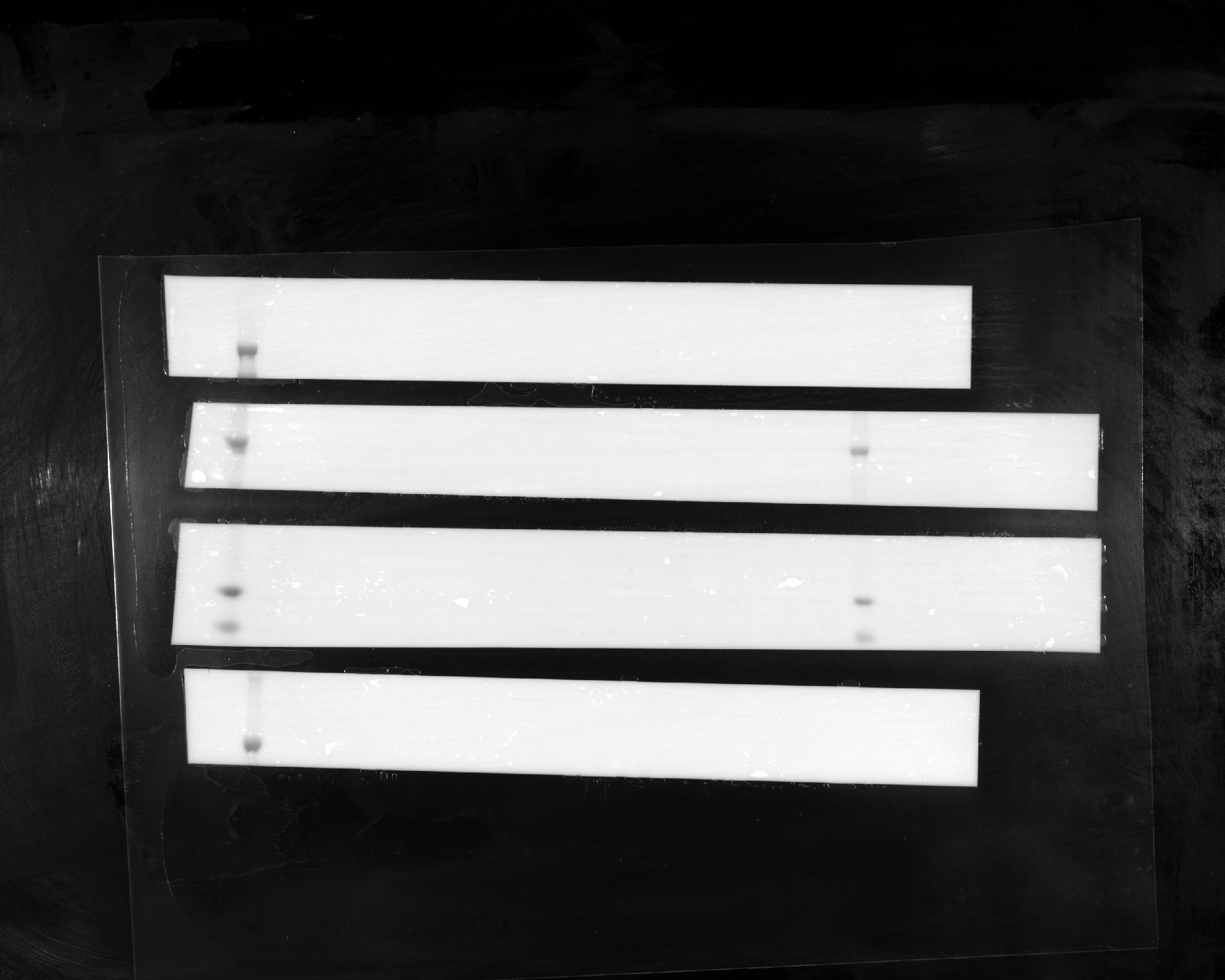

Supplement: Figure 2—figure supplement 1—source data 1. [file elife-106730-fig2-figsupp1-data1.zip › Figure 2ΓÇöfigure supplement 1ΓÇösource data 1/Figure 2ΓÇöfigure supplement 1C/111624-G1_s1618_cul3_p21_Ccna1_11(Colorimetric).tif]

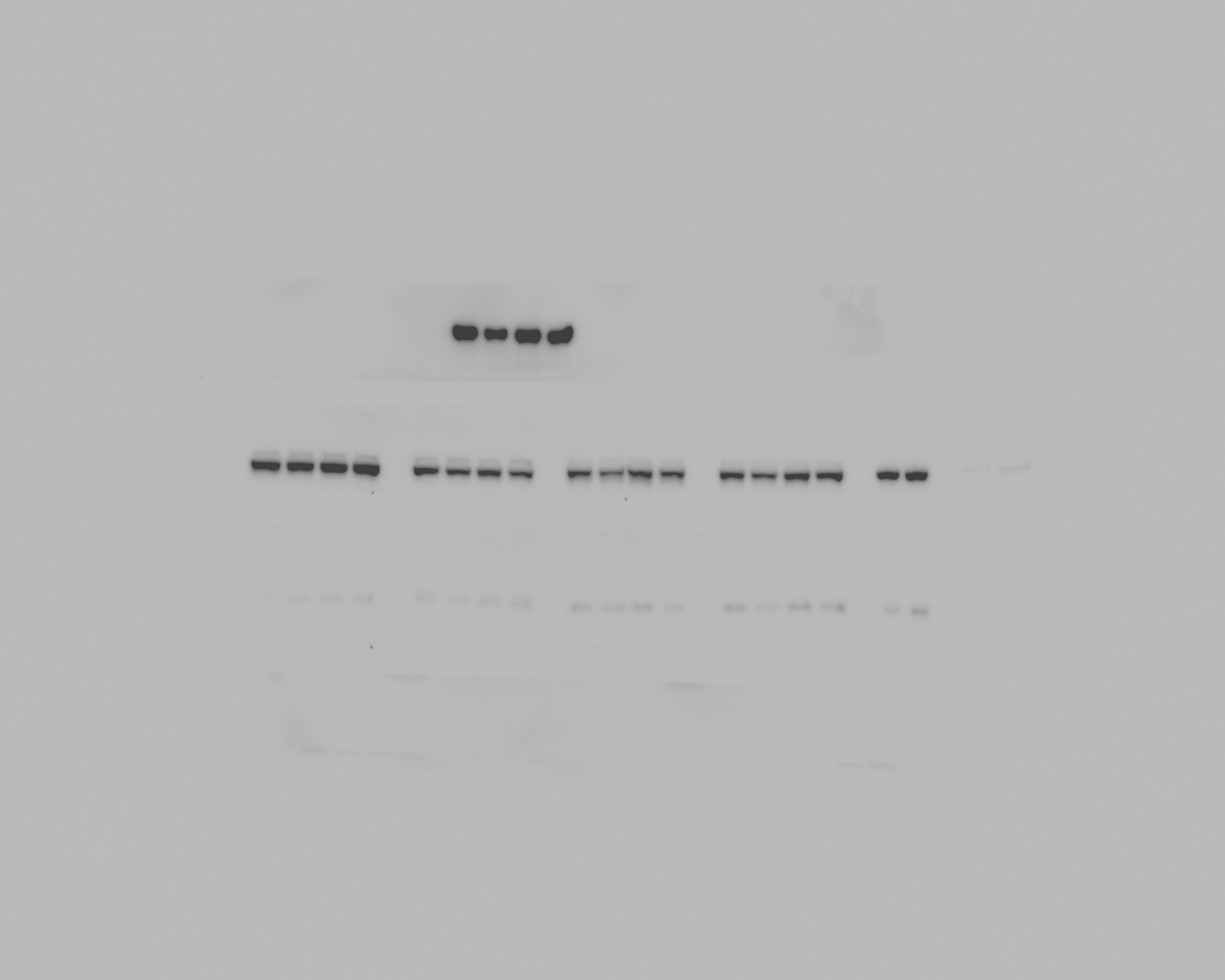

Supplement: Figure 2—figure supplement 1—source data 1. [file elife-106730-fig2-figsupp1-data1.zip › Figure 2ΓÇöfigure supplement 1ΓÇösource data 1/Figure 2ΓÇöfigure supplement 1C/111624-G1_s1618_cul3_p21_Ccna1_04(Chemiluminescence_Background).tif]

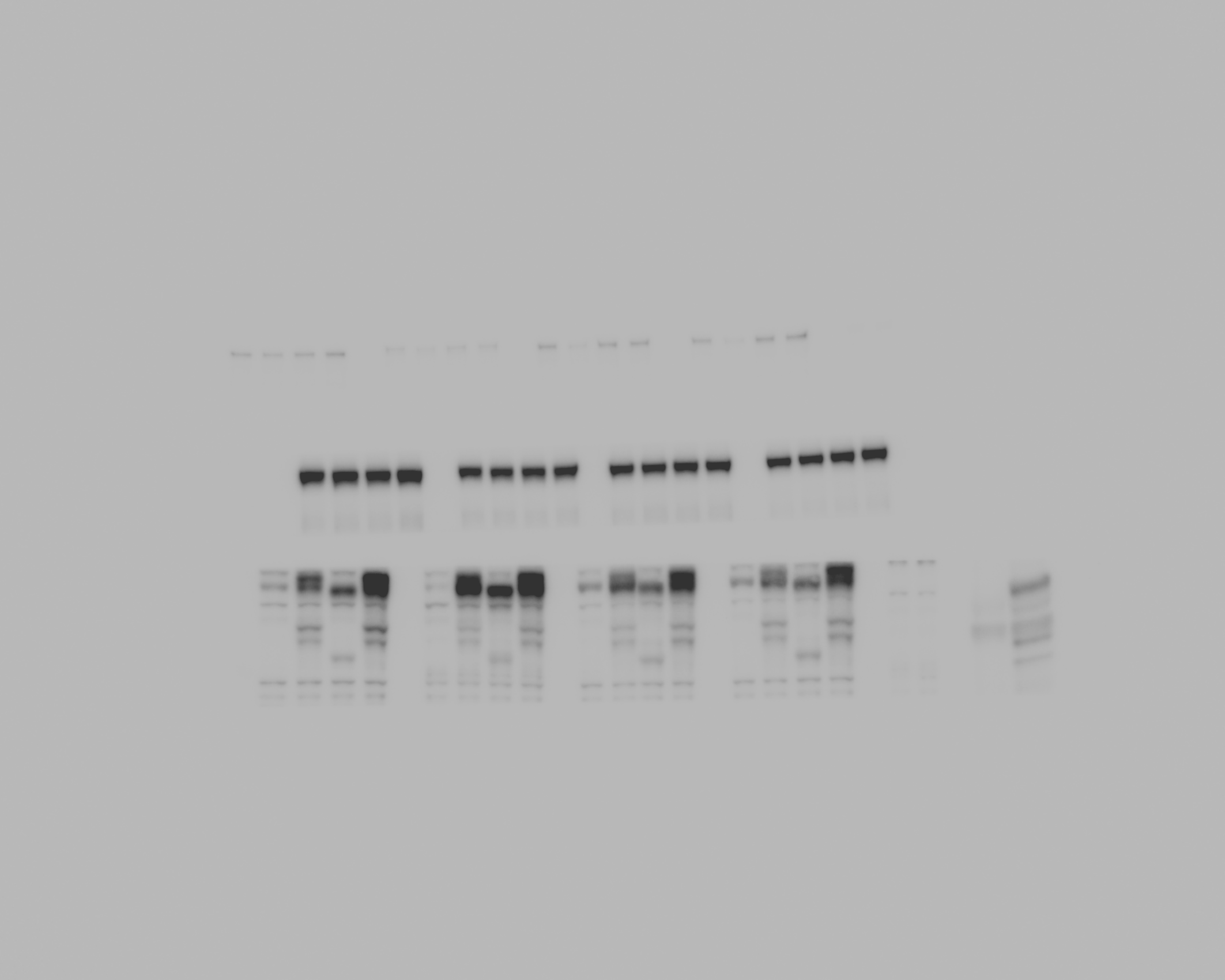

Supplement: Figure 2—figure supplement 1—source data 1. [file elife-106730-fig2-figsupp1-data1.zip › Figure 2ΓÇöfigure supplement 1ΓÇösource data 1/Figure 2ΓÇöfigure supplement 1C/111624-G1_53bp1_usp28_FLAG_CCND1_2(Chemiluminescence_Background).tif]
