# Supplementary figures and images for: GMCL1 controls 53BP1 stability and modulates taxane sensitivity (part 2 of 2)
Source: eLife. 2026 Jan 19;14:RP106730. doi: 10.7554/eLife.106730 (PMC12815461; doi:10.7554/eLife.106730)

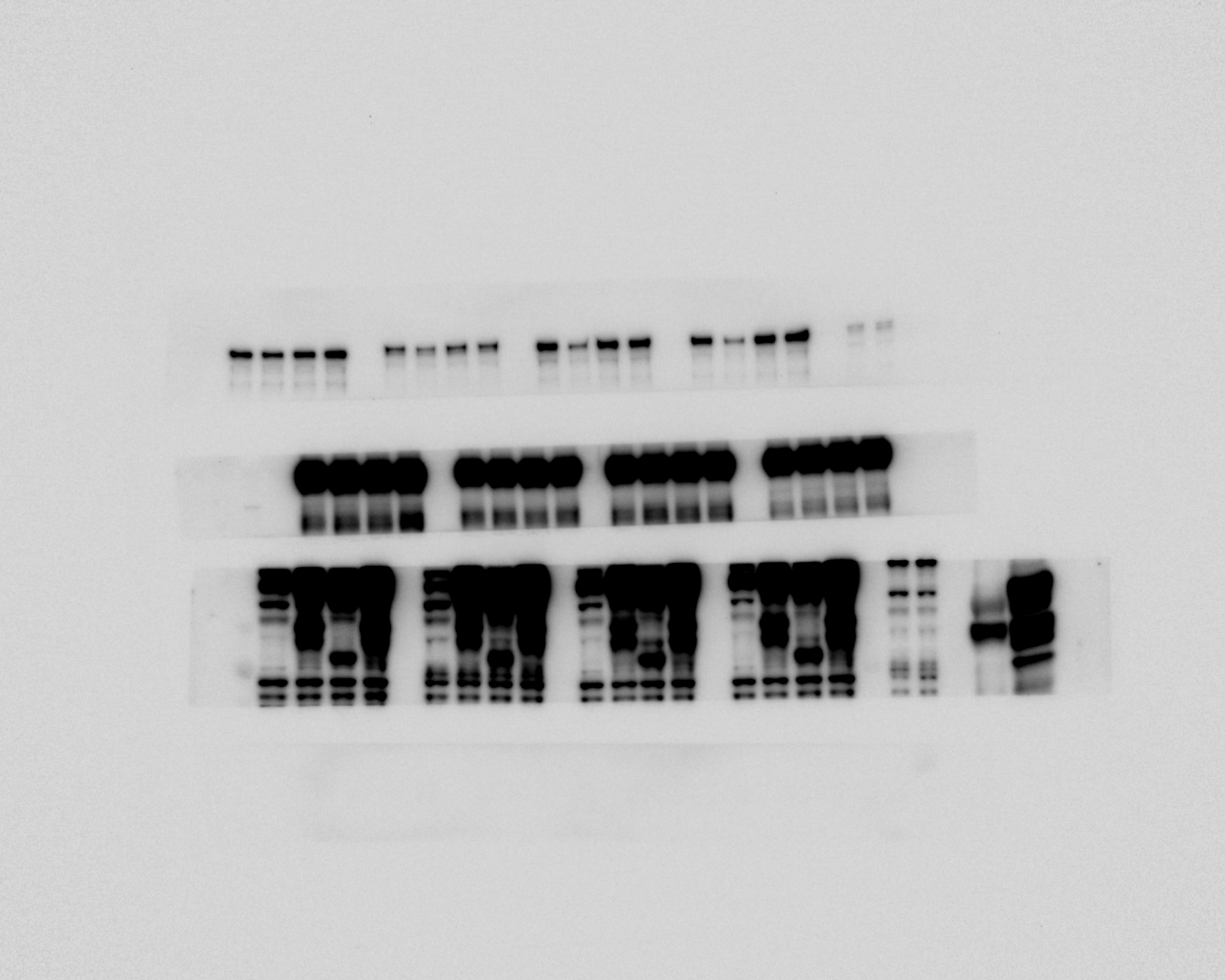

Supplement: Figure 2—figure supplement 1—source data 1. [file elife-106730-fig2-figsupp1-data1.zip › Figure 2ΓÇöfigure supplement 1ΓÇösource data 1/Figure 2ΓÇöfigure supplement 1C/111624-G1_53bp1_usp28_FLAG_CCND1_4(Chemiluminescence).tif]

# Supplemental Figure2C

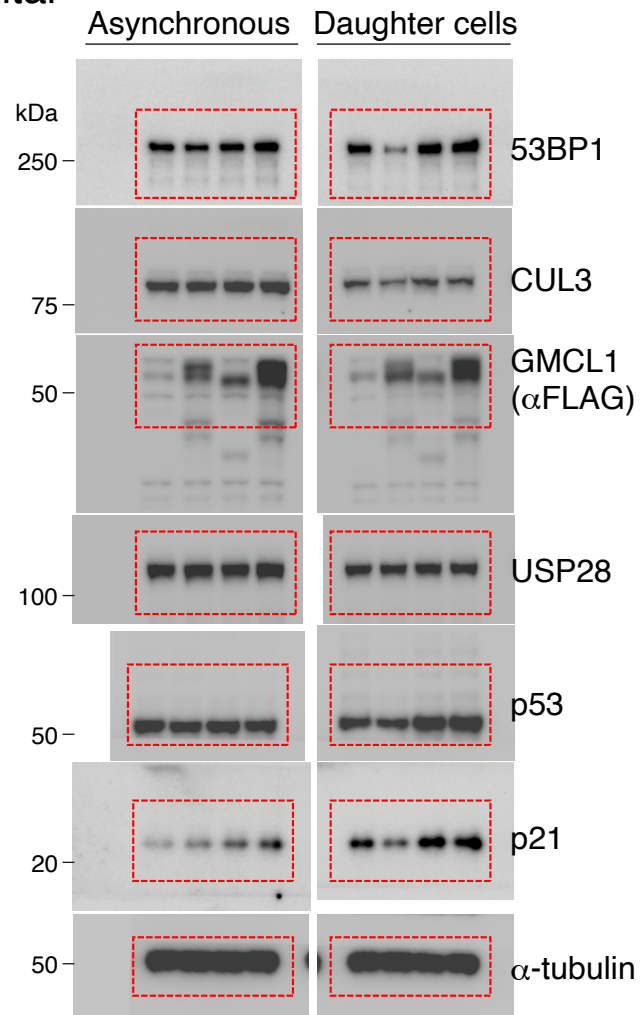

Supplement: Figure 2—figure supplement 1—source data 2. [file elife-106730-fig2-figsupp1-data2.zip › Figure 2ΓÇöfigure supplement 1ΓÇösource data 2/SupFigure2-1_Raw uncropped supporting Western blot files.pdf]

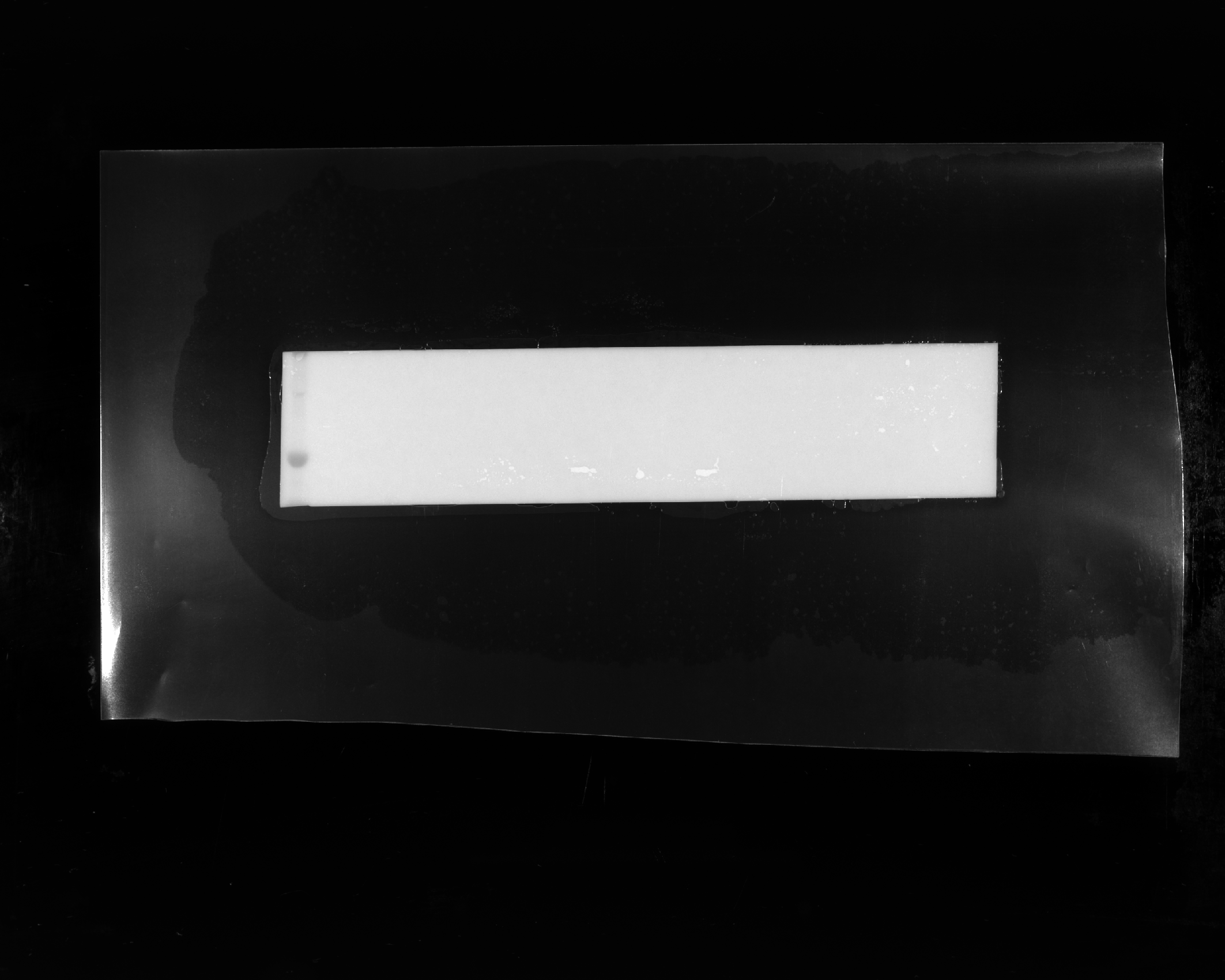

Supplement: Figure 3—source data 1. [file elife-106730-fig3-data1.zip › Figure 3ΓÇösource data 1/Figure 3B/062525-Expression_GMCL1_6(Colorimetric).tif]

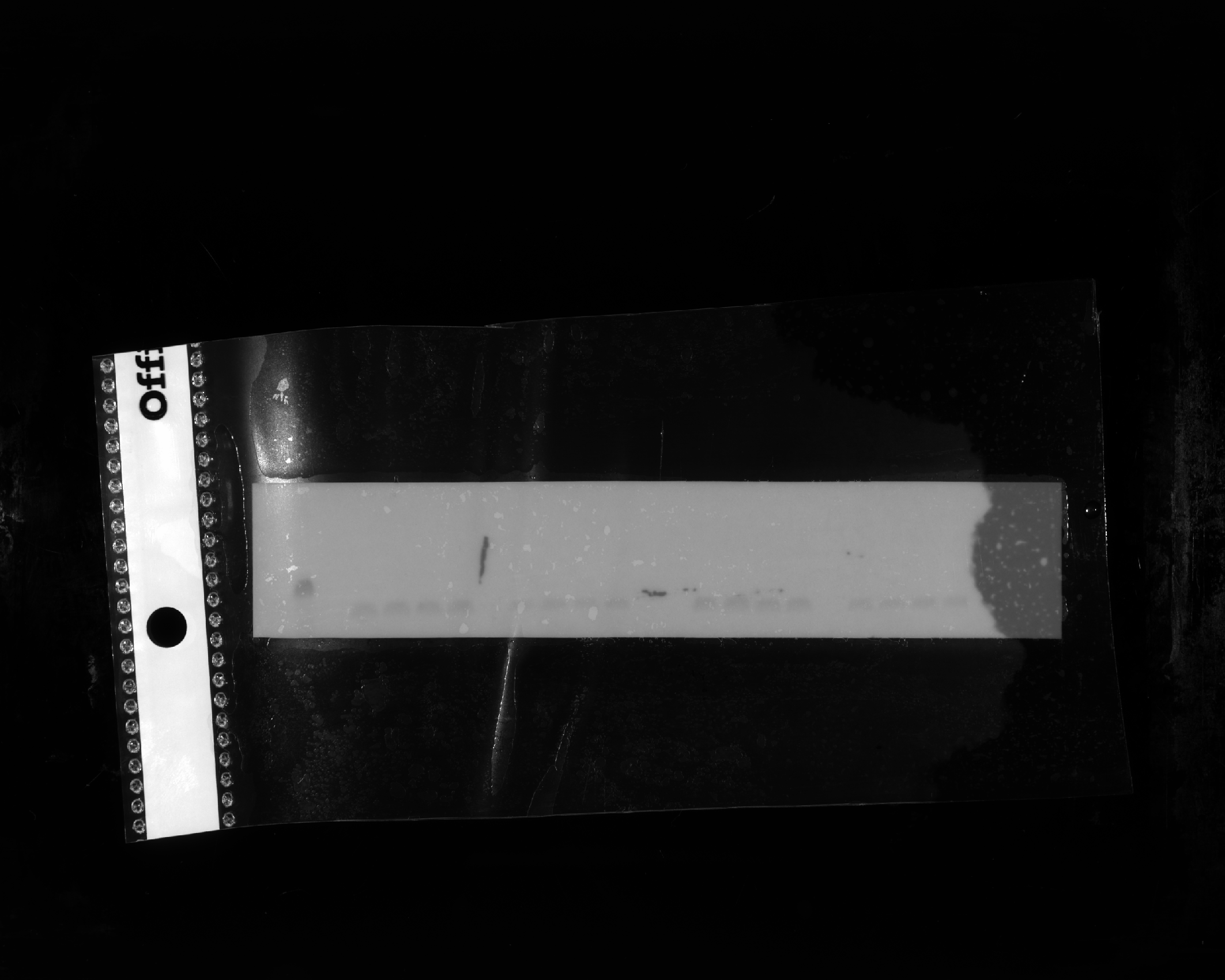

Supplement: Figure 3—source data 1. [file elife-106730-fig3-data1.zip › Figure 3ΓÇösource data 1/Figure 3B/062625-ExpressionCheck_actin_2(Colorimetric).tif]

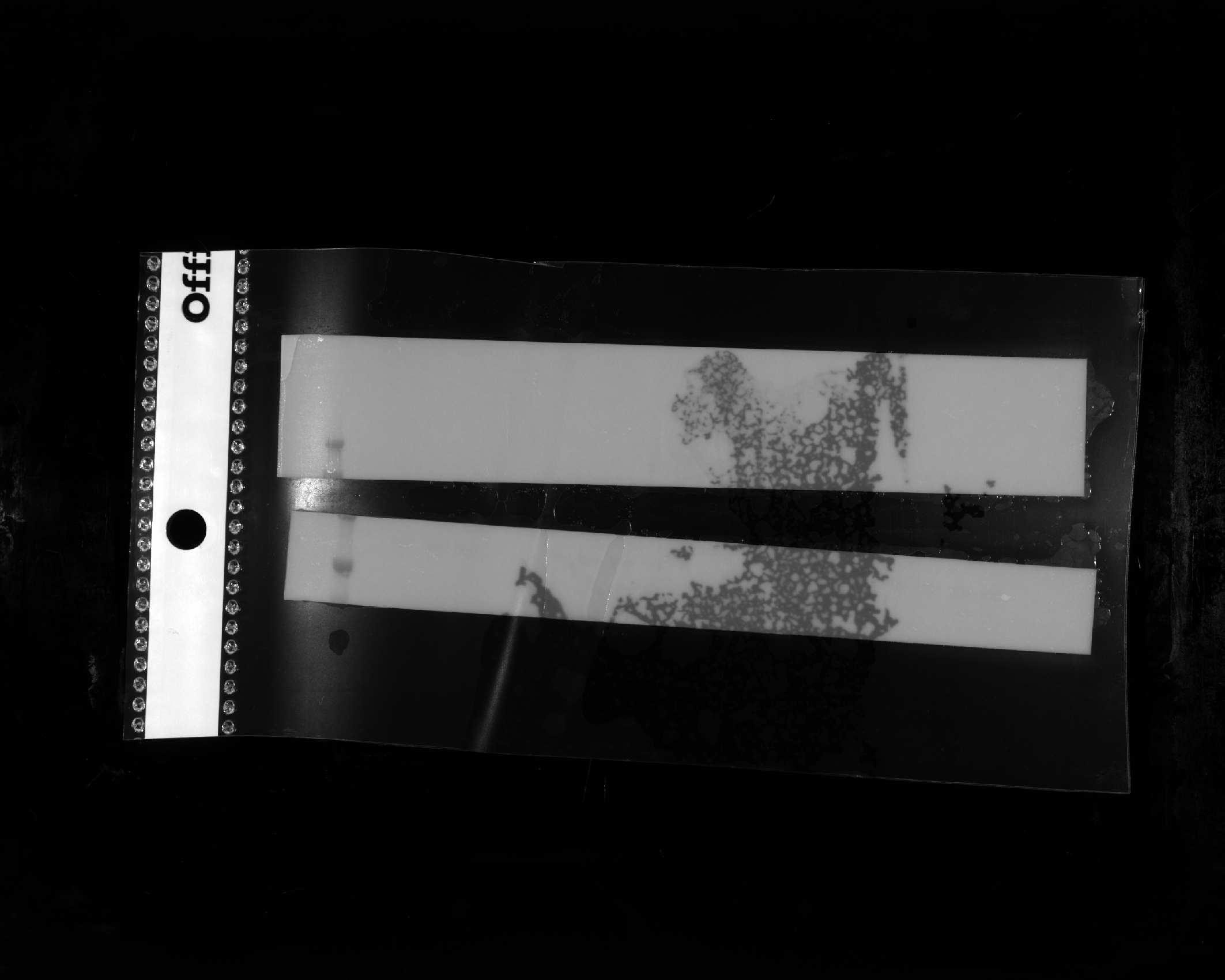

Supplement: Figure 3—source data 1. [file elife-106730-fig3-data1.zip › Figure 3ΓÇösource data 1/Figure 3B/062625-ExpressionCheck_53bp1_Uso28_10(Colorimetric).tif]

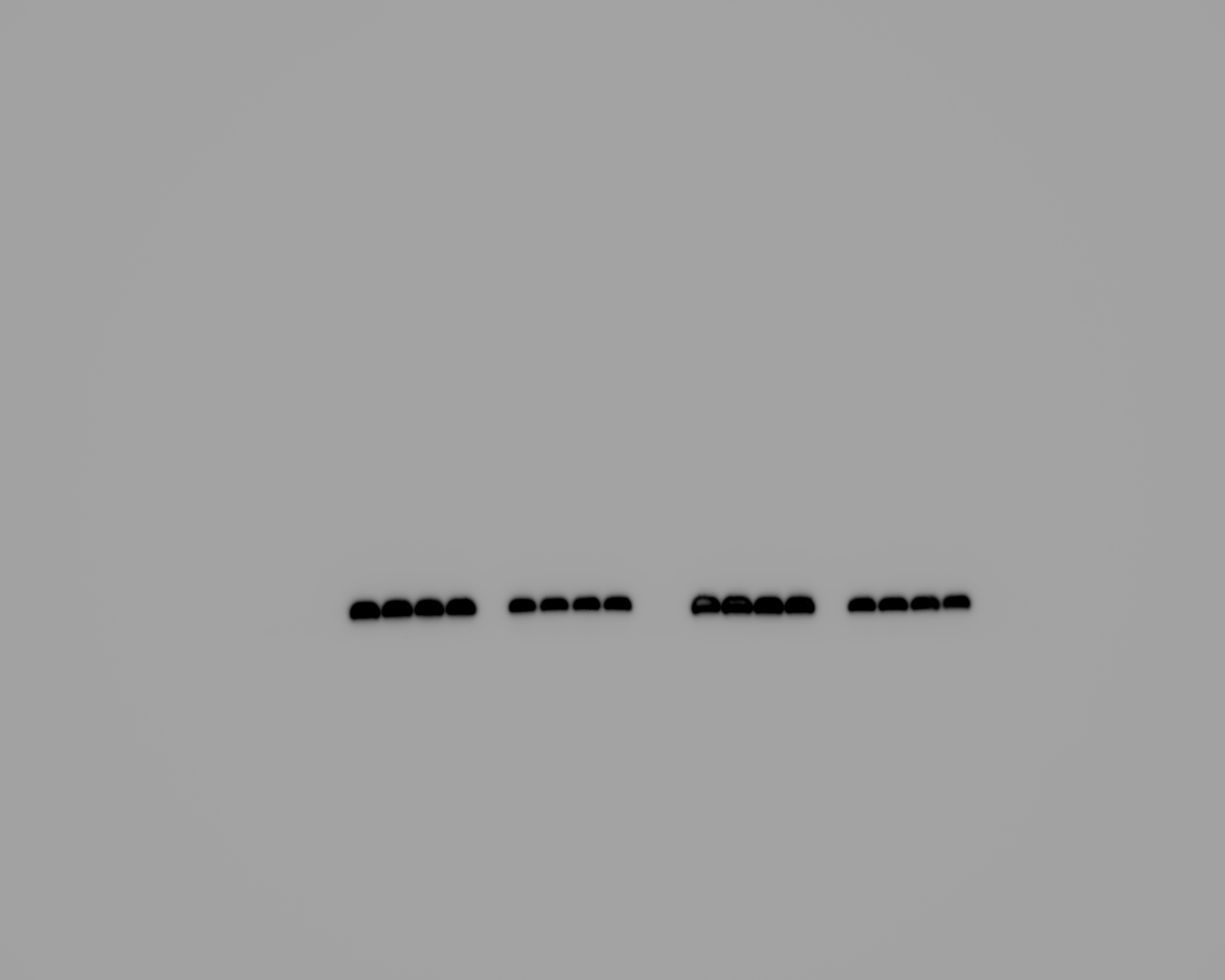

Supplement: Figure 3—source data 1. [file elife-106730-fig3-data1.zip › Figure 3ΓÇösource data 1/Figure 3B/062625-ExpressionCheck_actin_1(Chemiluminescence).tif]

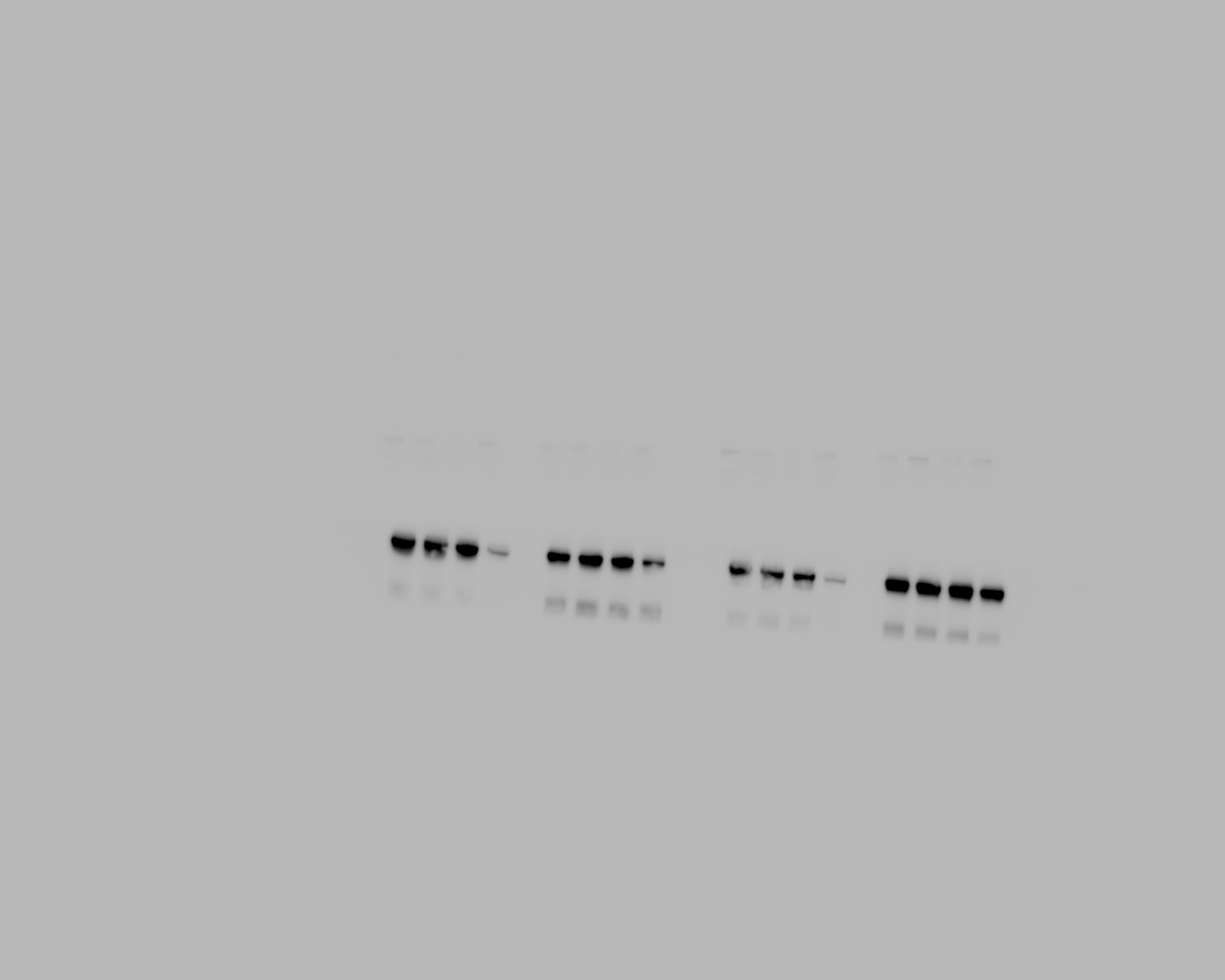

Supplement: Figure 3—source data 1. [file elife-106730-fig3-data1.zip › Figure 3ΓÇösource data 1/Figure 3B/062625-ExpressionCheck_53bp1_Uso28_03(Chemiluminescence).tif]

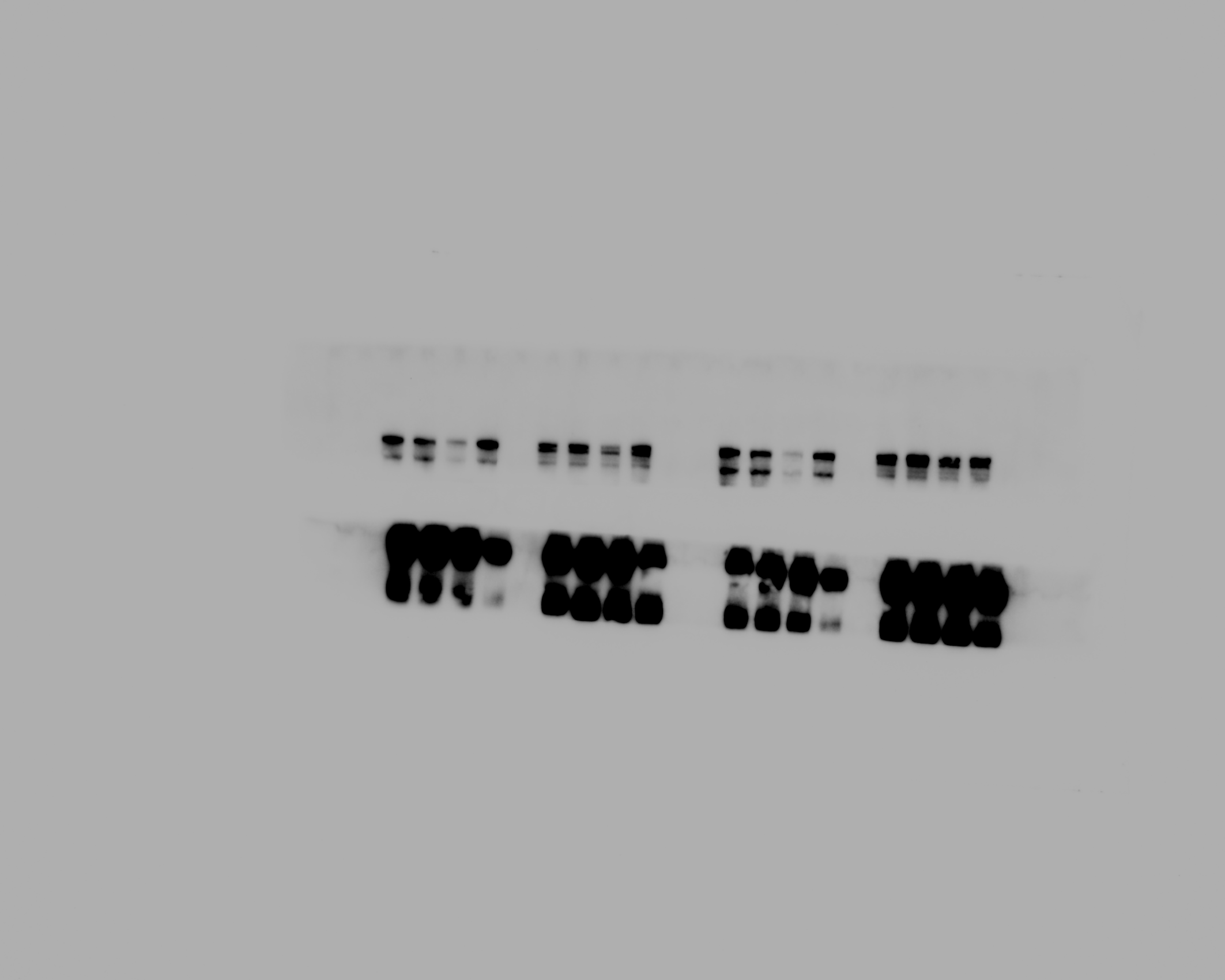

Supplement: Figure 3—source data 1. [file elife-106730-fig3-data1.zip › Figure 3ΓÇösource data 1/Figure 3B/062625-ExpressionCheck_53bp1_Uso28_09(Chemiluminescence).tif]

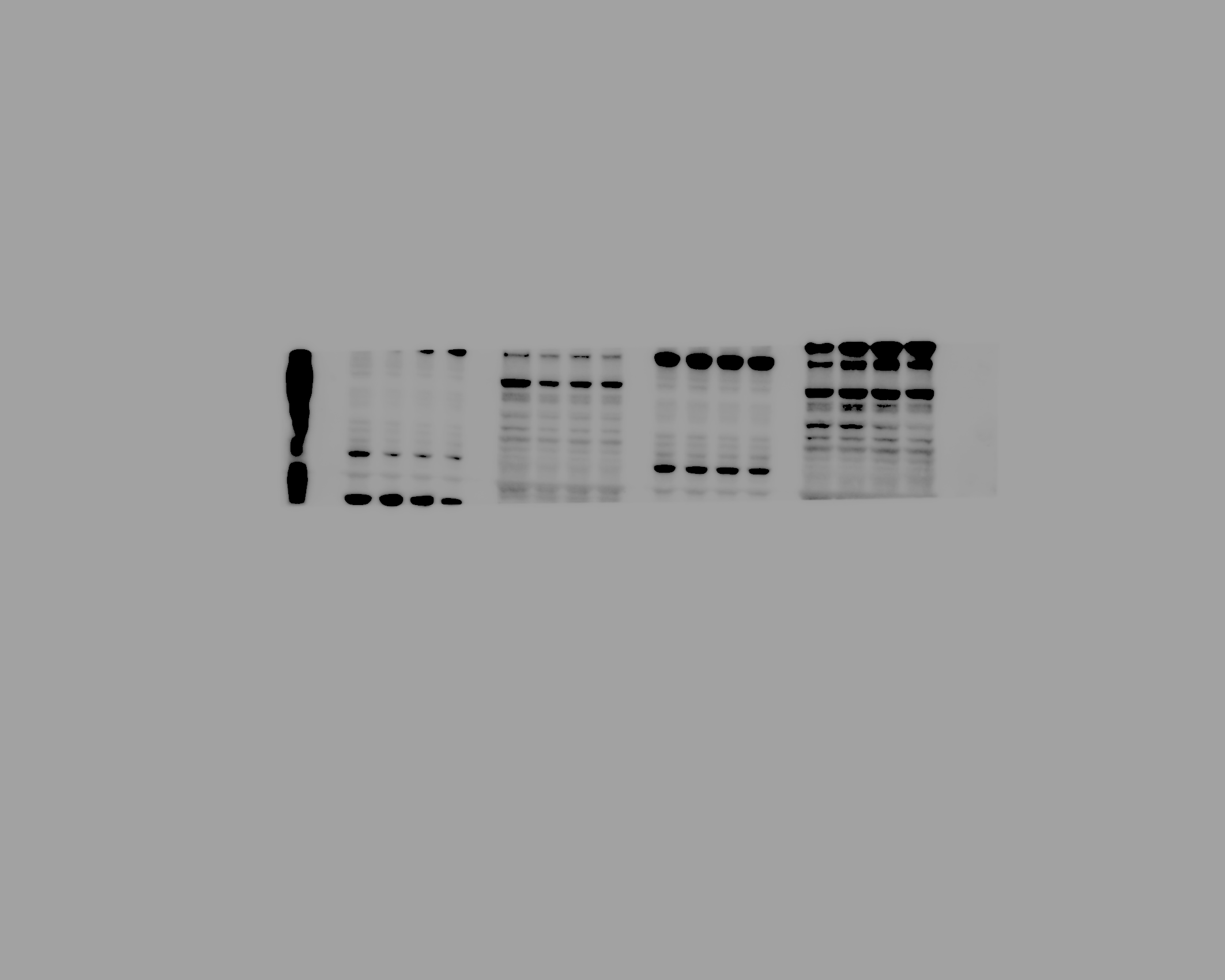

Supplement: Figure 3—source data 1. [file elife-106730-fig3-data1.zip › Figure 3ΓÇösource data 1/Figure 3B/062525-Expression_GMCL1_2(Chemiluminescence).png]

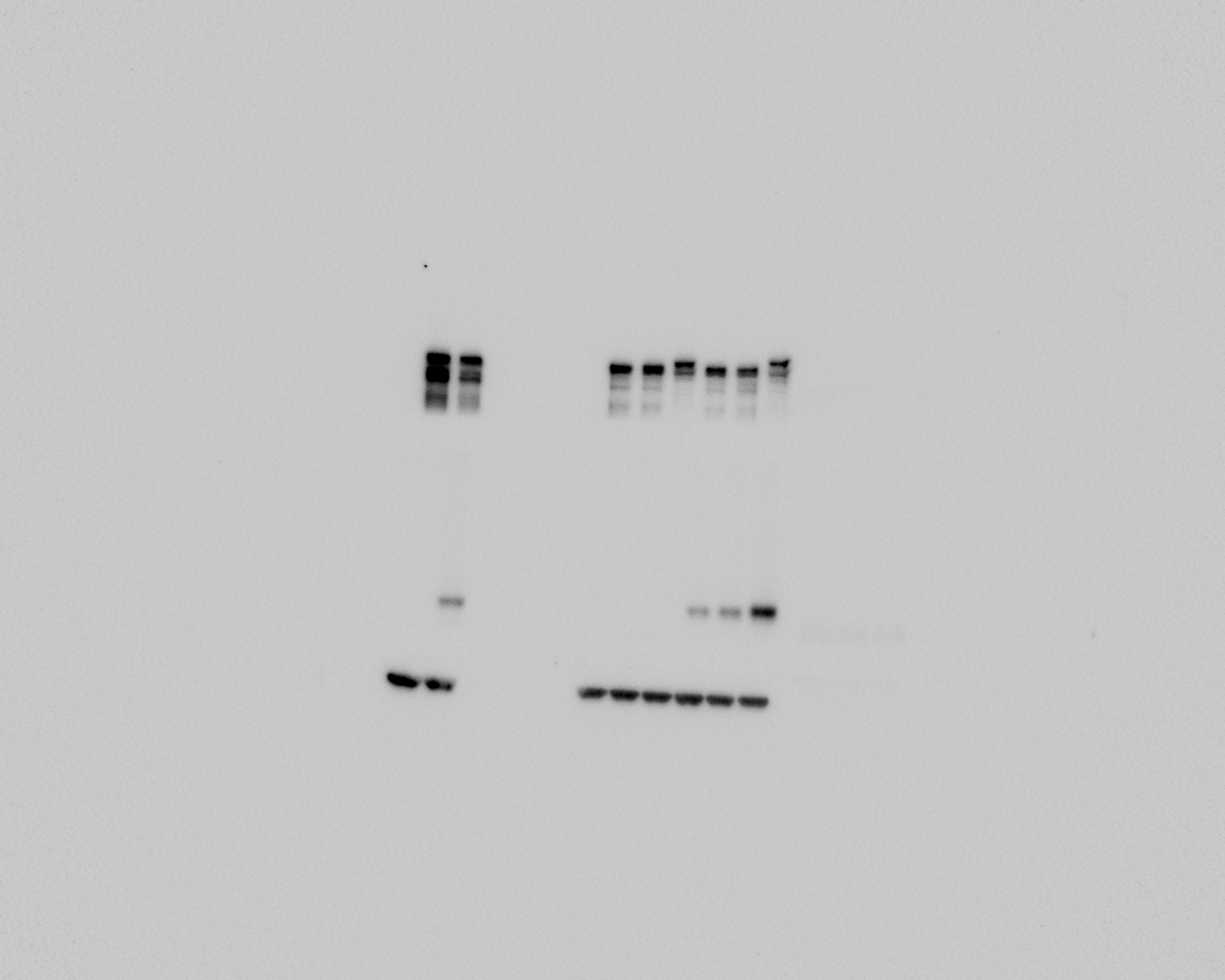

Supplement: Figure 3—source data 1. [file elife-106730-fig3-data1.zip › Figure 3ΓÇösource data 1/Figure 3D/072025-GMCL1V5expression_53bp1_V5_actin_2(Chemiluminescence).tif]

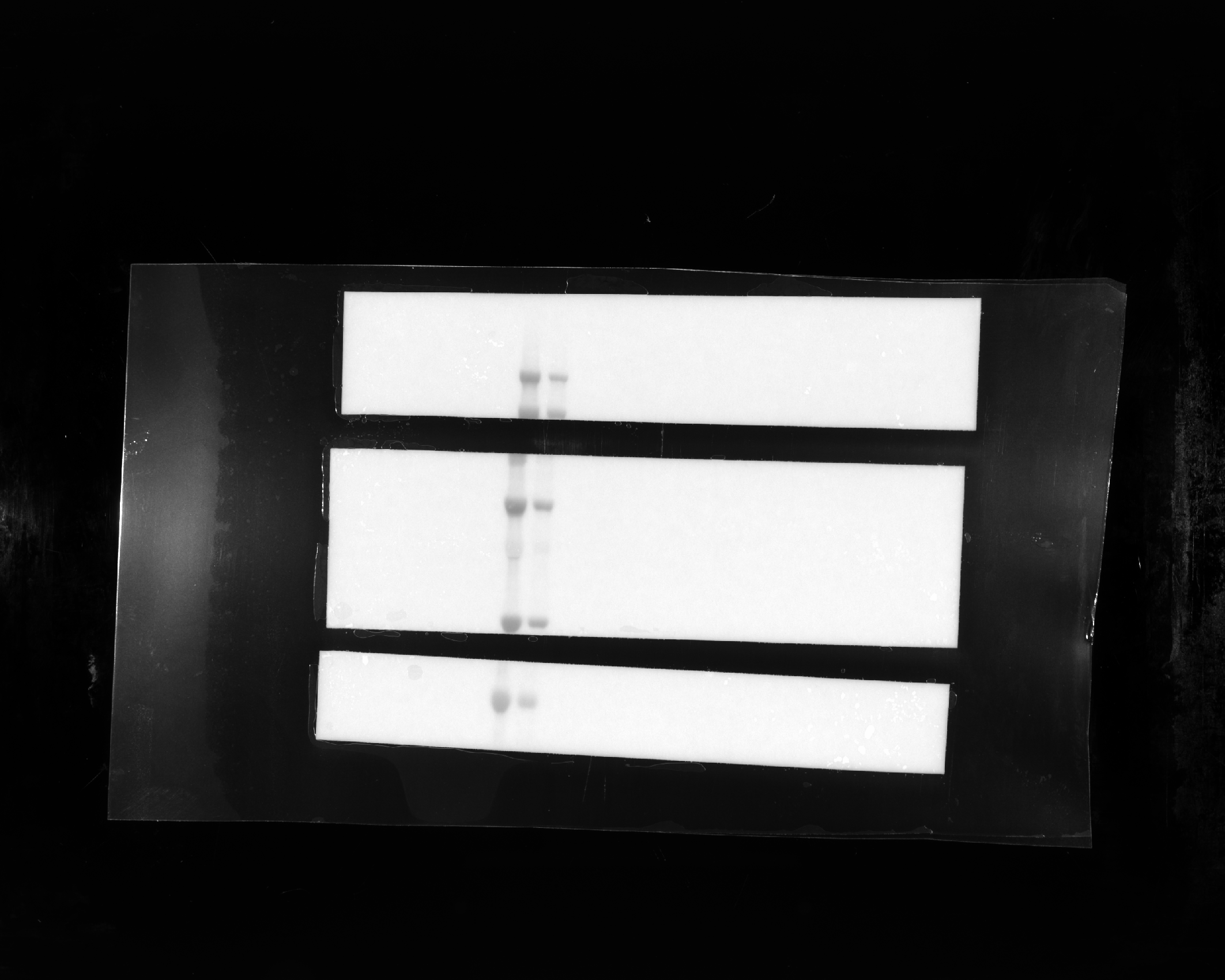

Supplement: Figure 3—source data 1. [file elife-106730-fig3-data1.zip › Figure 3ΓÇösource data 1/Figure 3D/072025-GMCL1V5expression_53bp1_V5_actin_6(Colorimetric).tif]

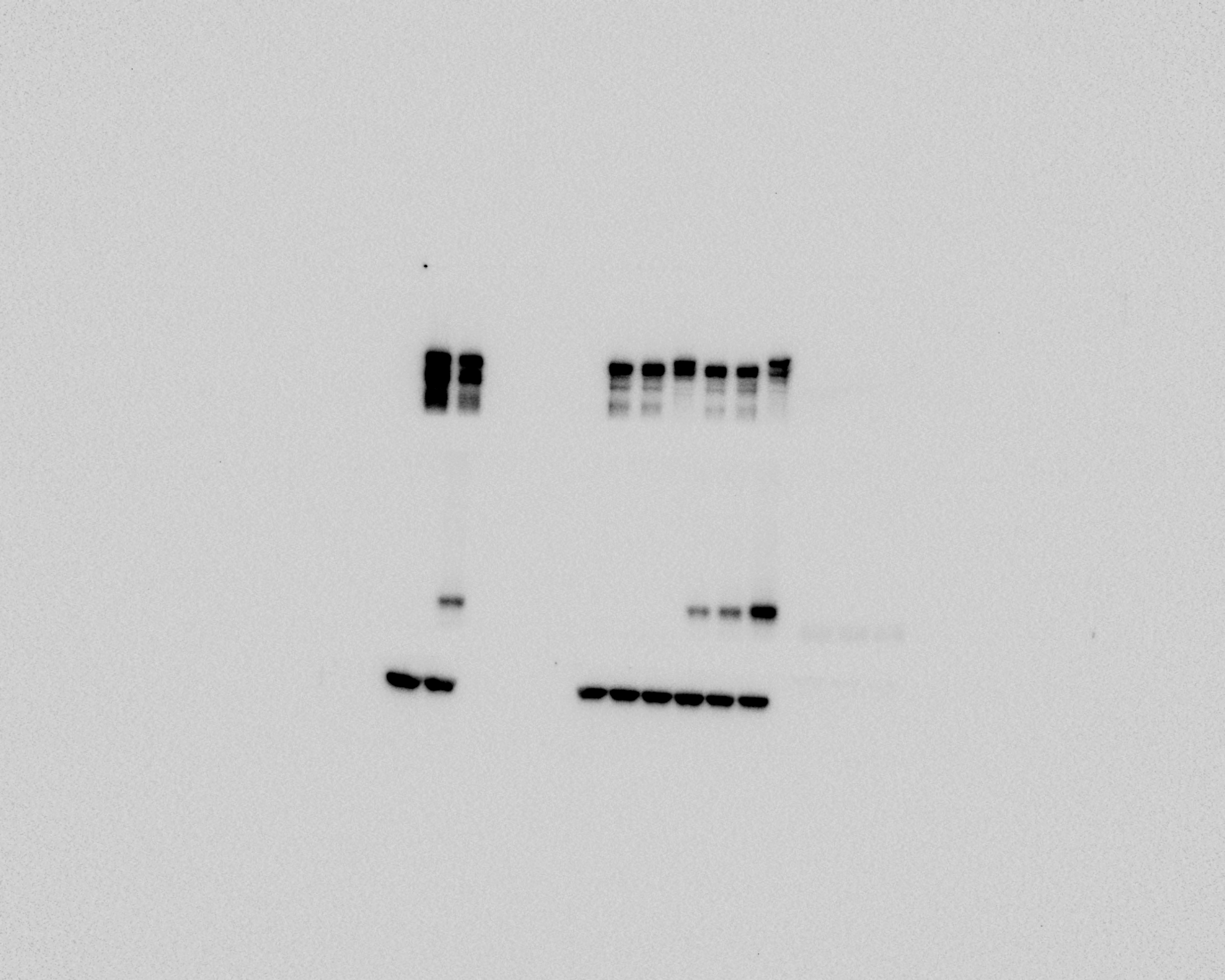

Supplement: Figure 3—source data 1. [file elife-106730-fig3-data1.zip › Figure 3ΓÇösource data 1/Figure 3D/072025-GMCL1V5expression_53bp1_V5_actin_2(Chemiluminescence_Background).tif]

Figure3B

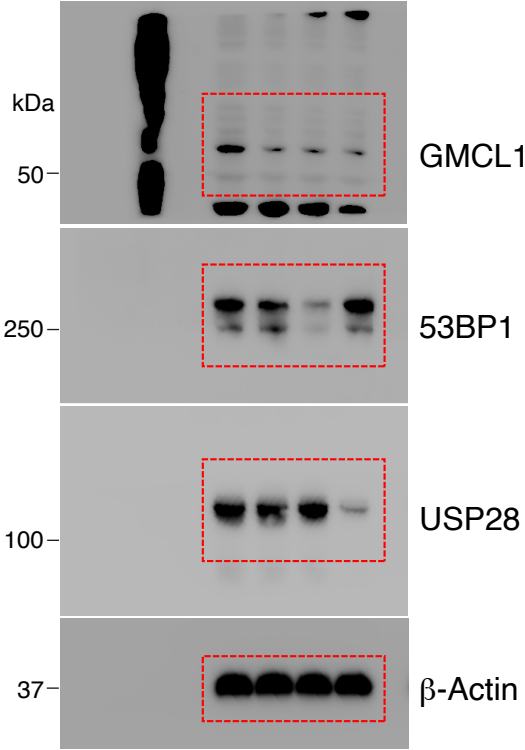

Figure3D

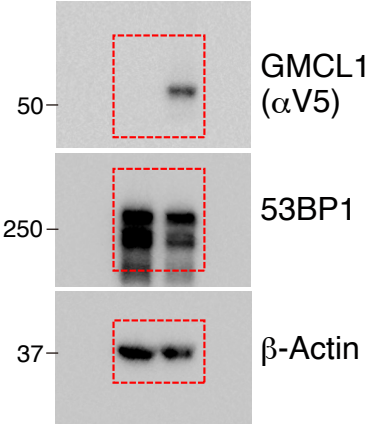

Supplement: Figure 3—source data 2. [file elife-106730-fig3-data2.zip › Figure 3ΓÇösource data 2/Figure3-1_Raw uncropped supporting Western blot files.pdf]
